# Supplementary material for: Sesquiterpenoids from the Florets of Carthamus tinctorius (Safflower) and Their Anti-Atherosclerotic Activity
Source: Nutrients. 2022 Dec 16;14(24):5348. doi: 10.3390/nu14245348 (PMC9783904; doi:10.3390/nu14245348)
Supplement: Supplementary file 1 [file nutrients-14-05348-s001.zip › nutrients-2028487-supplementary.pdf]

Supplementary data

Sesquiterpenoids from the florets of *Carthamus tinctorius* (safflower) and their anti-atherosclerotic activity

Lei Li [1,2,3,†], Juan Liu [1,2,3,†], Xinrui Li [1,2,3], Yuqin Guo [1,2,3], Yunqiu Fan [1,2,3], Hongzhen Shu [1,2,3], Guangxu Wu [1,2,3], Cheng Peng [1,2,*] and Liang Xiong [1,2,3,*]

[1] State Key Laboratory of Southwestern Chinese Medicine Resources, Chengdu University of Traditional Chinese Medicine, Chengdu 611137, China

[2] School of Pharmacy, Chengdu University of Traditional Chinese Medicine, Chengdu 611137, China

[3] Institute of Innovative Medicine Ingredients of Southwest Specialty Medicinal Materials, Chengdu University of Traditional Chinese Medicine, Chengdu 611137, China

* Correspondence: pengcheng@cdutcm.edu.cn (C.P.); xiling0505@126.com (L.X.)

† These authors made equal contributions to this work.

List of Contents

No.	Content	Page
1	**Text S1. General experimental details**	S1
2	Text S2. **ECD calculation of compound 1**	S1
3	**Figure S1.** *ω*B97XD/DGDZVP optimized 21 conformers of (1*R*,4*S*,9*S*,11*R*)-**1**	S3
4	**Table S1.** Energy analysis for the conformers of (1*R*,4*S*,9*S*,11*R*)-**1**	S3
5	**Text S3. ECD calculation of compound 2**	S4
6	**Figure S2.** *ω*B97XD/DGDZVP optimized 18 conformers of (1*R*,4*R*,9*S*,11*R*)-**2**	S5
7	**Table S2.** Energy analysis for the conformers of (1*R*,4*R*,9*S*,11*R*)-**2**	S5
8	**Text S4. ECD calculation of compound 3a**	S6
9	**Figure S3.** *ω*B97XD/DGDZVP optimized 9 conformers of (1*R*,5*S*,8*S*,9*S*,11*R*)-**3a**	S7
10	**Table S3.** Energy analysis for the conformers of (1*R*,5*S*,8*S*,9*S*,11*R*)-**3a**	S7
11	**Text S5. ECD calculation of compound 4a**	S8
12	**Figure S4.** *ω*B97XD/DGDZVP optimized 8 conformers of (1*S*,7*R*,10*S*)-**4a**	S9
13	**Table S4.** Energy analysis for the conformers of (1*S*,7*R*,10*S*)-**4a**	S9
14	**Text S6. ECD calculation of compound 5a**	S9
15	**Figure S5.** *ω*B97XD/DGDZVP optimized 15 conformers of (2*R*,5*R*,10*R*)-**5a**	S10
16	**Table S5.** Energy analysis for the conformers of (2*R*,5*R*,10*R*)-**5a**	S10
17	**References**	S11
18	**Figure S6.** The ^{1}H NMR spectrum of compound **1** in CD_3OD	S13
19	**Figure S7.** The ^{13}C NMR spectrum of compound **1** in CD_3OD	S14
20	**Figure S8.** The DEPT spectrum of compound **1** in CD_3OD	S15
21	**Figure S9.** The HSQC spectrum of compound **1** in CD_3OD	S16
22	**Figure S10.** The ^{1}H-^{1}H COSY spectrum of compound **1** in CD_3OD	S17
23	**Figure S11.** The HMBC spectrum of compound **1** in CD_3OD	S18
24	**Figure S12.** The NOESY spectrum of compound **1** in CD_3OD	S19
25	**Figure S13.** The IR spectrum of compound **1**	S20
26	**Figure S14.** The (+)-HR-ESI-MS spectroscopic data of compound **1**	S21
27	**Figure S15.** The ^{1}H NMR spectrum of compound **2** in CD_3OD	S22
28	**Figure S16.** The ^{13}C NMR spectrum of compound **2** in CD_3OD	S23
29	**Figure S17.** The DEPT spectrum of compound **2** in CD_3OD	S24
30	**Figure S18.** The HSQC spectrum of compound **2** in CD_3OD	S25
31	**Figure S19.** The ^{1}H-^{1}H COSY spectrum of compound **2** in CD_3OD	S26
32	**Figure S20.** The HMBC spectrum of compound **2** in CD_3OD	S27
33	**Figure S21.** The NOESY spectrum of compound **2** in CD_3OD	S28
34	**Figure S22.** The 1D NOE spectrum of compound **2** in CD_3OD	S29
35	**Figure S23.** The IR spectrum of compound **2**	S30
36	**Figure S24.** The (+)-HR-ESI-MS spectroscopic data of compound **2**	S31
37	**Figure S25.** The ^{1}H NMR spectrum of compound **3** in CD_3OD	S32
38	**Figure S26.** The ^{13}C NMR spectrum of compound **3** in CD_3OD	S33
39	**Figure S27.** The DEPT spectrum of compound **3** in CD_3OD	S34
40	**Figure S28.** The HSQC spectrum of compound **3** in CD_3OD	S35

41	**Figure S29.** The ^{1}H-^{1}H COSY spectrum of compound **3** in CD_3OD	S36
42	**Figure S30.** The HMBC spectrum of compound **3** in CD_3OD	S37
43	**Figure S31.** The NOESY spectrum of compound **3** in CD_3OD	S38
44	**Figure S32.** The 1D NOE spectrum of compound **3** in CD_3OD	S39
45	**Figure S33.** The IR spectrum of compound **3**	S40
46	**Figure S34.** The (+)-HR-ESI-MS spectroscopic data of compound **3**	S41
47	**Figure S35.** The ^{1}H NMR spectrum of compound **3a** in CD_3OD	S42
48	**Figure S36.** The (+)-HR-ESI-MS spectroscopic data of compound **3a**	S43
49	**Figure S37.** The ^{1}H NMR spectrum of compound **4** in CD_3OD	S44
50	**Figure S38.** The ^{13}C NMR spectrum of compound **4** in CD_3OD	S45
51	**Figure S39.** The DEPT spectrum of compound **4** in CD_3OD	S46
52	**Figure S40.** The HSQC spectrum of compound **4** in CD_3OD	S47
53	**Figure S41.** The ^{1}H-^{1}H COSY spectrum of compound **4** in CD_3OD	S48
54	**Figure S42.** The HMBC spectrum of compound **4** in CD_3OD	S49
55	**Figure S43.** The NOESY spectrum of compound **4** in CD_3OD	S50
56	**Figure S44.** The IR spectrum of compound **4**	S51
57	**Figure S45.** The (+)-HR-ESI-MS spectroscopic data of compound **4**	S52
58	**Figure S46.** The ^{1}H NMR spectrum of compound **4a** in CD_3OD	S53
59	**Figure S47.** The (+)-HR-ESI-MS spectroscopic data of compound **4a**	S54
60	**Figure S48.** The ^{1}H NMR spectrum of compound **5** in CD_3OD	S55
61	**Figure S49.** The ^{13}C NMR spectrum of compound **5** in CD_3OD	S56
62	**Figure S50.** The DEPT spectrum of compound **5** in CD_3OD	S57
63	**Figure S51.** The HSQC spectrum of compound **5** in CD_3OD	S58
64	**Figure S52.** The ^{1}H-^{1}H COSY spectrum of compound **5** in CD_3OD	S59
65	**Figure S53.** The HMBC spectrum of compound **5** in CD_3OD	S60
66	**Figure S54.** The NOESY spectrum of compound **5** in CD_3OD	S61
67	**Figure S55.** The IR spectrum of compound **5**	S62
68	**Figure S56.** The (+)-HR-ESI-MS spectroscopic data of compound **5**	S63
69	**Figure S57.** The ^{1}H NMR spectrum of compound **5a** in CD_3OD	S64
70	**Figure S58.** The (+)-HR-ESI-MS spectroscopic data of compound **5a**	S65

Text S1. General experimental details.

NMR spectra were recorded on a Bruker AVANCE NEO 600 instrument (Bruker Corporation, MA, USA) with the solvent peaks as reference standards. HR-ESI-MS analyses were measured on a Bruker timsTOF-MS instrument (Bruker Corporation, MA, USA). IR spectra were obtained on a Spectrum One FT-IR spectrometer (Perki-nelmer Las Inc., CA, USA). ECD spectra were measured by an Applied Photophysics Chirascan and Chirascan-plus circular dichroism spectrometer (Applied Photophysics Ltd., Leatherhead, England). Optical rotations were recorded by an Anton Paar MCP 200 polarimeter (Anton Paar GmbH, Graz, Austria). Absorbance was measured with a Molecular Devices SpectraMax iD3 microplate reader (Molecular Devices, LLC., CA, USA). Cells were observed with a Leica DMi1 Inverted microscope (Leica Microsystems GmbH, Wetzlar, Germany).

Text S2. ECD calculation of compound 1.

ECD calculation of (1*R*, 4*S*, 9*S*, 11*R*)-1 Conformation searches based on molecular mechanics with MMFF94s force field were performed for (1*R*, 4*S*, 9*S*, 11*R*)-**1** and gave 21 conformers with relative energies within 2 kcal/mol [1]. The selected conformers were optimized using DFT at the B3LYP/6-31G (d) level in a vacuum with the Gaussian 16 program (Table S1) [2]. The B3LYP/6-31G (d)-optimized conformers (Boltzmann distribution ≥ 1%) were then reoptimized at the *ω*B97XD/DGDZVP level in acetonitrile. ECD computations for the *ω*B97XD/DGDZVP-optimized conformers (Figure S1) were conducted at the CAM-B3LYP/DGDZVP level in acetonitrile [3]. According to the Boltzmann distribution theory and the relative Gibbs free energy (ΔG), the ECD spectrum for (1*R*, 4*S*, 9*S*, 11*R*)-**1** was generated using SpecDis 1.71 with σ = 0.3 eV and a UV shift of +13 nm [4]. The results showed that the theoretically calculated ECD spectrum of (1*R*, 4*S*, 9*S*, 11*R*)-**1** was in good agreement with the experimental ECD spectrum of **1**.

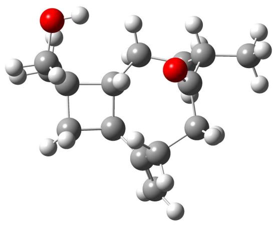

1C1

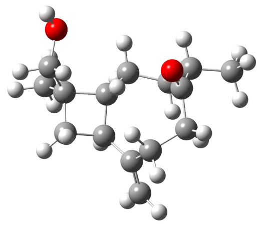

1C2

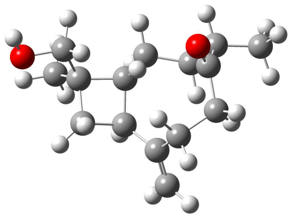

1C3

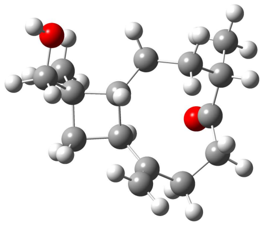

1C4

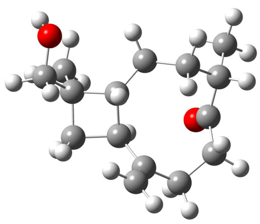

1C5

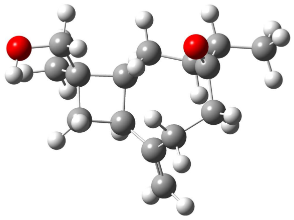

1C6

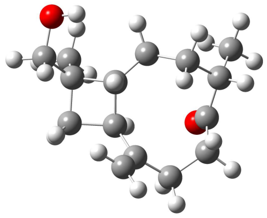

1C7

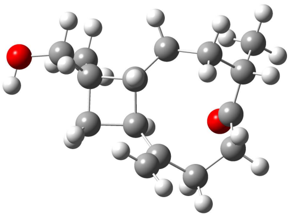

1C8

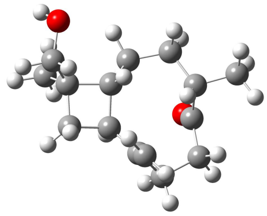

1C9

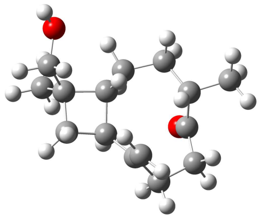

1C10

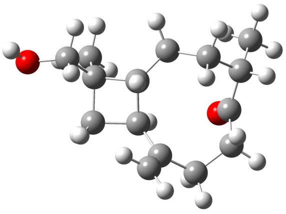

1C11

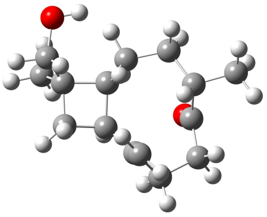

1C12

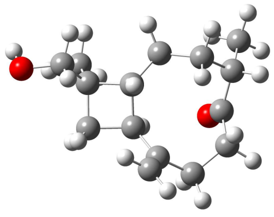

1C13

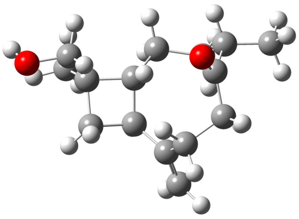

1C14

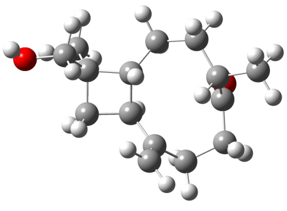

1C15

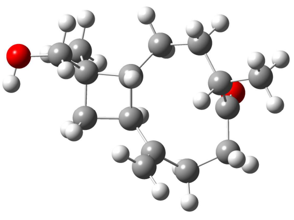

1C16

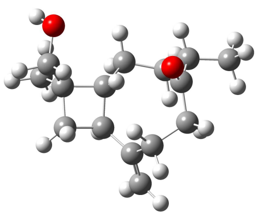

1C17

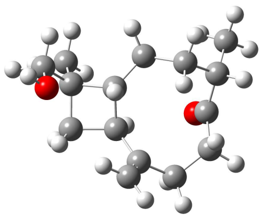

1C18

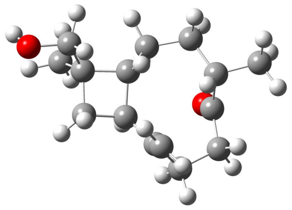

1C19

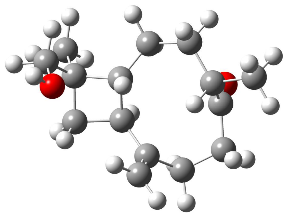

1C20

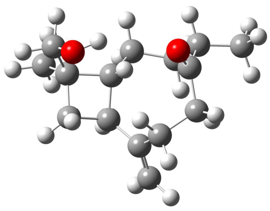

1C21

Figure S1. *ω*B97XD/DGDZVP optimized 21 conformers of (1*R*, 4*S*, 9*S*, 11*R*)-**1**.

Table S1. Energy analysis for the conformers of (1*R*, 4*S*, 9*S*, 11*R*)-**1**.

Conf.	MMFF energy	B3LYP/6-31G(d) Gibbs free energy (298.15 K)			*ω*B97XD/DGDZVP Gibbs free energy (298.15 K)		
	ΔE (Kcal/mol)	G (Hartree)	ΔG (Kcal/mol)	Boltzmann distribution	G (Hartree)	ΔG (Kcal/mol)	Boltzmann distribution
**1C1**	0.00	-736.120131	0.0000	0.038	-735.993738	0.0000	0.114
**1C2**	0.16	-736.119346	0.4930	0.017	-735.99282	0.5760	0.043
**1C3**	0.40	-736.118774	0.8520	0.009	-735.992528	0.7590	0.031
**1C4**	0.47	-736.120513	-0.2400	0.057	-735.992023	1.0760	0.018
**1C5**	0.51	-736.120747	-0.3870	0.073	-735.992547	0.7470	0.032
**1C6**	0.59	-736.119514	0.3870	0.02	-735.993129	0.3820	0.06
**1C7**	0.76	-736.120614	-0.3030	0.063	-735.992599	0.7150	0.034
**1C8**	0.87	-736.120654	-0.3280	0.066	-735.993147	0.3710	0.061
**1C9**	0.92	-736.119893	0.1490	0.03	-735.992893	0.5300	0.046
**1C10**	0.95	-736.119634	0.3120	0.022	-735.992012	1.0830	0.018
**1C11**	1.08	-736.119532	0.3760	0.02	-735.992223	0.9510	0.023
**1C12**	1.10	-736.119958	0.1090	0.032	-735.992869	0.5450	0.045
**1C13**	1.14	-736.11987	0.1640	0.029	-735.992499	0.7770	0.031
**1C14**	1.25	-736.118636	0.9380	0.008	-735.992687	0.6600	0.037
**1C15**	1.26	-736.11911	0.6410	0.013	-735.992166	0.9860	0.021
**1C16**	1.28	-736.120036	0.0600	0.034	-735.993091	0.4060	0.057
**1C17**	1.29	-736.118713	0.8900	0.008	-735.993125	0.3850	0.059
**1C18**	1.36	-736.121206	-0.6750	0.119	-735.992631	0.6950	0.035

**1C19**	1.37	-736.119432	0.4390	0.018	-735.99256	0.7390	0.033
**1C20**	1.47	-736.120841	-0.4460	0.081	-735.99252	0.7640	0.031
**1C21**	1.64	-736.121888	-1.1030	0.244	-735.99412	-0.2400	0.17

Text S3. ECD calculation of compound 2.

ECD calculation of (1*R*, 4*R*, 9*S*, 11*R*)-2 Conformation searches based on molecular mechanics with MMFF94s force field were performed for (1*R*, 4*R*, 9*S*, 11*R*)-**2** and gave 18 conformers with relative energies within 2 kcal/mol [1]. The selected conformers were optimized using DFT at the B3LYP/6-31G (d) level in a vacuum with the Gaussian 16 program (Table S2) [2]. The B3LYP/6-31G (d)-optimized conformers (Boltzmann distribution ≥ 1%) were then reoptimized at the *ω*B97XD/DGDZVP level in acetonitrile. ECD computations for the *ω*B97XD/DGDZVP-optimized conformers (Figure S2) were conducted at the CAM-B3LYP/DGDZVP level in acetonitrile [3]. According to the Boltzmann distribution theory and the relative Gibbs free energy (ΔG), the ECD spectrum for (1*R*, 4*R*, 9*S*, 11*R*)-**2** was generated using SpecDis 1.71 with σ = 0.3 eV and a UV shift of +15 nm [4]. The results showed that the theoretically ECD spectrum of (1*R*, 4*R*, 9*S*, 11*R*)-**2** was in good agreement with the experimental ECD spectrum of **2**.

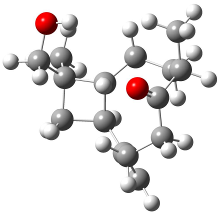

2C1

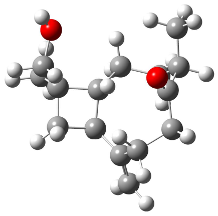

2C2

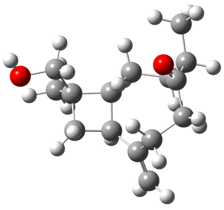

2C3

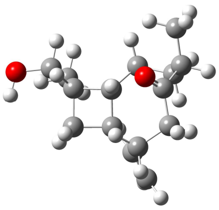

2C4

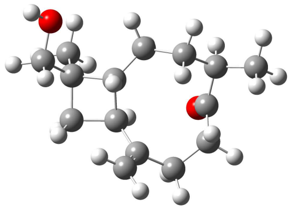

2C5

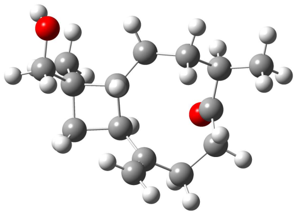

2C6

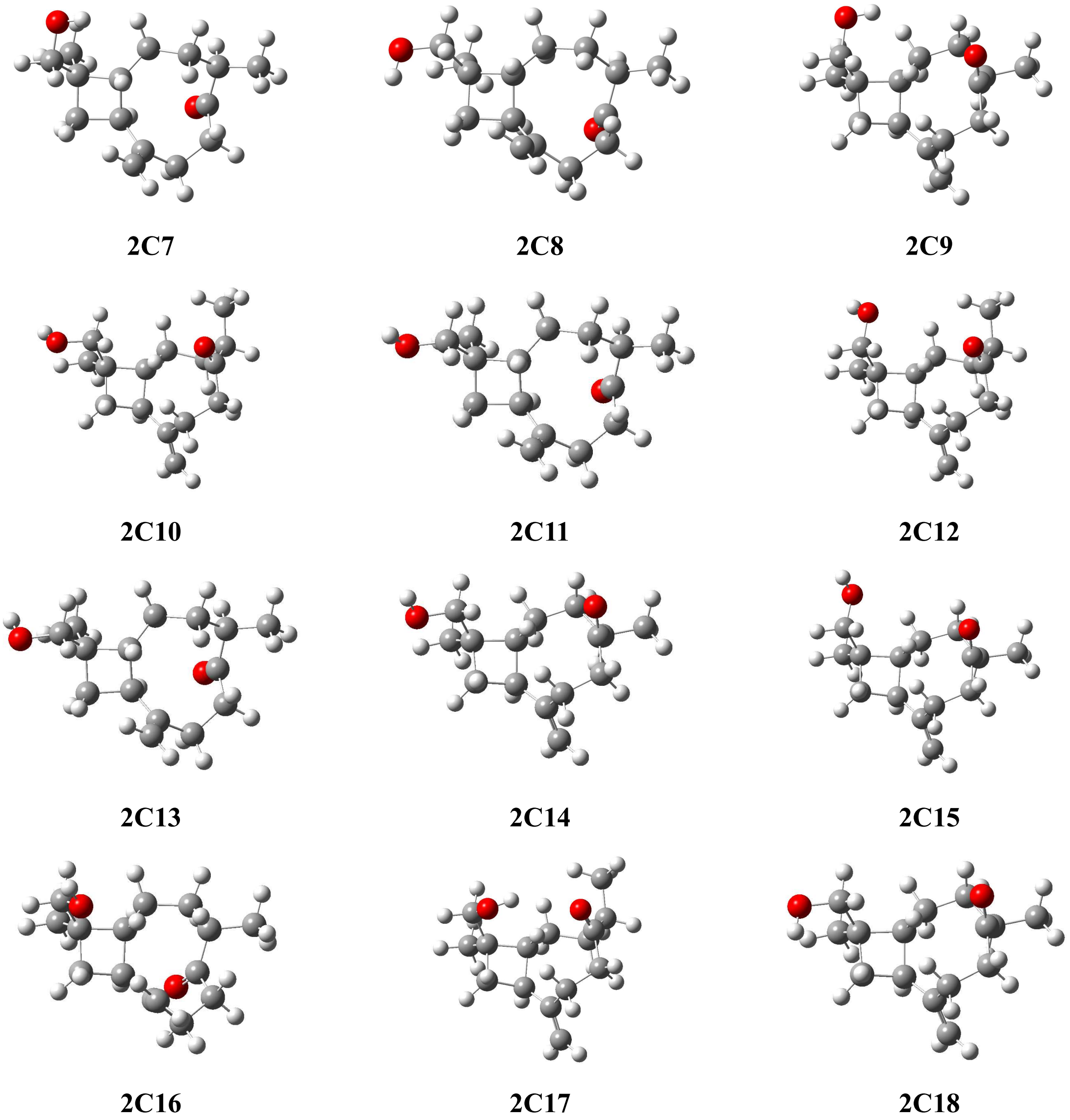

Figure S2. *ω*B97XD/DGDZVP optimized 18 conformers of (1*R*, 4*R*, 9*S*, 11*R*)-**2**.

Table S2. Energy analysis for the conformers of (1*R*, 4*R*, 9*S*, 11*R*)-**2**.

Conf.	MMFF energy	B3LYP/6-31G(d) Gibbs free energy (298.15 K)			ωB97XD/DGDZVP Gibbs free energy (298.15 K)		
	ΔE (Kcal/mol)	G (Hartree)	ΔG (Kcal/mol)	Boltzmann distribution	G (Hartree)	ΔG (Kcal/mol)	Boltzmann distribution
**2C1**	0.00	-736.120507	0.0000	0.068	-735.993915	0.0000	0.136
**2C2**	0.20	-736.119977	0.3330	0.039	-735.99335	0.3550	0.075
**2C3**	0.45	-736.11906	0.9080	0.015	-735.992955	0.6020	0.049
**2C4**	0.64	-736.119804	0.4410	0.032	-735.993786	0.0810	0.119

**2C5**	0.72	-736.119923	0.3660	0.036	-735.991848	1.2970	0.015
**2C6**	0.77	-736.120159	0.2180	0.047	-735.992384	0.9610	0.027
**2C7**	1.04	-736.119934	0.3600	0.037	-735.992441	0.9250	0.028
**2C8**	1.14	-736.120013	0.3100	0.04	-735.992683	0.7730	0.037
**2C9**	1.16	-736.120064	0.2780	0.042	-735.992956	0.6020	0.049
**2C10**	1.31	-736.118916	0.9980	0.013	-735.993362	0.3470	0.076
**2C11**	1.34	-736.118922	0.9950	0.013	-735.991792	1.3320	0.014
**2C12**	1.35	-736.119393	0.6990	0.021	-735.993884	0.0190	0.131
**2C13**	1.42	-736.119274	0.7740	0.018	-735.992171	1.0940	0.021
**2C14**	1.46	-736.118962	0.9700	0.013	-735.991468	1.5360	0.01
**2C15**	1.50	-736.119222	0.8060	0.017	-735.99156	1.4780	0.011
**2C16**	1.61	-736.120593	-0.0540	0.074	-735.992337	0.9900	0.026
**2C17**	1.65	-736.122288	-1.1180	0.447	-735.994022	-0.0670	0.152
**2C18**	1.73	-736.119673	0.5230	0.028	-735.992251	1.0440	0.023

Text S4. ECD calculation of compound 3a.

ECD calculation of (1*R*, 5*S*, 8*S*, 9*S*, 11*R*)-3a Conformation searches based on molecular mechanics with MMFF94s force field were performed for (1*R*, 5*S*, 8*S*, 9*S*, 11*R*)-**3a** and gave 9 conformers with relative energies within 2 kcal/mol [1]. The selected conformers were optimized using DFT at the B3LYP/6-31G (d) level in a vacuum with the Gaussian 16 program (Table S3) [2]. The B3LYP/6-31G (d)-optimized conformers (Boltzmann distribution ≥ 1%) were then reoptimized at the *ω*B97XD/DGDZVP level in acetonitrile. ECD computations for the *ω*B97XD/DGDZVP-optimized conformers (Figure S3) were conducted at the CAM-B3LYP/DGDZVP level in acetonitrile [3]. According to the Boltzmann distribution theory and the relative Gibbs free energy (ΔG), the ECD spectrum for (1*R*, 5*S*, 8*S*, 9*S*, 11*R*)-**3a** was generated using SpecDis 1.71 with σ = 0.3 eV and a UV shift of +19 nm [4]. The results showed that the theoretically calculated ECD

spectrum of (1*R*, 5*S*, 8*S*, 9*S*, 11*R*)-**3a** was in good agreement with the experimental ECD spectrum of **3a**.

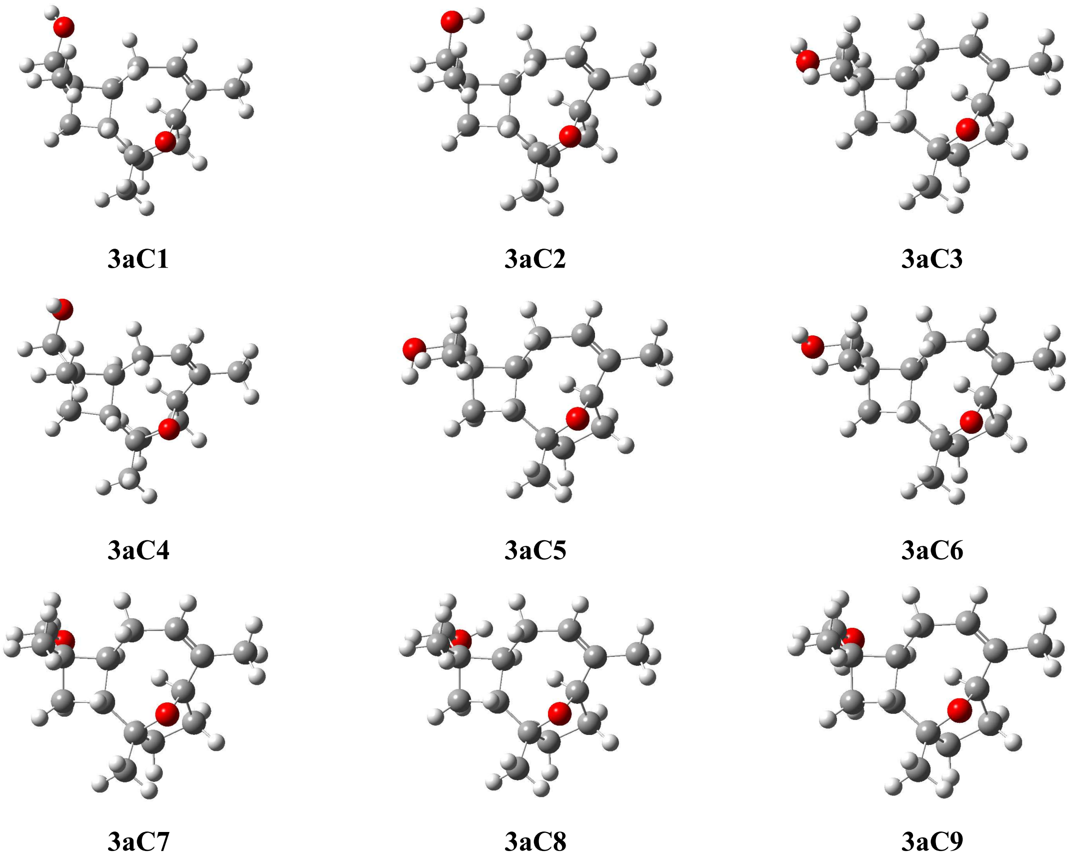

Figure S3. *ω*B97XD/DGDZVP optimized 9 conformers of (1*R*, 5*S*, 8*S*, 9*S*, 11*R*)-**3a**.

Table S3. Energy analysis for the conformers of (1*R*, 5*S*, 8*S*, 9*S*, 11*R*)-**3a**.

Conf.	MMFF energy	B3LYP/6-31G(d) Gibbs free energy (298.15 K)			*ω*B97XD/DGDZVP Gibbs free energy (298.15 K)		
	ΔE (Kcal/mol)	G (Hartree)	ΔG (Kcal/mol)	Boltzmann distribution	G (Hartree)	ΔG (Kcal/mol)	Boltzmann distribution
**3aC1**	0.00	-736.066449	0.0000	0.082	-735.937248	0.0000	0.085
**3aC2**	0.20	-736.066724	-0.1730	0.109	-735.937345	-0.0610	0.094
**3aC3**	0.21	-736.065589	0.5400	0.033	-735.9369	0.2180	0.059
**3aC4**	0.31	-736.06658	-0.0820	0.094	-735.937423	-0.1100	0.102
**3aC5**	0.46	-736.066703	-0.1590	0.107	-735.937879	-0.3960	0.165
**3aC6**	0.46	-736.06581	0.4010	0.042	-735.937225	0.0140	0.083

**3aC7**	0.86	-736.067086	-0.4000	0.161	-735.937281	-0.0210	0.088
**3aC8**	1.65	-736.066985	-0.3360	0.144	-735.937459	-0.1320	0.106
**3aC9**	1.77	-736.067418	-0.6080	0.228	-735.938146	-0.5640	0.219

Text S5. ECD calculation of compound 4a.

ECD calculation of (1*S*, 7*R*, 10*S*)-4a Conformation searches based on molecular mechanics with MMFF94s force field were performed for (1*S*, 7*R*, 10*S*)-**4a** and gave 8 conformers with relative energies within 2 kcal/mol [1]. The selected conformers were optimized using DFT at the B3LYP/6-31G (d) level in a vacuum with the Gaussian 16 program (Table S4) [2]. The B3LYP/6-31G (d)-optimized conformers (Boltzmann distribution ≥ 1%) were then reoptimized at the *ω*B97XD/DGDZVP level in acetonitrile. ECD computations for the *ω*B97XD/DGDZVP-optimized conformers (Figure S4) were conducted at the CAM-B3LYP/DGDZVP level in acetonitrile [3]. According to the Boltzmann distribution theory and the relative Gibbs free energy (ΔG), the ECD spectrum for (1*S*, 7*R*, 10*S*)-**4a** was generated using SpecDis 1.71 with σ = 0.3 eV and a UV shift of +16 nm [4]. The results showed that the theoretically calculated ECD spectrum of (1*S*, 7*R*, 10*S*)-**4a** was in good agreement with the experimental ECD spectrum of **4a**.

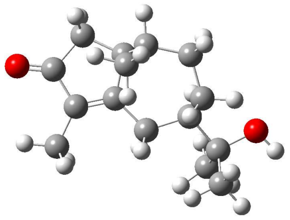

4aC1

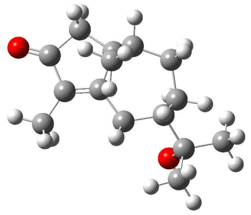

4aC2

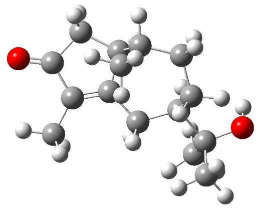

4aC3

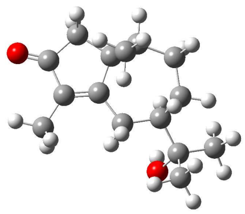

4aC4

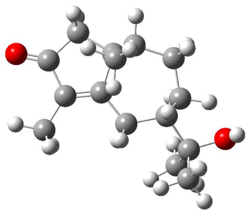

4aC5

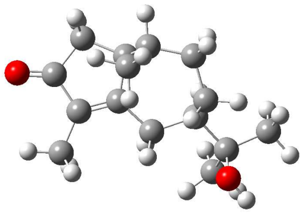

4aC6

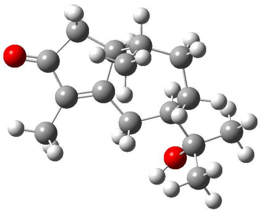

4aC7

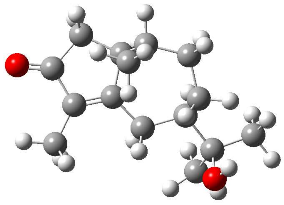

4aC8

Figure S4. *ω*B97XD/DGDZVP optimized 8 conformers of (1*S*, 7*R*, 10*S*)-**4a**.

Table S4. Energy analysis for the conformers of (1*S*, 7*R*, 10*S*)-**4a**.

Conf.	MMFF energy	B3LYP/6-31G(d) Gibbs free energy (298.15 K)			*ω*B97XD/DGDZVP Gibbs free energy (298.15 K)		
	ΔE (Kcal/mol)	G (Hartree)	ΔG (Kcal/mol)	Boltzmann distribution	G (Hartree)	ΔG (Kcal/mol)	Boltzmann distribution
**4aC1**	0	-736.176582	0.0000	0.155	-736.047219	0.0000	0.104
**4aC2**	0.0389	-736.176914	-0.2080	0.221	-736.047702	-0.3030	0.173
**4aC3**	0.1623	-736.176889	-0.1930	0.215	-736.047793	-0.3600	0.191
**4aC4**	0.2522	-736.177044	-0.2900	0.253	-736.048114	-0.5620	0.268
**4aC5**	0.9967	-736.175895	0.4310	0.075	-736.047176	0.0270	0.099
**4aC6**	1.2134	-736.174519	1.2950	0.017	-736.04495	1.4240	0.009
**4aC7**	1.5069	-736.175496	0.6810	0.049	-736.047492	-0.1710	0.139
**4aC8**	1.7133	-736.174371	1.3870	0.015	-736.045443	1.1140	0.016

Text S6. ECD calculation of compound 5a.

ECD calculation of (2*R*, 5*R*, 10*R*)-5a Conformation searches based on molecular mechanics with MMFF94s force field were performed for (2*R*, 5*R*, 10*R*)-**5a** and gave 15 conformers with relative energies within 2 kcal/mol [1]. The selected conformers were optimized using DFT at the B3LYP/6-31G (d) level in a vacuum with the Gaussian 16 program (Table S5) [2]. The B3LYP/6-31G (d)-optimized conformers (Boltzmann distribution ≥ 1%) were then reoptimized at the *ω*B97XD/DGDZVP level in acetonitrile. ECD computations for the *ω*B97XD/DGDZVP-optimized

conformers (Figure S5) were conducted at the CAM-B3LYP/DGDZVP level in acetonitrile [3]. According to the Boltzmann distribution theory and the relative Gibbs free energy (ΔG), the ECD spectrum for (2*R*, 5*R*, 10*R*)-**5a** was generated using SpecDis 1.71 with σ = 0.3 eV and a UV shift of +9 nm [4]. The results showed that the theoretically calculated ECD spectrum of (2*R*, 5*R*, 10*R*)-**5a** was in good agreement with the experimental ECD spectrum of **5a**.

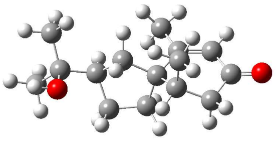

5aC1

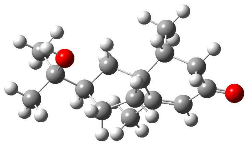

5aC2

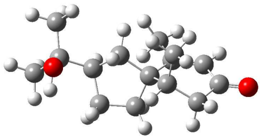

5aC3

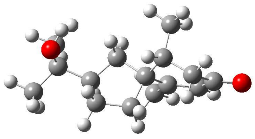

5aC4

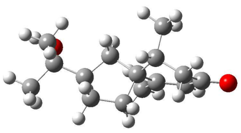

5aC5

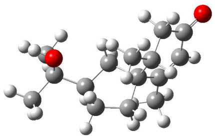

5aC6

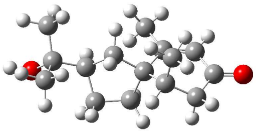

5aC7

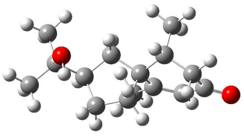

5aC8

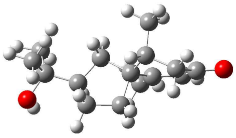

5aC9

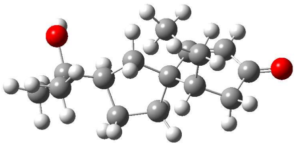

5aC10

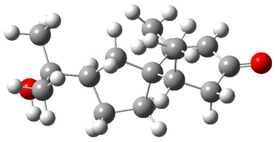

5aC11

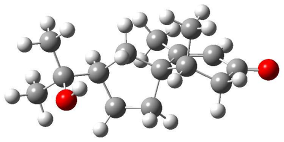

5aC12

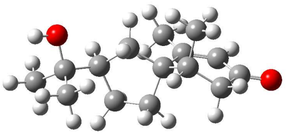

5aC13

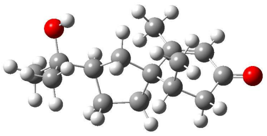

5aC14

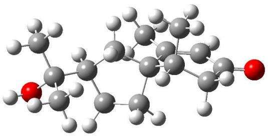

5aC15

Figure S5. *ω*B97XD/DGDZVP optimized 15 conformers of (2*R*, 5*R*, 10*R*)-**5a**.

Table S5. Energy analysis for the conformers of (2*R*, 5*R*, 10*R*)-**5a**.

Conf.	MMFF energy	B3LYP/6-31G(d) Gibbs free energy (298.15 K)			*ω*B97XD/DGDZVP Gibbs free energy (298.15 K)		
	ΔE (Kcal/mol)	G (Hartree)	ΔG (Kcal/mol)	Boltzmann distribution	G (Hartree)	ΔG (Kcal/mol)	Boltzmann distribution

**5aC1**	0	-736.166712	0.0000	0.311	-736.039067	0.0000	0.154
**5aC2**	0.7747	-736.164429	1.4330	0.028	-736.037554	0.9490	0.031
**5aC3**	0.7816	-736.166339	0.2340	0.21	-736.039224	-0.0990	0.182
**5aC4**	1.0086	-736.165815	0.5630	0.12	-736.038724	0.2150	0.107
**5aC5**	1.4622	-736.164902	1.1360	0.046	-736.037867	0.7530	0.043
**5aC6**	1.5295	-736.165654	0.6640	0.102	-736.037916	0.7220	0.046
**5aC7**	1.5659	-736.164635	1.3030	0.034	-736.037907	0.7280	0.045
**5aC8**	1.5704	-736.163803	1.8250	0.014	-736.037796	0.7980	0.04
**5aC9**	1.8365	-736.164467	1.4090	0.029	-736.038403	0.4170	0.076
**5aC10**	1.8523	-736.164605	1.3220	0.033	-736.03853	0.3370	0.087
**5aC11**	1.9233	-736.164398	1.4520	0.027	-736.03815	0.5750	0.058
**5aC12**	1.9348	-736.163543	1.9890	0.011	-736.037611	0.9140	0.033
**5aC13**	2.0118	-736.162627	2.5630	0.004	-736.037236	1.1490	0.022
**5aC14**	2.0247	-736.164395	1.4540	0.027	-736.037984	0.6800	0.049
**5aC15**	2.0311	-736.162542	2.6170	0.004	-736.037384	1.0560	0.026

References

[1] (a) Goto, H.; Osawa, E. Corner flapping: a simple and fast algorithm for exhaustive generation of ring conformations. *J. Am. Chem. Soc.* **1989**, *111*, 8950-8951. (b) Goto, H.; Osawa, E. An efficient algorithm for searching low-energy conformers of cyclic and acyclic molecules. *J. Chem. Soc., Perkin Trans. 2* **1993**, *2*, 187-198.

[2] Frisch, M.J.; Trucks, G.W.; Schlegel, H.B.; Scuseria, G.E.; Robb, M.A.; Cheeseman, J.R.; Scalmani, G.; Barone, V.; Petersson, G.A.; Nakatsuji, H.; Li, X.; Caricato, M.; Marenich, A.V.; Bloino, J.; Janesko, B.G.; Gomperts, R.; Mennucci, B.; Hratchian, H.P.; Ortiz, J.V.; Izmaylov, A.F.; Sonnenberg, J.L.; Williams-Young, D.; Ding, F.; Lipparini, F.; Egidi, F.; Goings, J.; Peng, B.; Petrone, A.; Henderson, T.; Ranasinghe, D.; Zakrzewski, V.G.; Gao, J.; Rega, N.; Zheng, G.; Liang, W.; Hada, M.; Ehara, M.; Toyota, K.; Fukuda, R.; Hasegawa, J.; Ishida, M.; Nakajima, T.; Honda, Y.; Kitao, O.; Nakai, H.; Vreven, T.; Throssell, K.; Montgomery, J.A.Jr.; Peralta, J.E.; Ogliaro, F.; Bearpark, M.J.; Heyd, J.J.; Brothers, E.N.; Kudin, K.N.; Staroverov, V.N.; Keith, T.A.; Kobayashi, R.; Normand, J.; Raghavachari, K.; Rendell, A.P.; Burant, J.C.; Iyengar, S.S.; Tomasi, J.; Cossi, M.; Millam, J.M.; Klene, M.; Adamo, C.; Cammi, R.; Ochterski, J.W.; Martin, R.L.; Morokuma, K.; Farkas, O.; Foresman, J. B.;

Fox, D. J. Gaussian 16, Revision B.01, Gaussian, Inc., Wallingford CT, **2016**.

[3] Liu, Y.; Liu, F.; Qiao M.M.; Guo, L.; Chen, M.H.; Peng, C.; Xiong, L. Curcumanes A and B, two bicyclic sesquiterpenoids with significant vasorelaxant activity from *Curcuma longa*. *Org. Lett.* **2019**, *21*, 1197-1201.

[4] Bruhn, T.; Schaumlöffel, A.; Hemberger, Y.; Bringmann, G. Spec Dis, version 1.71, University of Würzburg, Germany, **2017**.

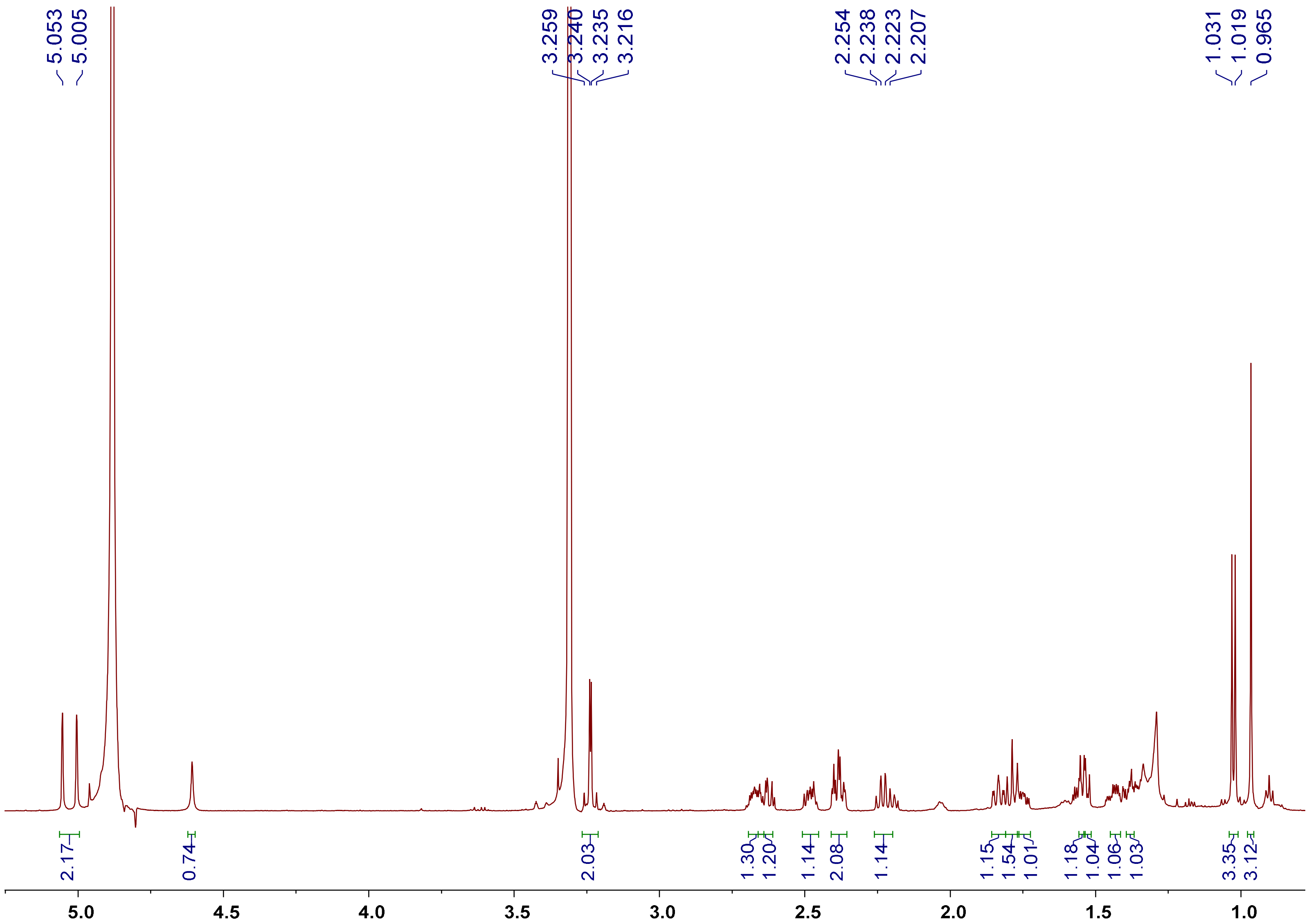

Figure S6. The ^{1}H NMR spectrum of compound **1** in CD_3OD

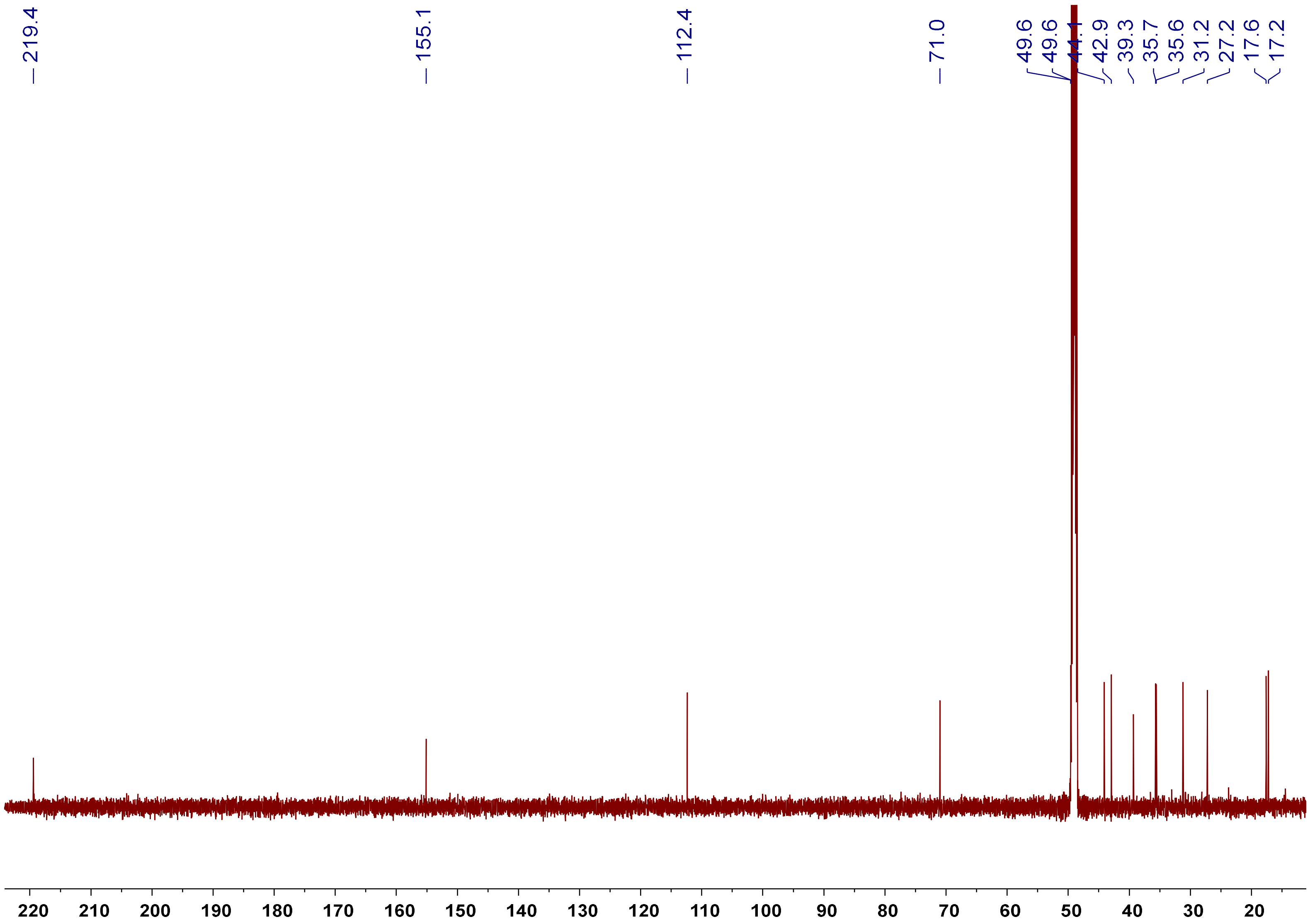

Figure S7. The ^{13}C NMR spectrum of compound **1** in CD_3OD

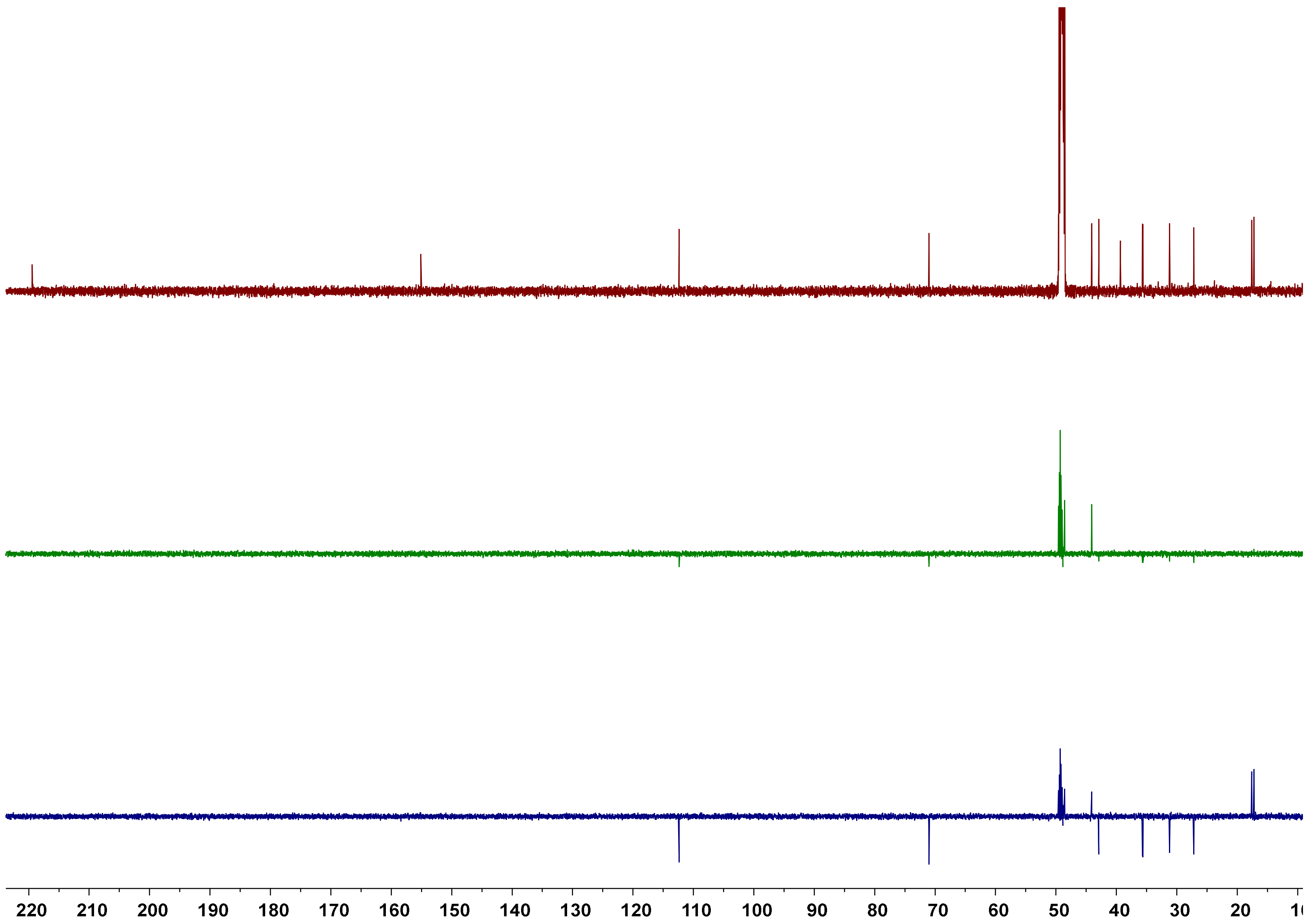

Figure S8. The DEPT spectrum of compound **1** in CD_3OD

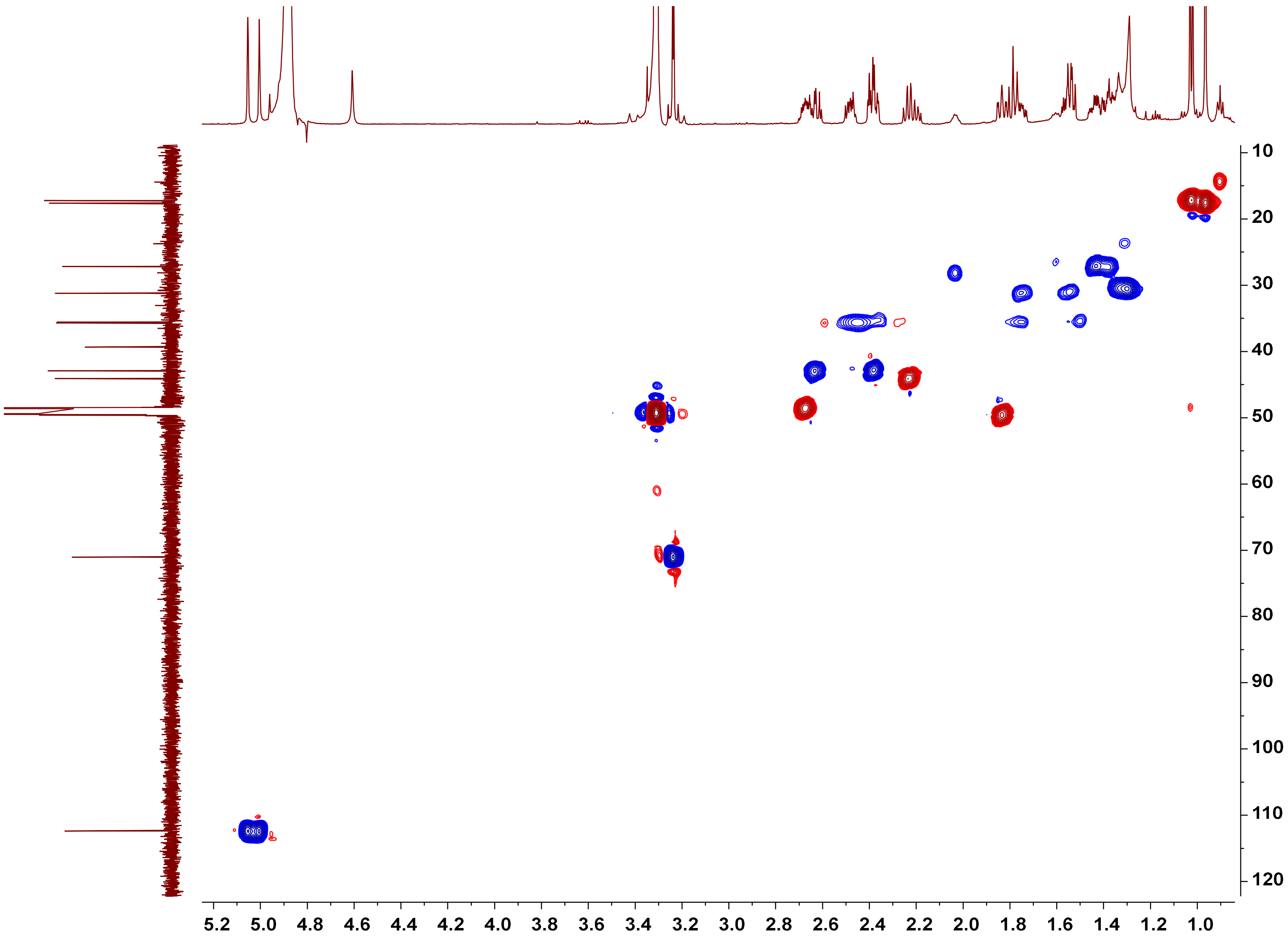

Figure S9. The HSQC spectrum of compound **1** in CD_3OD

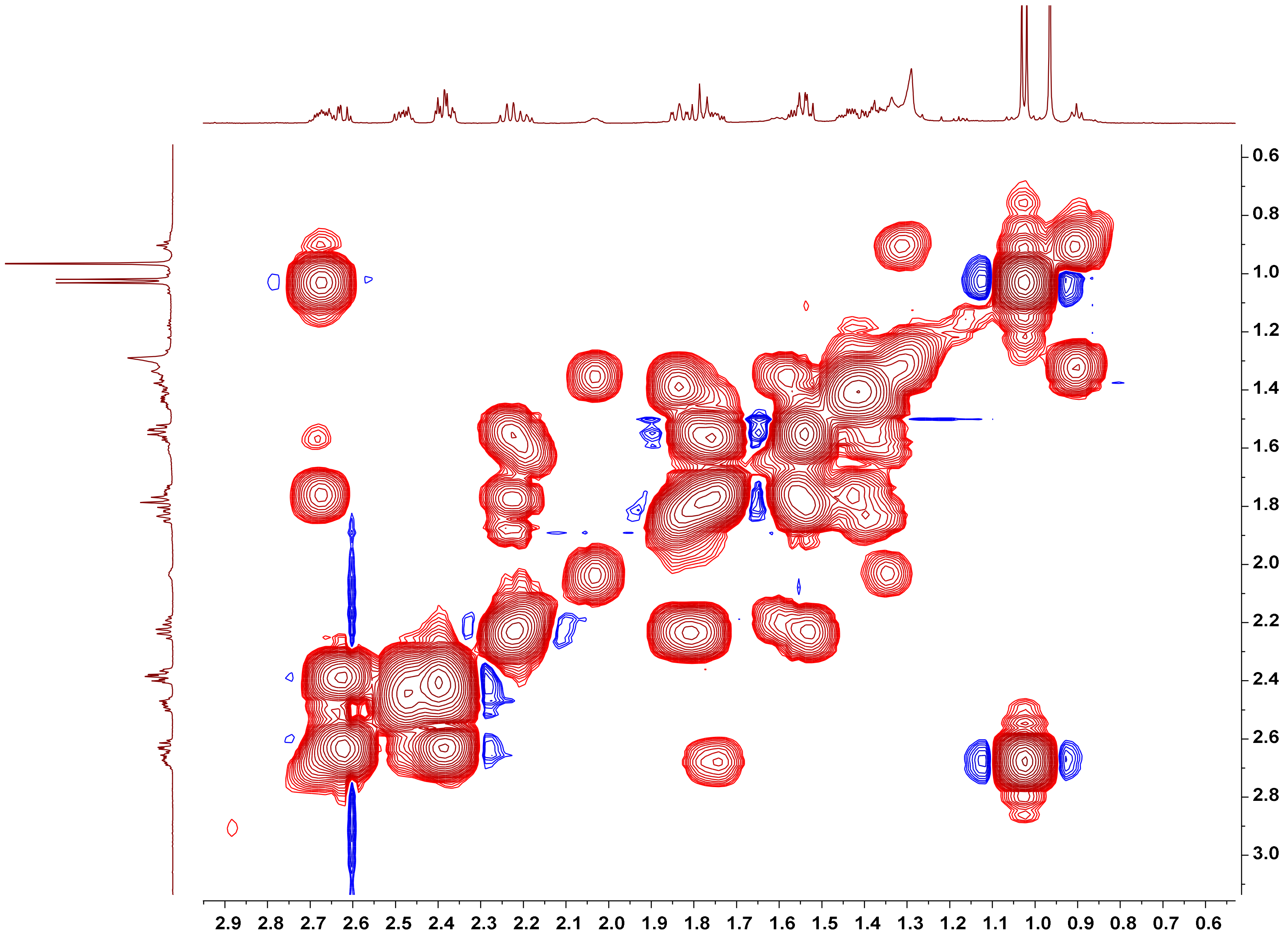

Figure S10. The ^{1}H-^{1}H COSY spectrum of compound **1** in CD_3OD

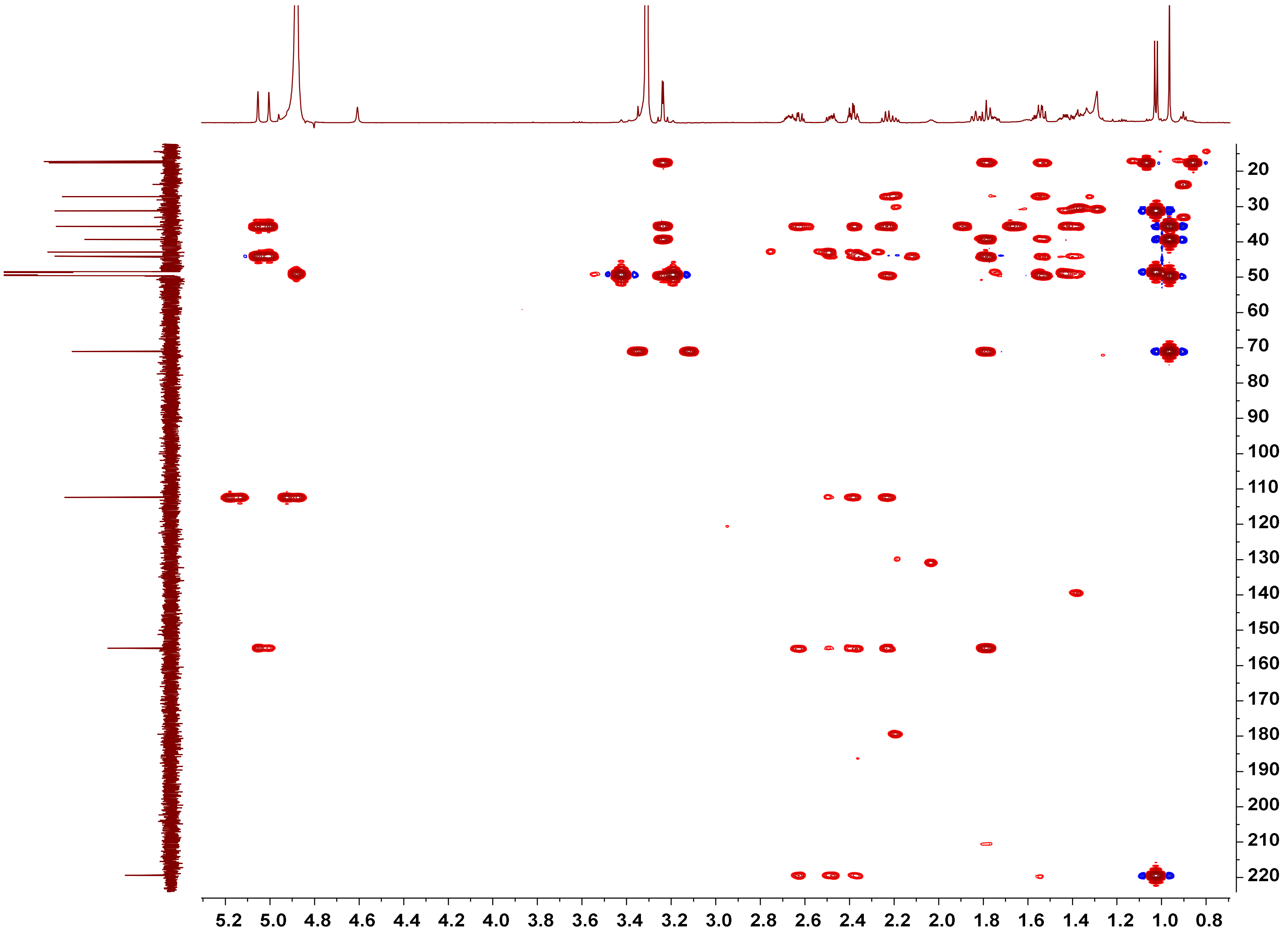

Figure S11. The HMBC spectrum of compound **1** in CD_3OD

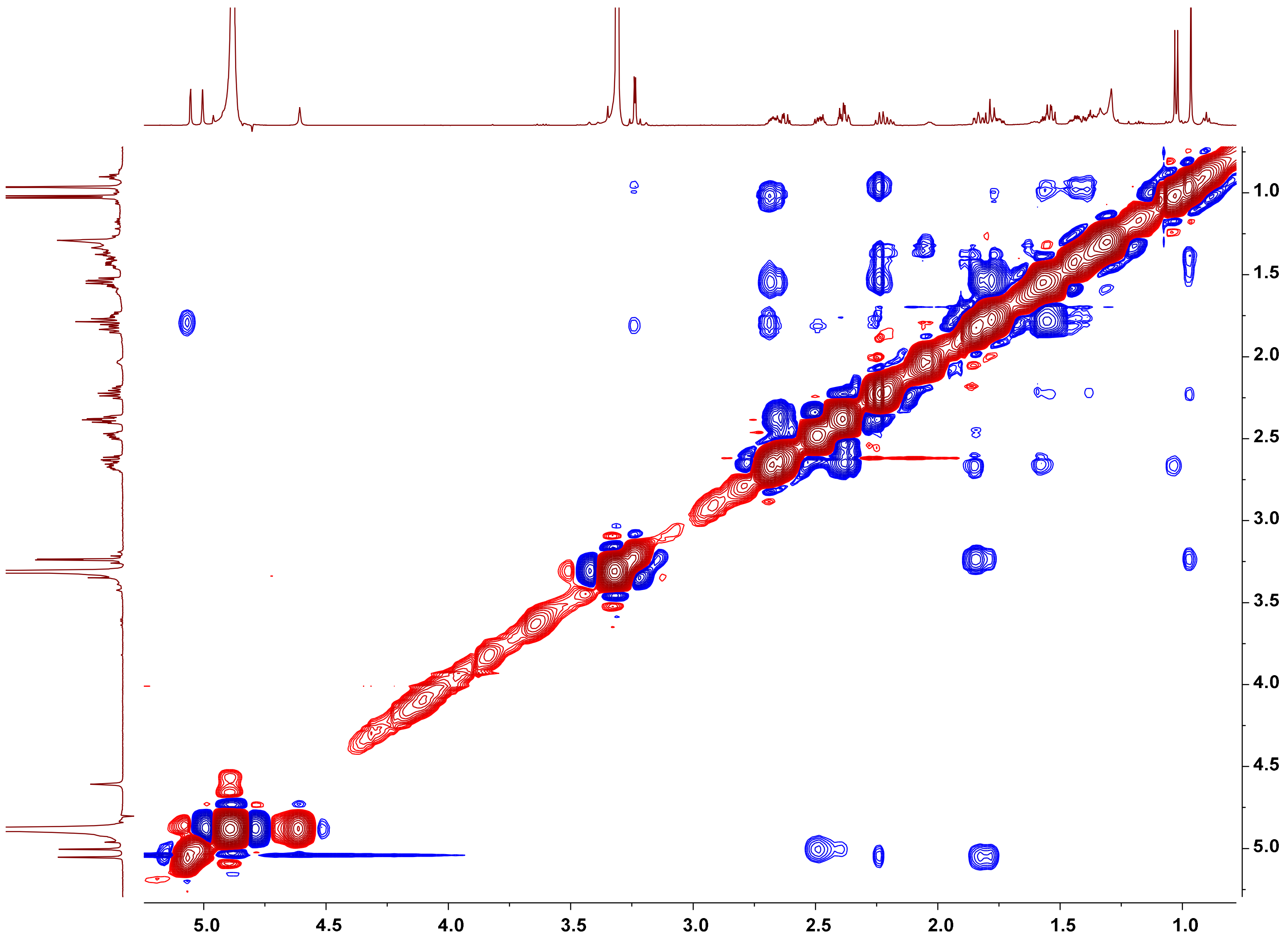

Figure S12. The NOESY spectrum of compound **1** in CD_3OD

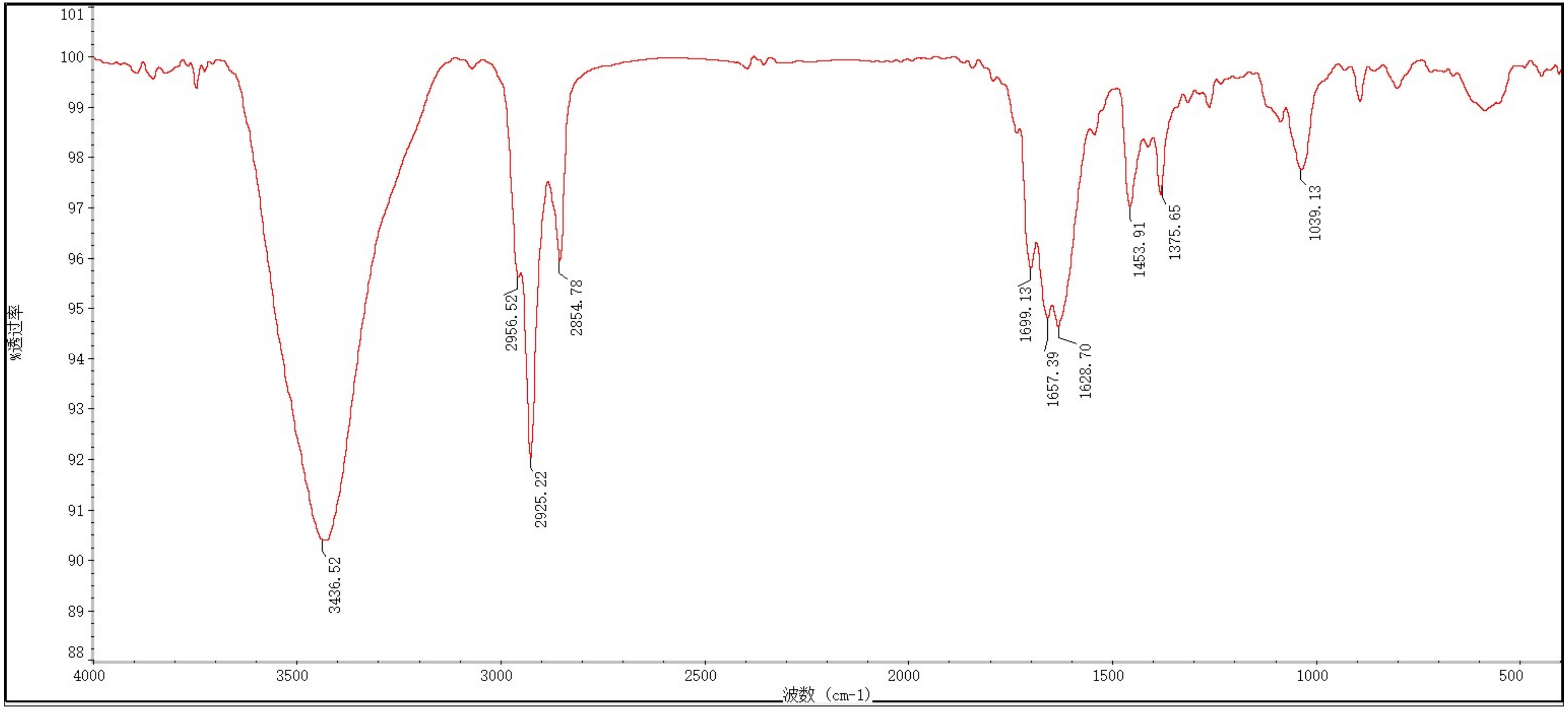

Figure S13. The IR spectrum of compound **1**

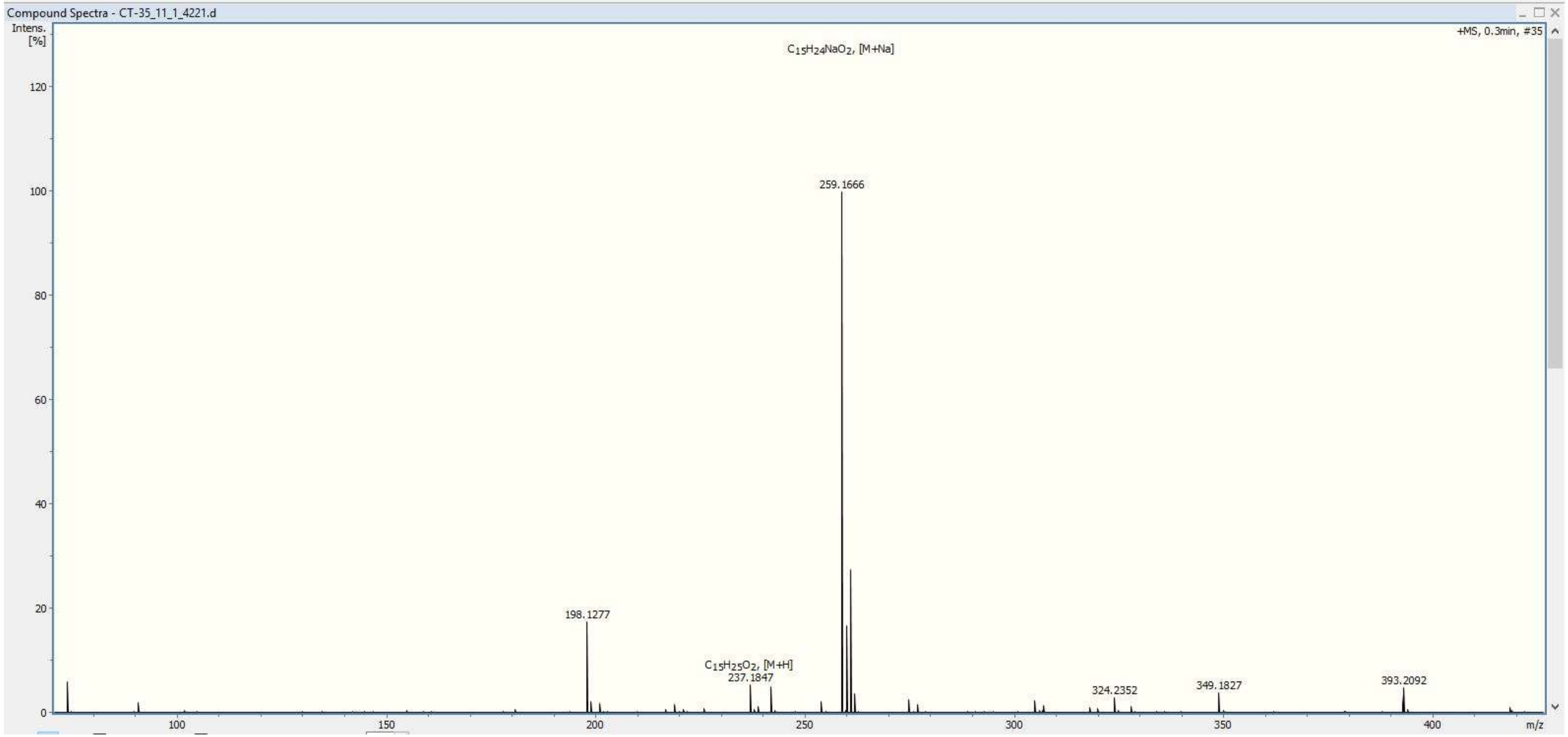

Figure S14. The (+)-HR-ESI-MS spectroscopic data of compound **1**

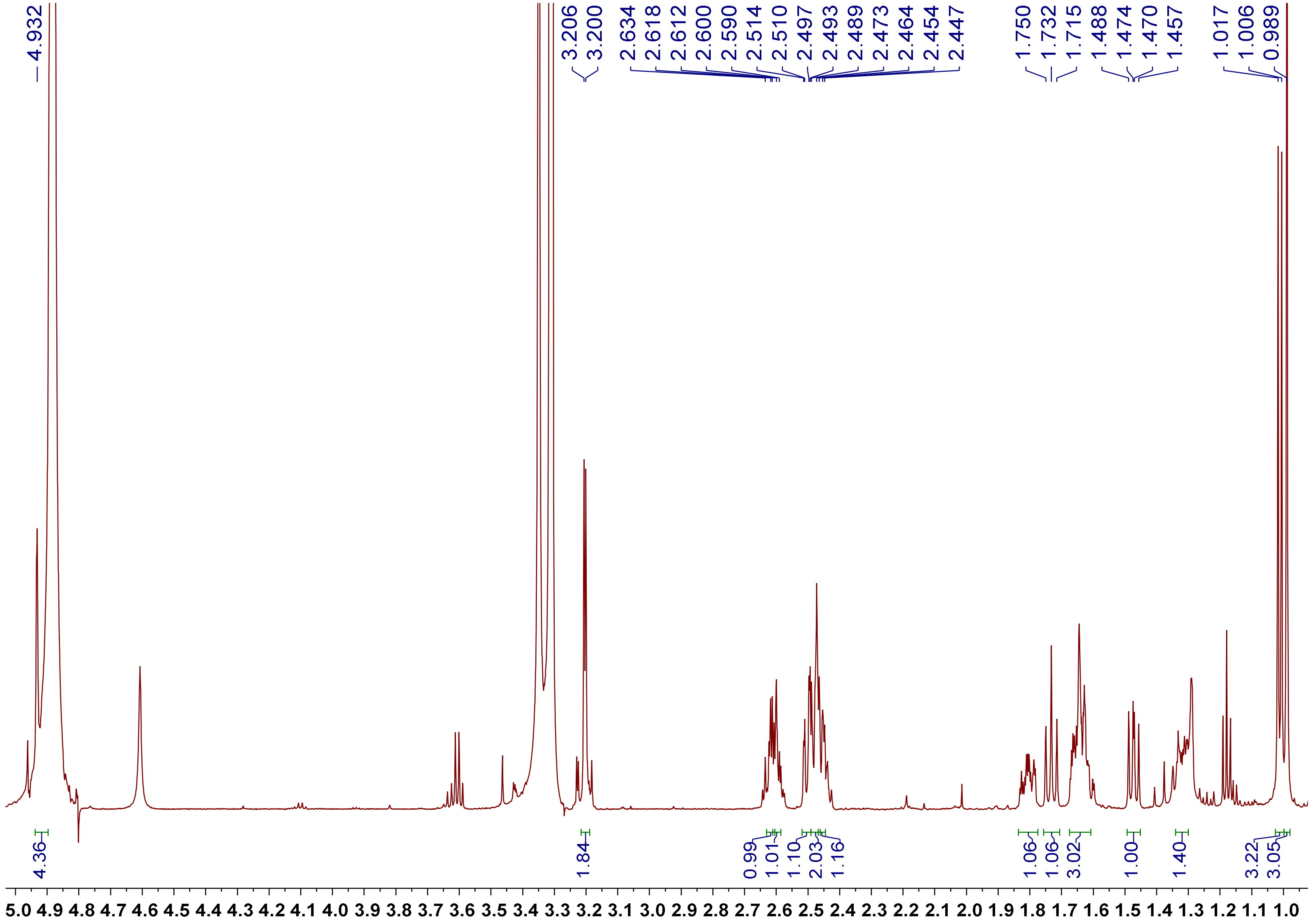

Figure S15. The ^{1}H NMR spectrum of compound **2** in CD_3OD

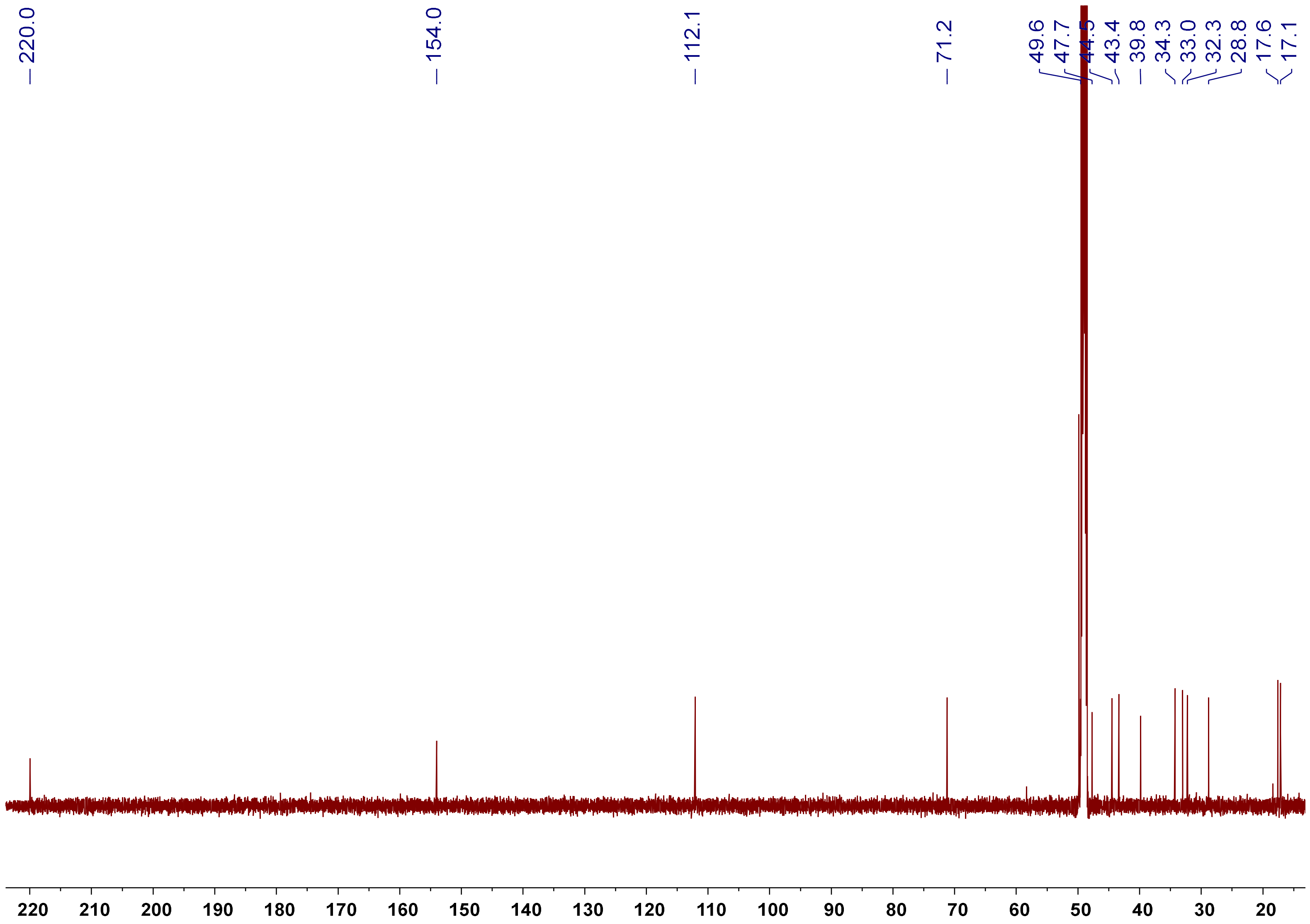

Figure S16. The ^{13}C NMR spectrum of compound **2** in CD_3OD

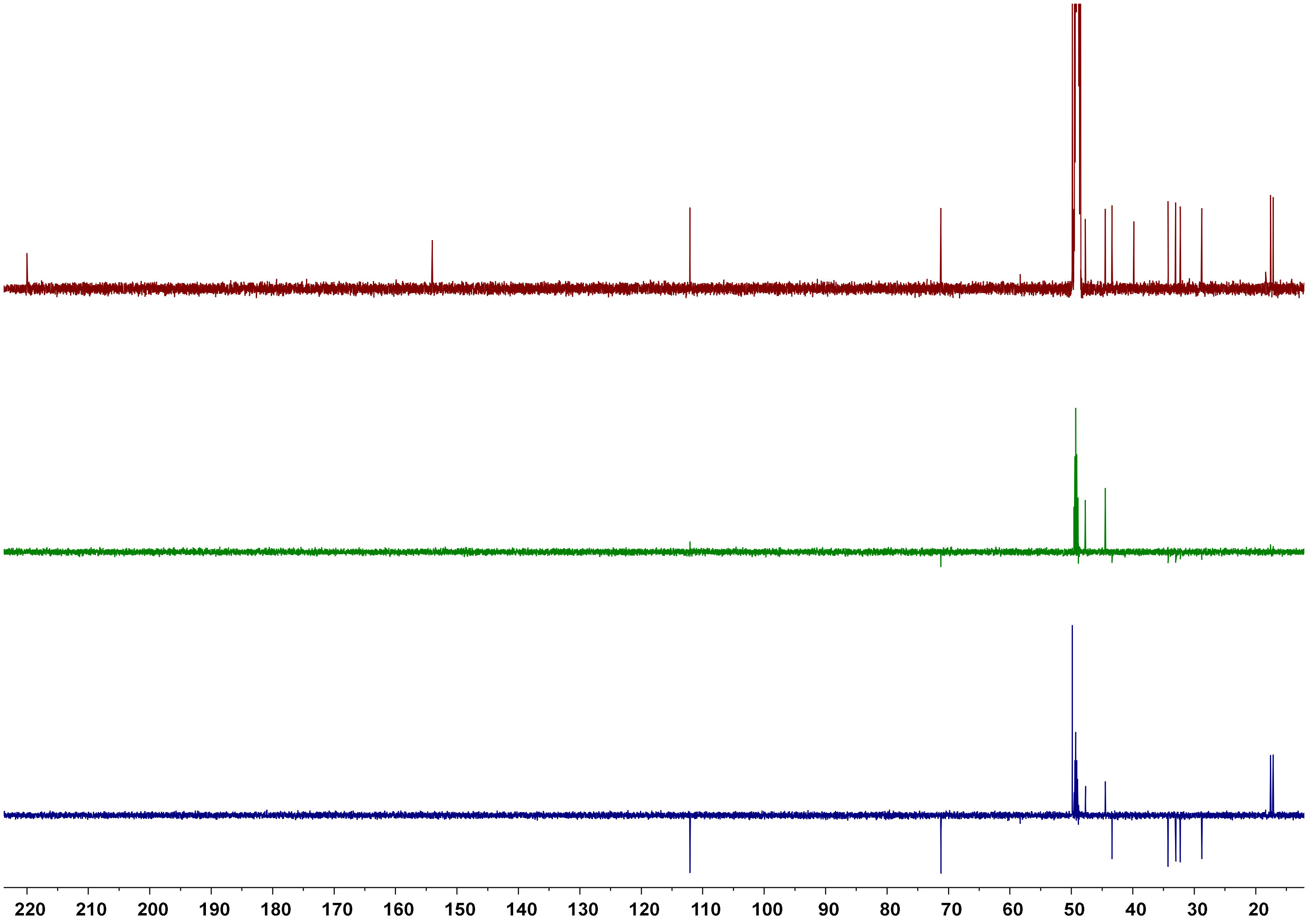

Figure S17. The DEPT spectrum of compound **2** in CD_3OD

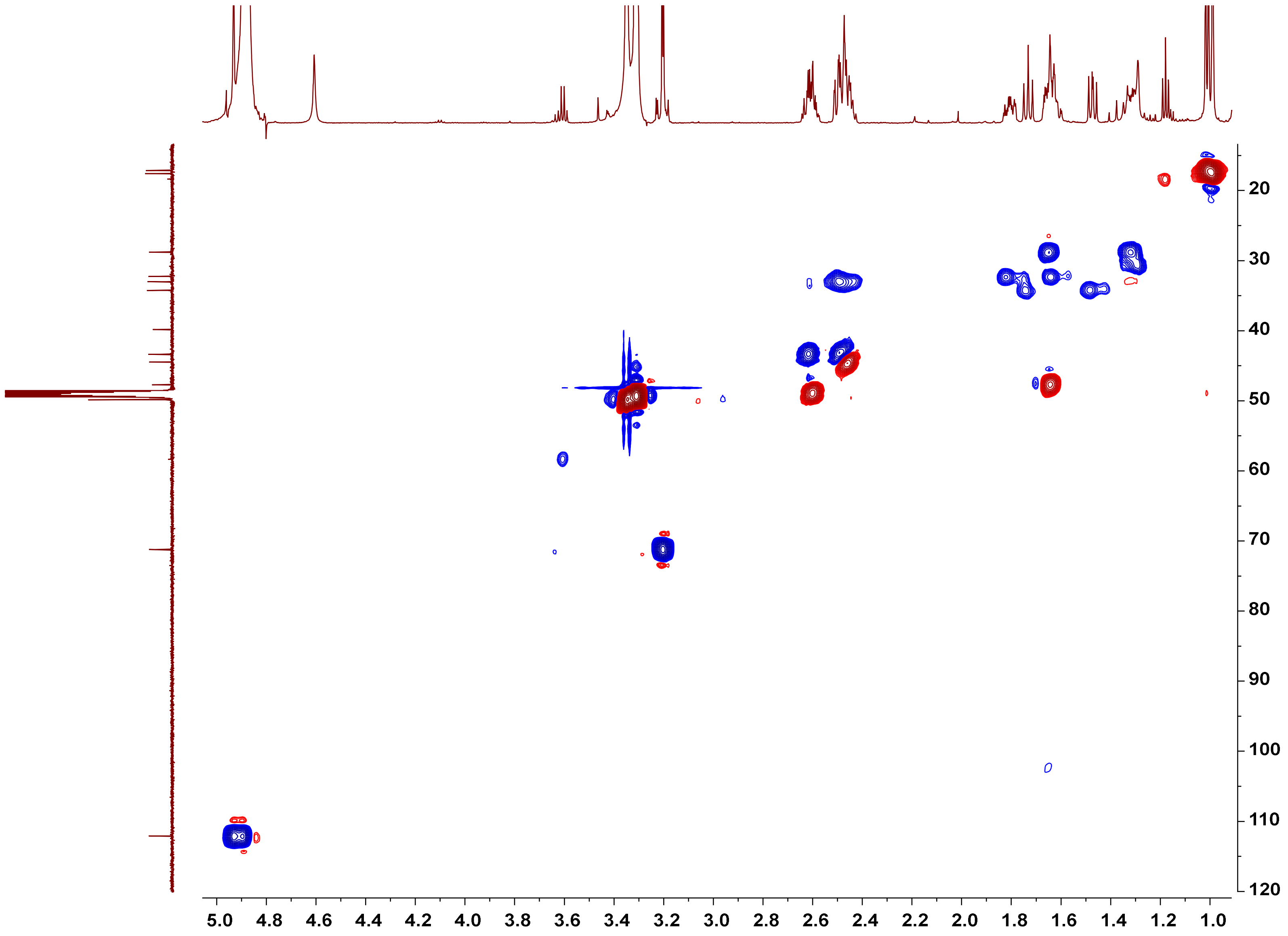

Figure S18. The HSQC spectrum of compound **2** in CD_3OD

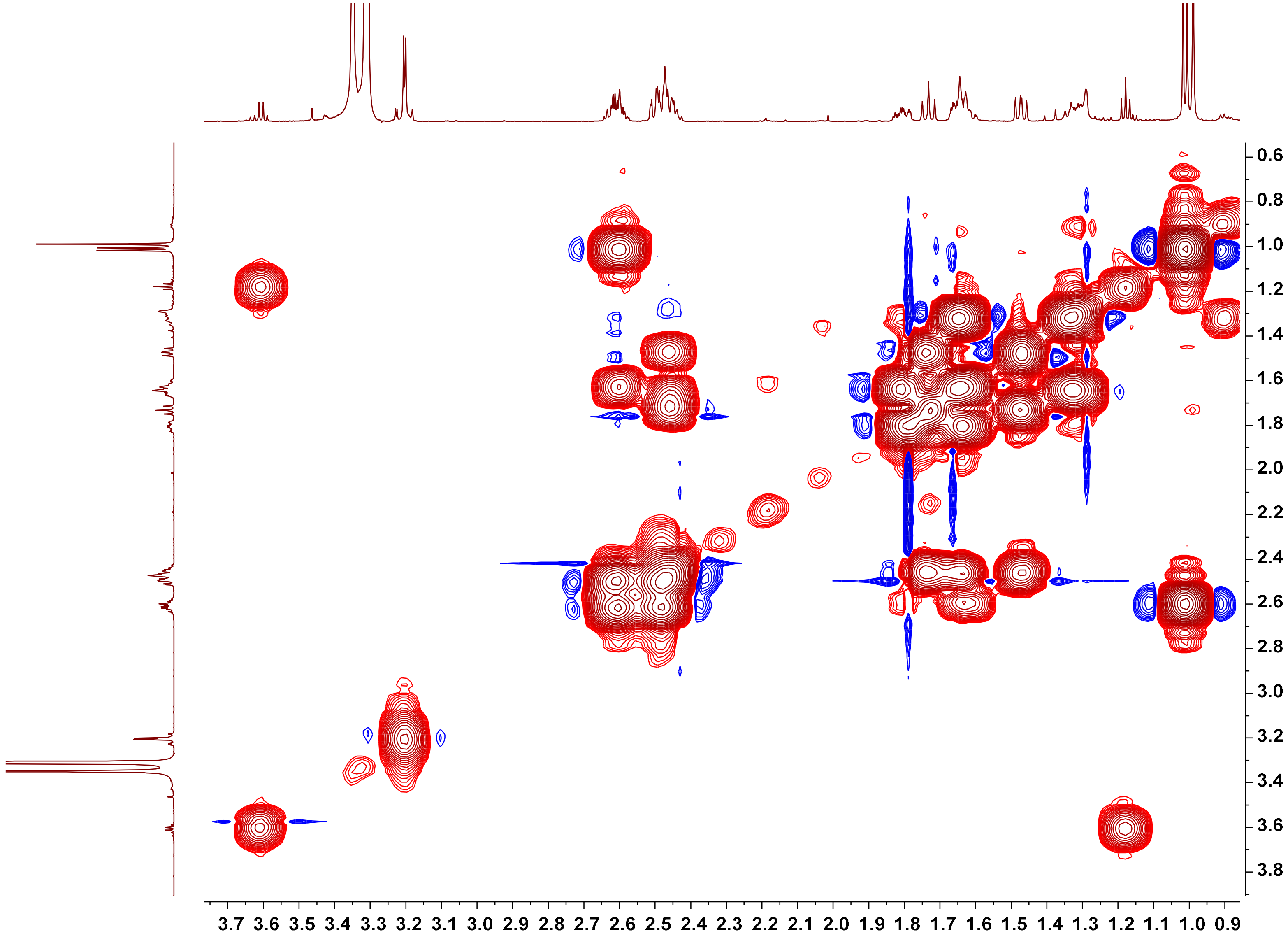

Figure S19. The ^{1}H-^{1}H COSY spectrum of compound **2** in CD_3OD

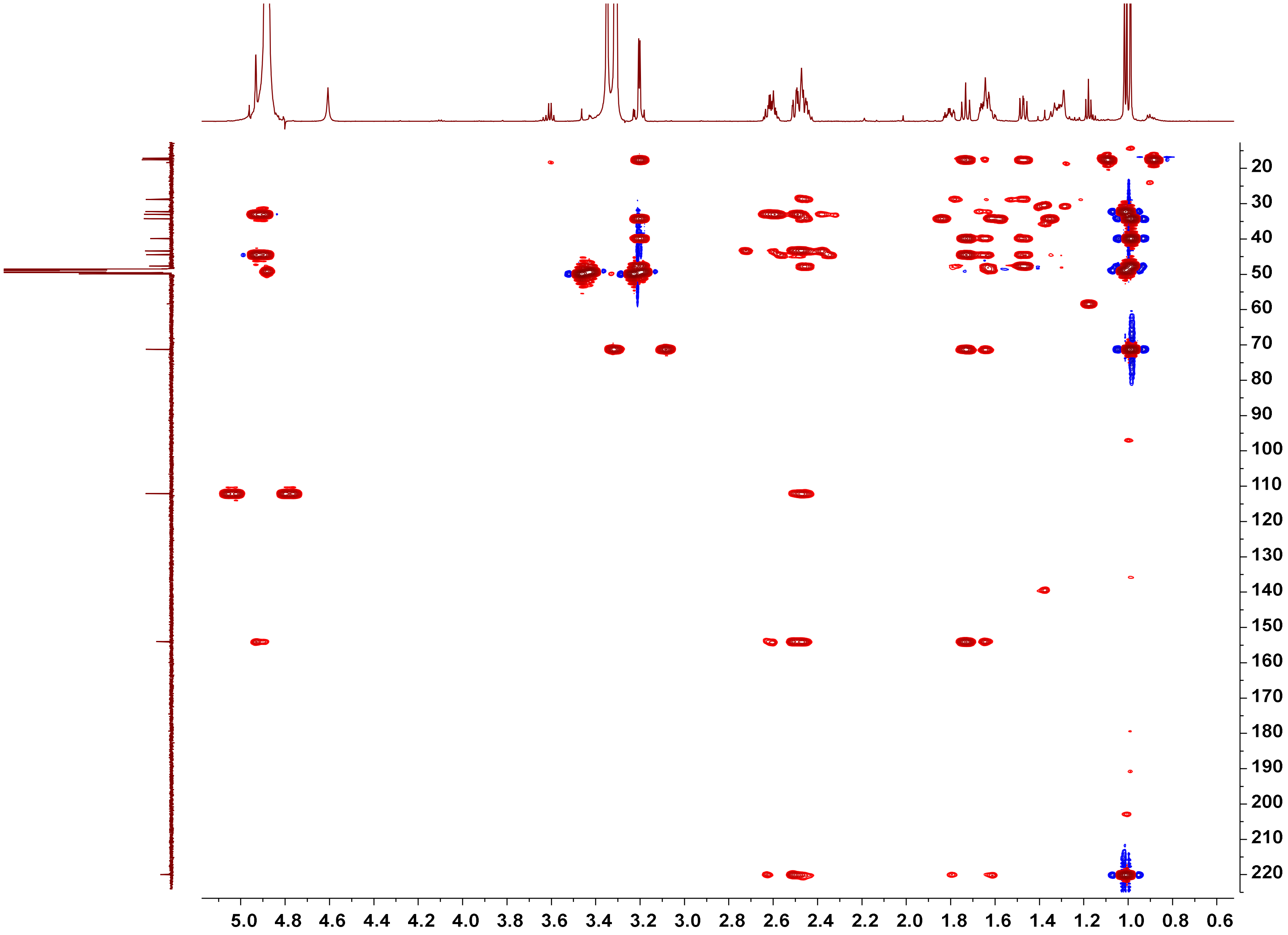

Figure S20. The HMBC spectrum of compound **2** in CD_3OD

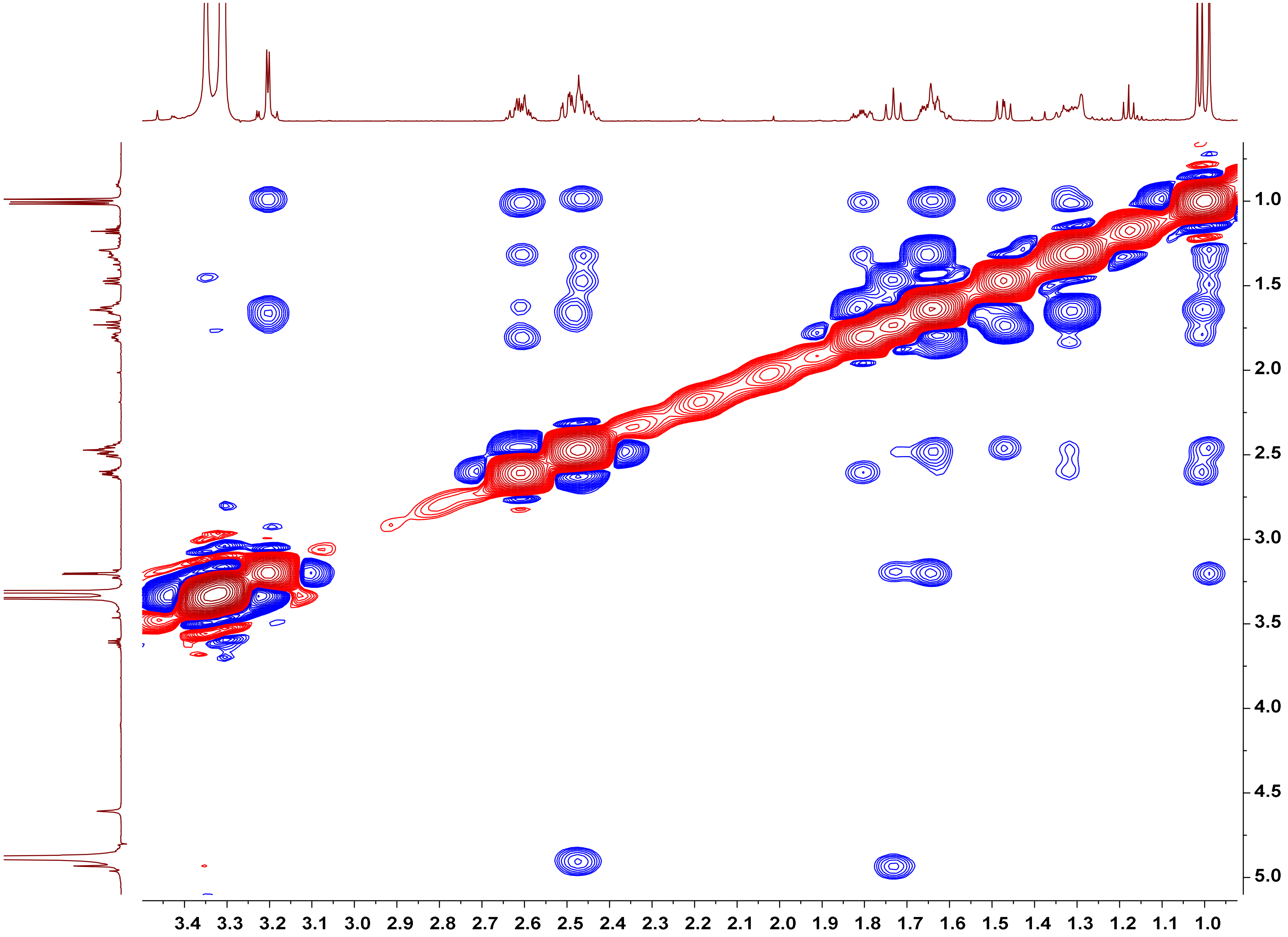

Figure S21. The NOESY spectrum of compound **2** in CD_3OD

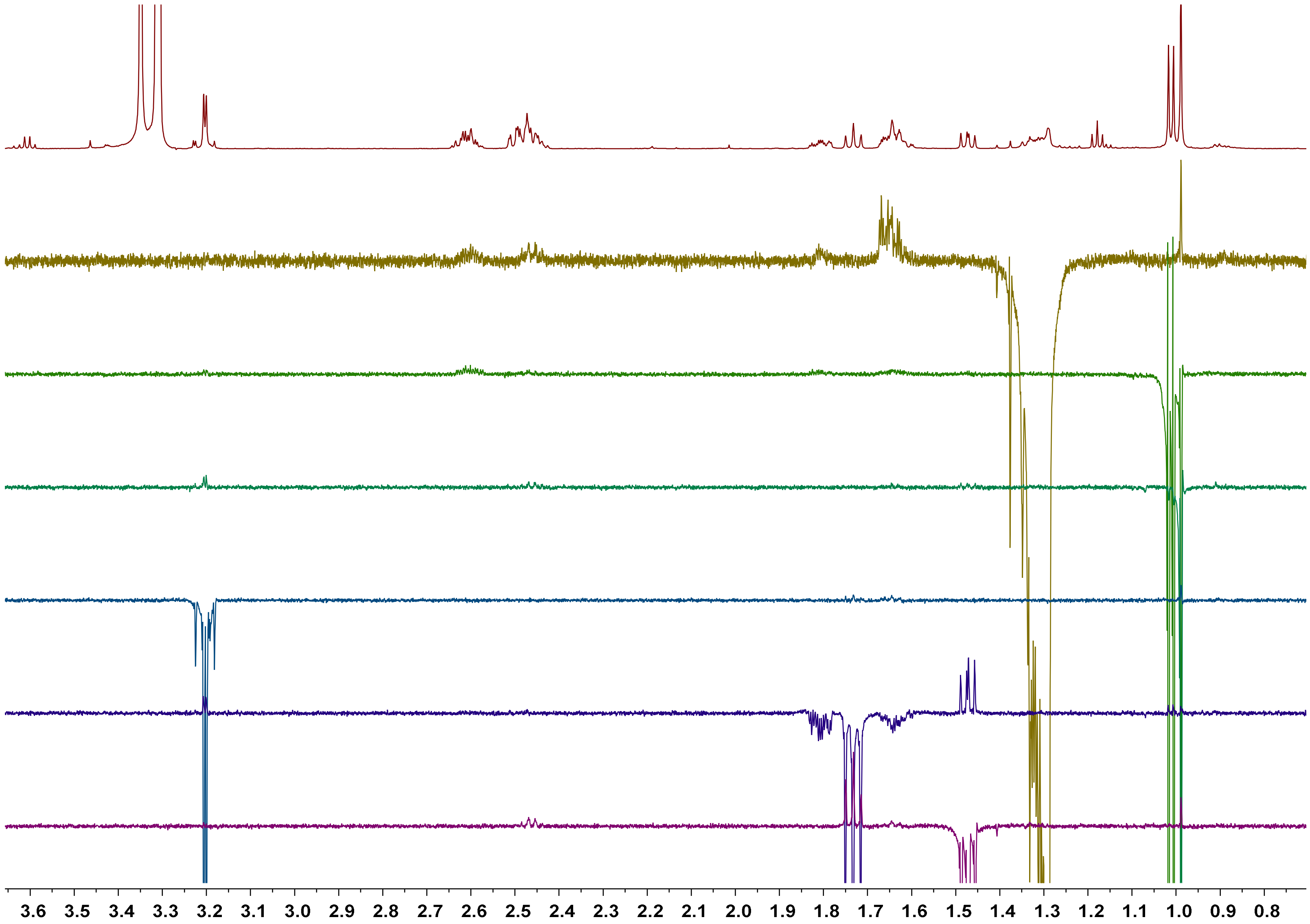

Figure S22. The 1D NOE spectrum of compound **2** in CD_3OD

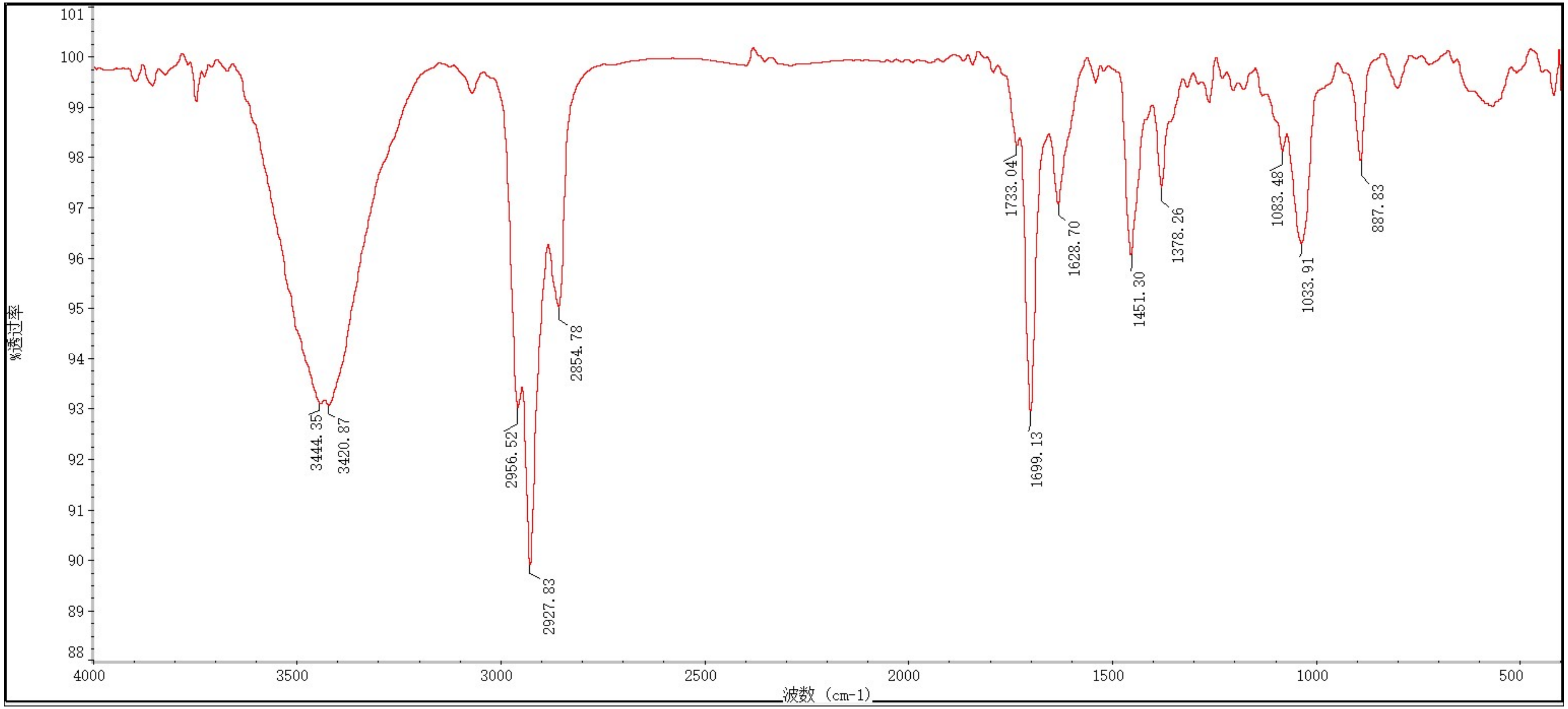

Figure S23. The IR spectrum of compound **2**

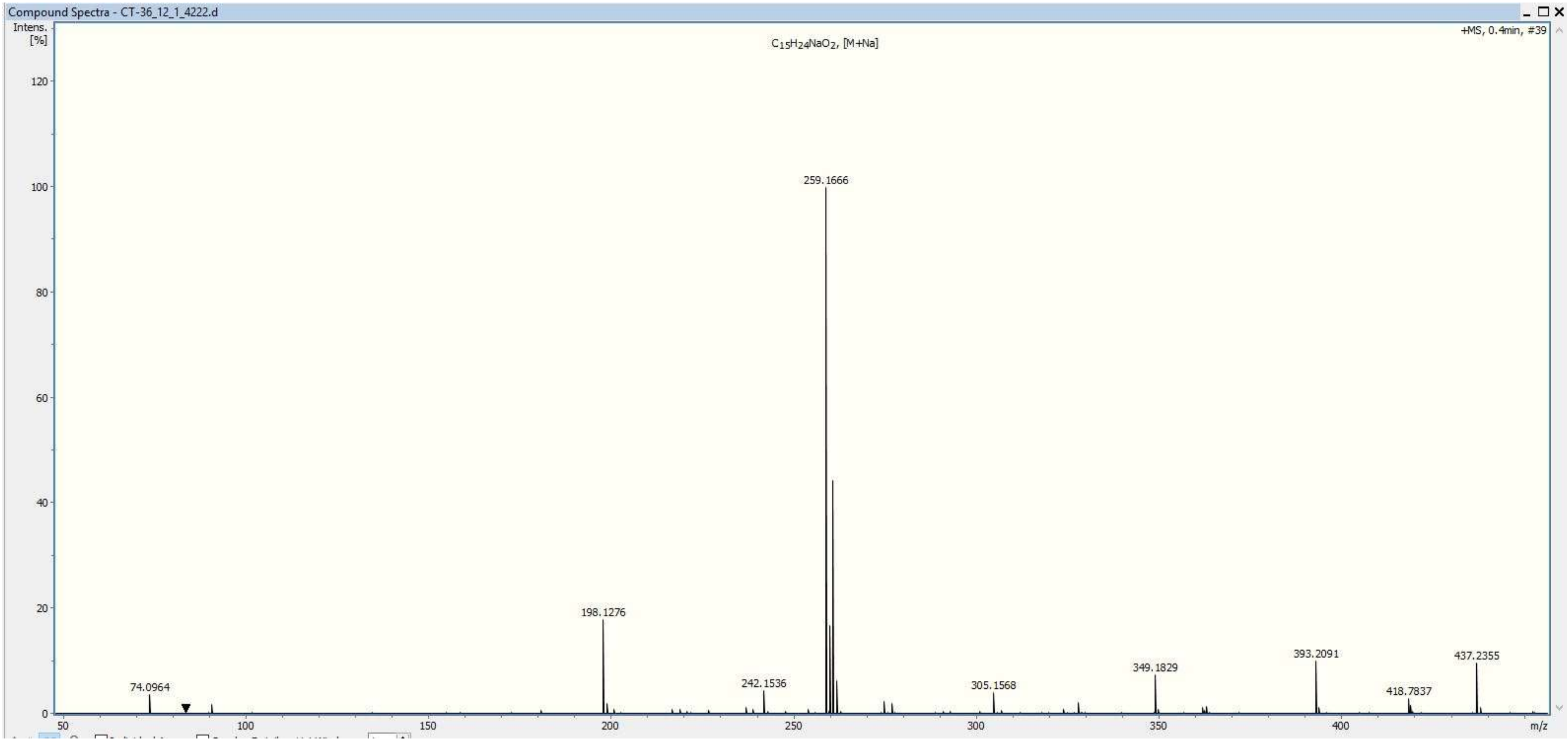

Figure S24. The (+)-HR-ESI-MS spectroscopic data of compound **2**

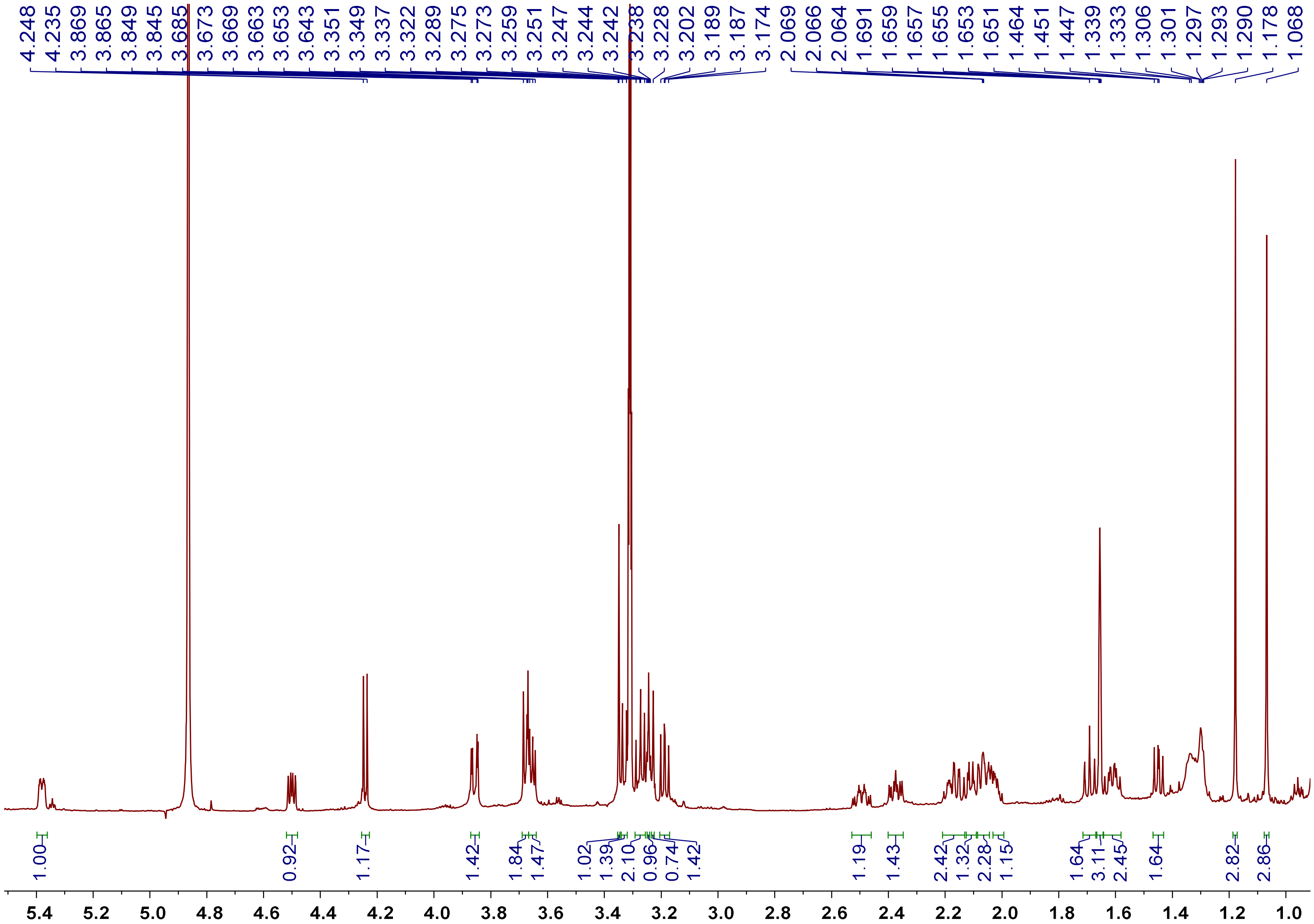

Figure S25. The ^{1}H NMR spectrum of compound **3** in CD_3OD

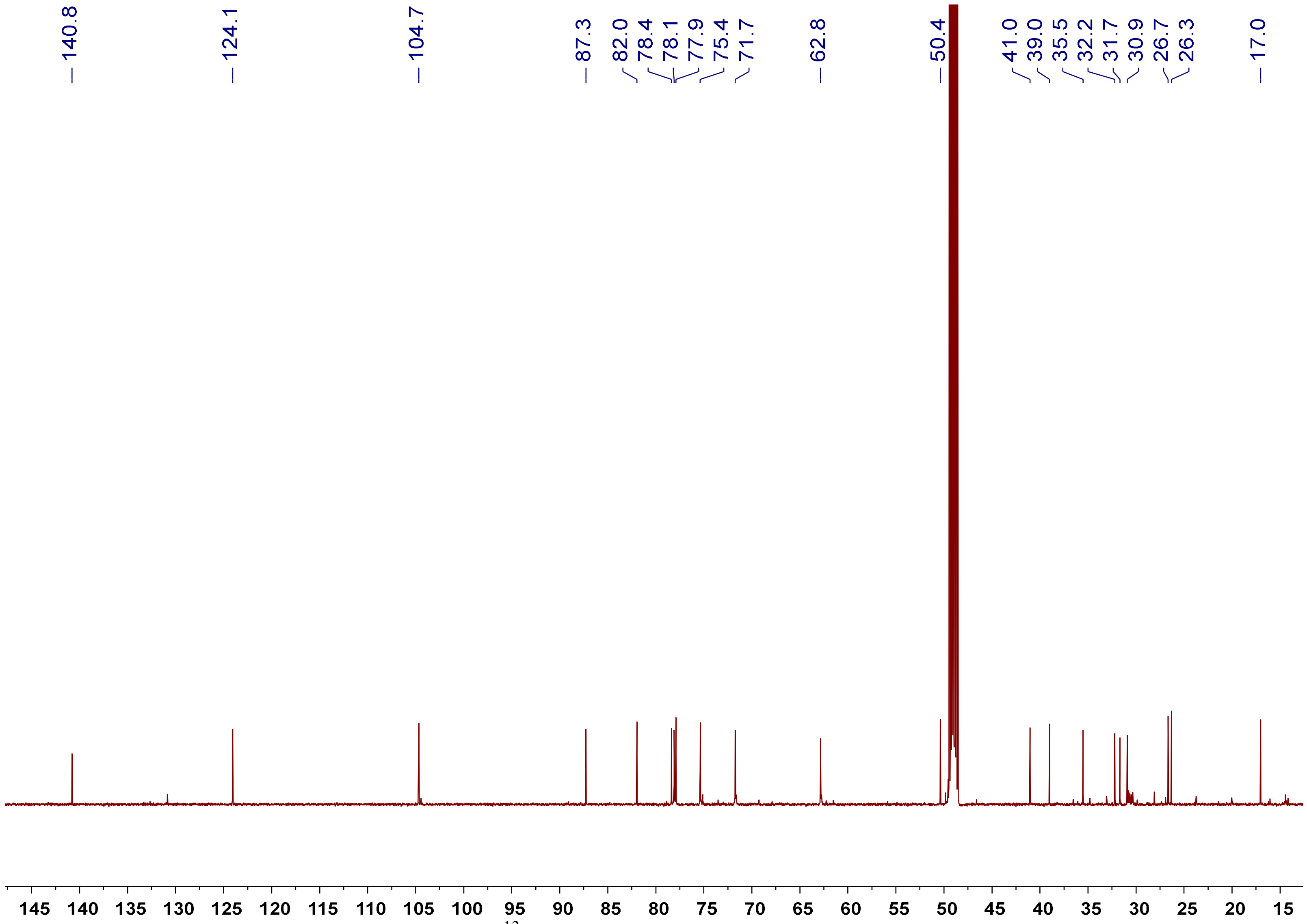

Figure S26. The ^{13}C NMR spectrum of compound **3** in CD_3OD

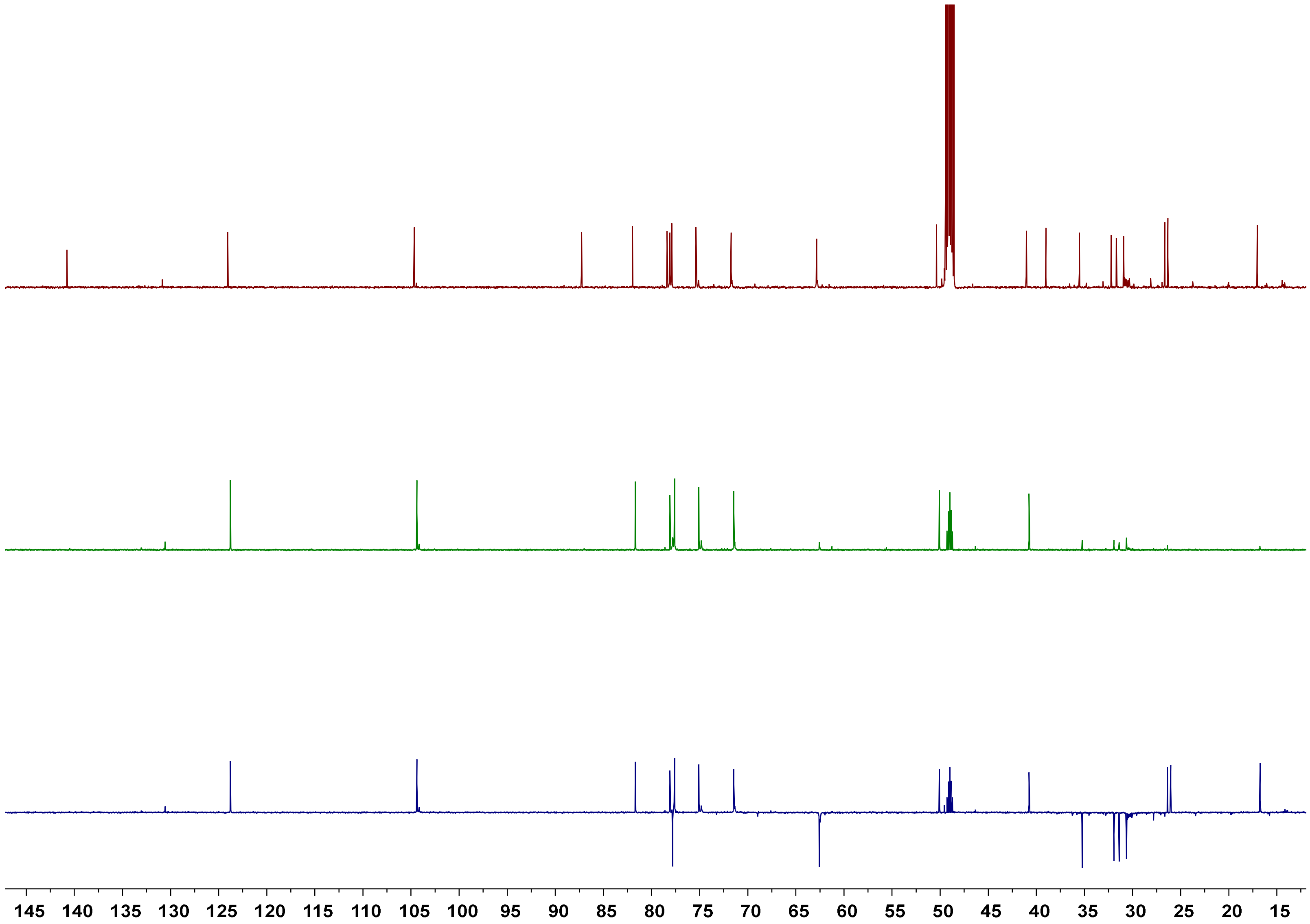

Figure S27. The DEPT spectrum of compound **3** in CD_3OD

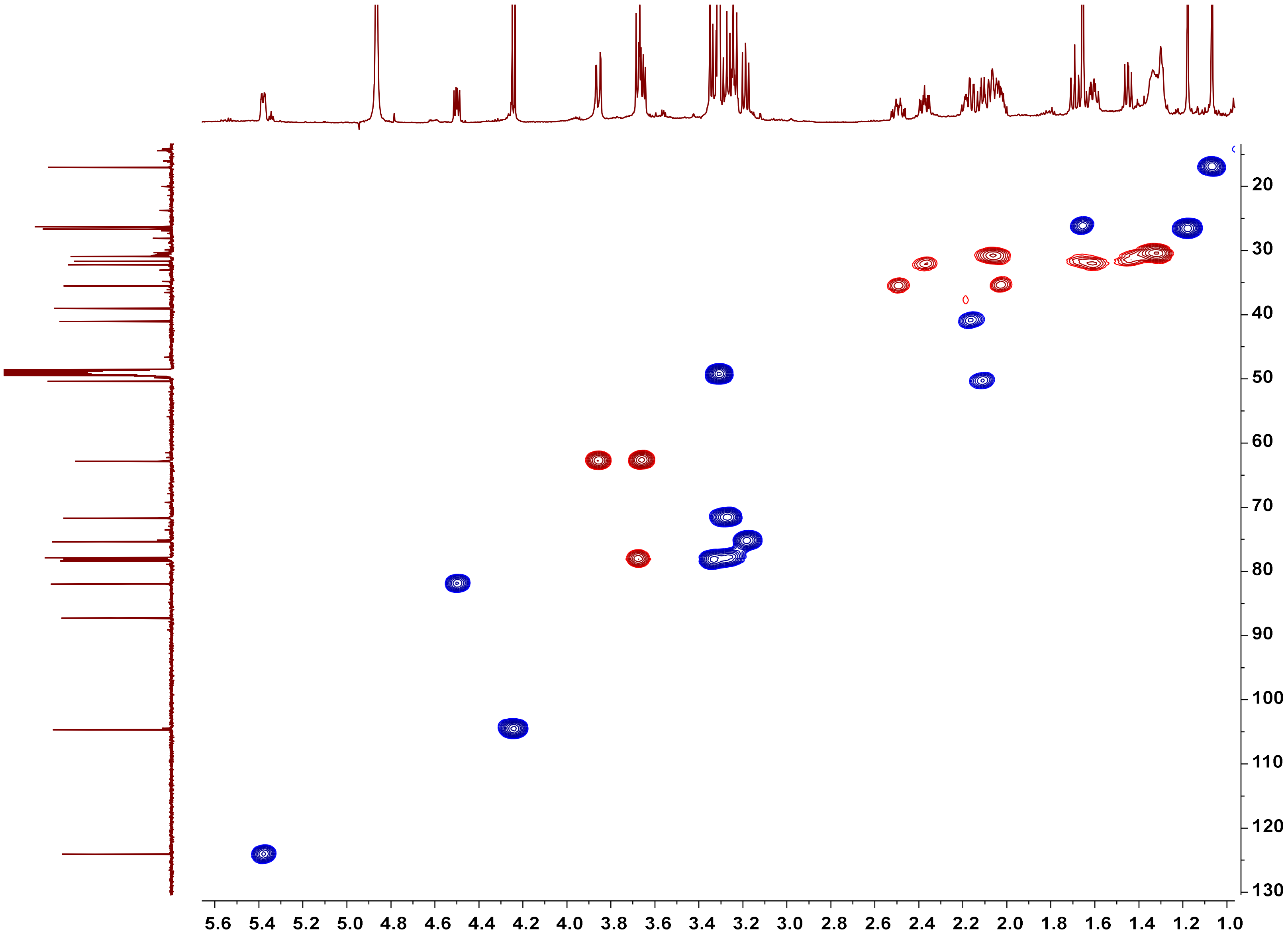

Figure S28. The HSQC spectrum of compound **3** in CD_3OD

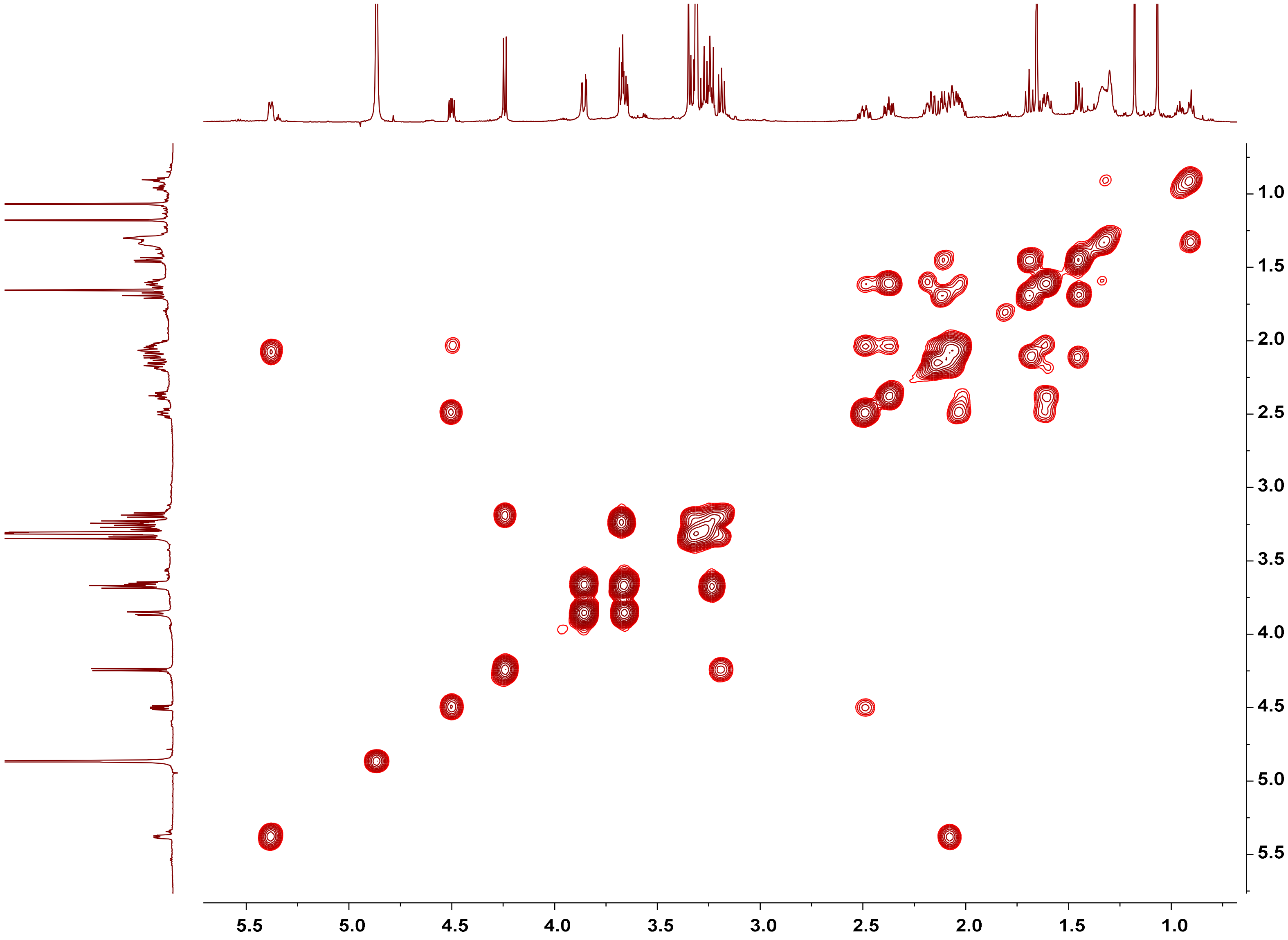

Figure S29. The ^{1}H-^{1}H COSY spectrum of compound **3** in CD_3OD

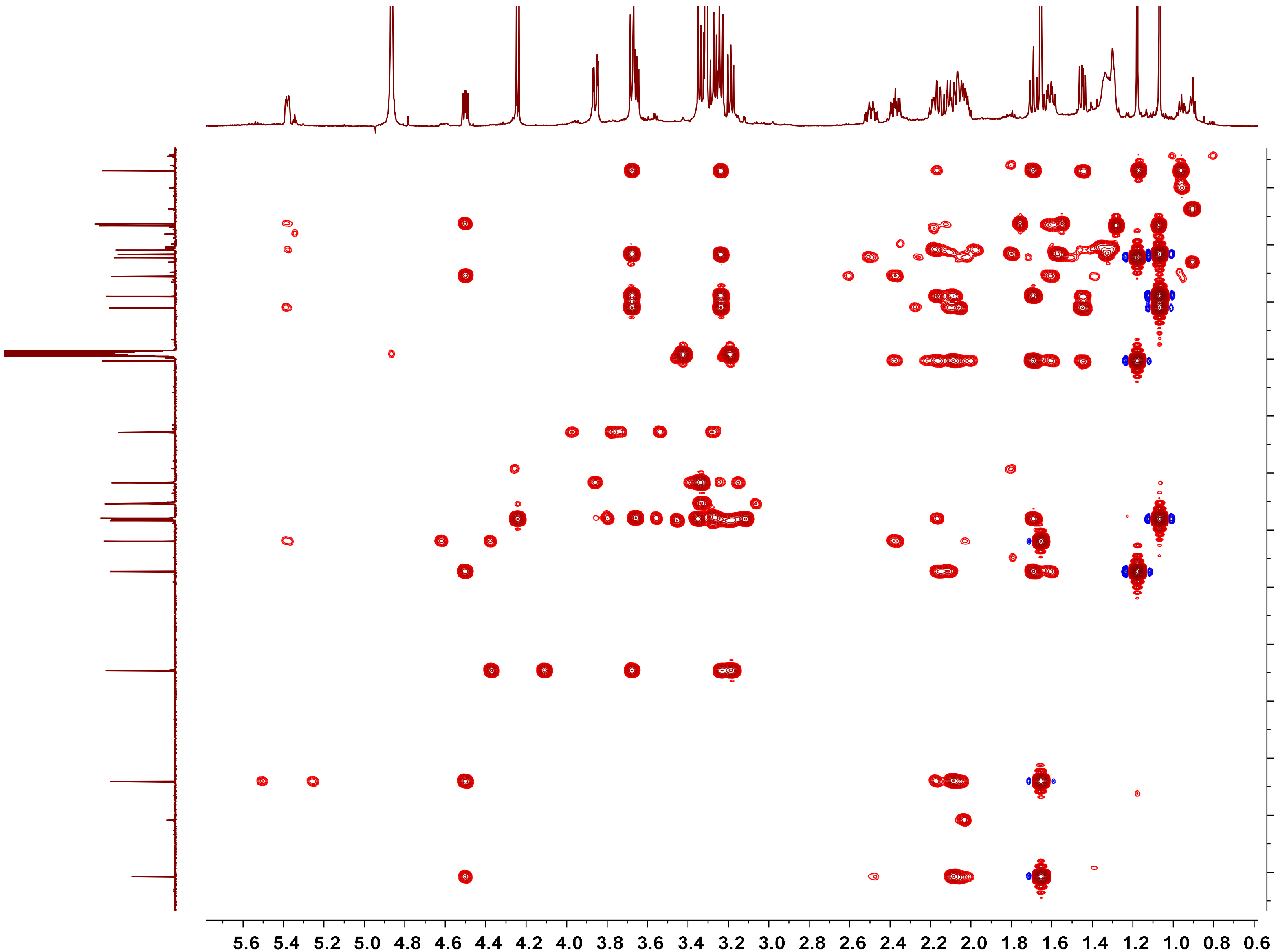

Figure S30. The HMBC spectrum of compound **3** in CD_3OD

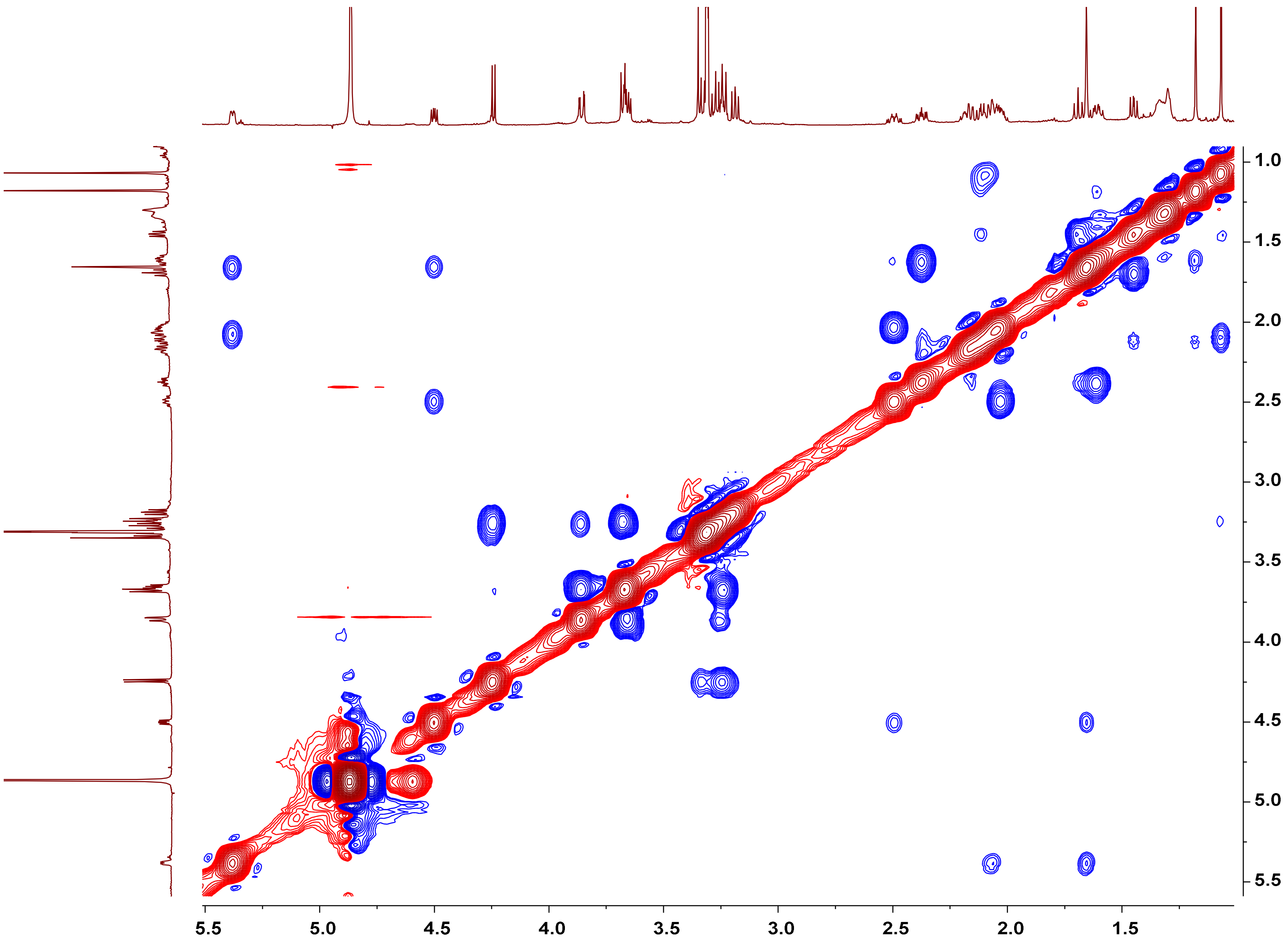

Figure S31. The NOESY spectrum of compound **3** in CD_3OD

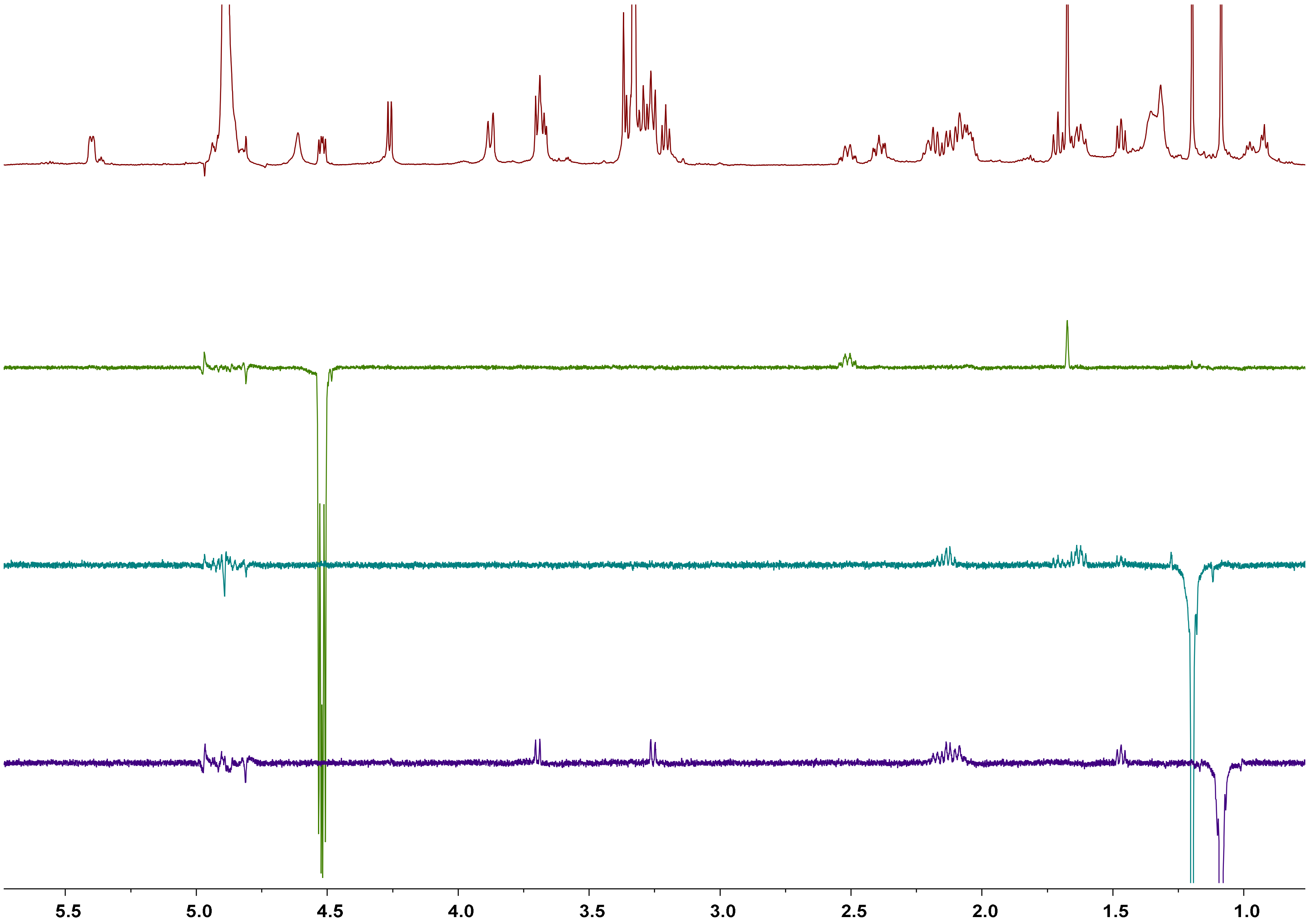

Figure S32. The 1D NOE spectrum of compound **3** in CD_3OD

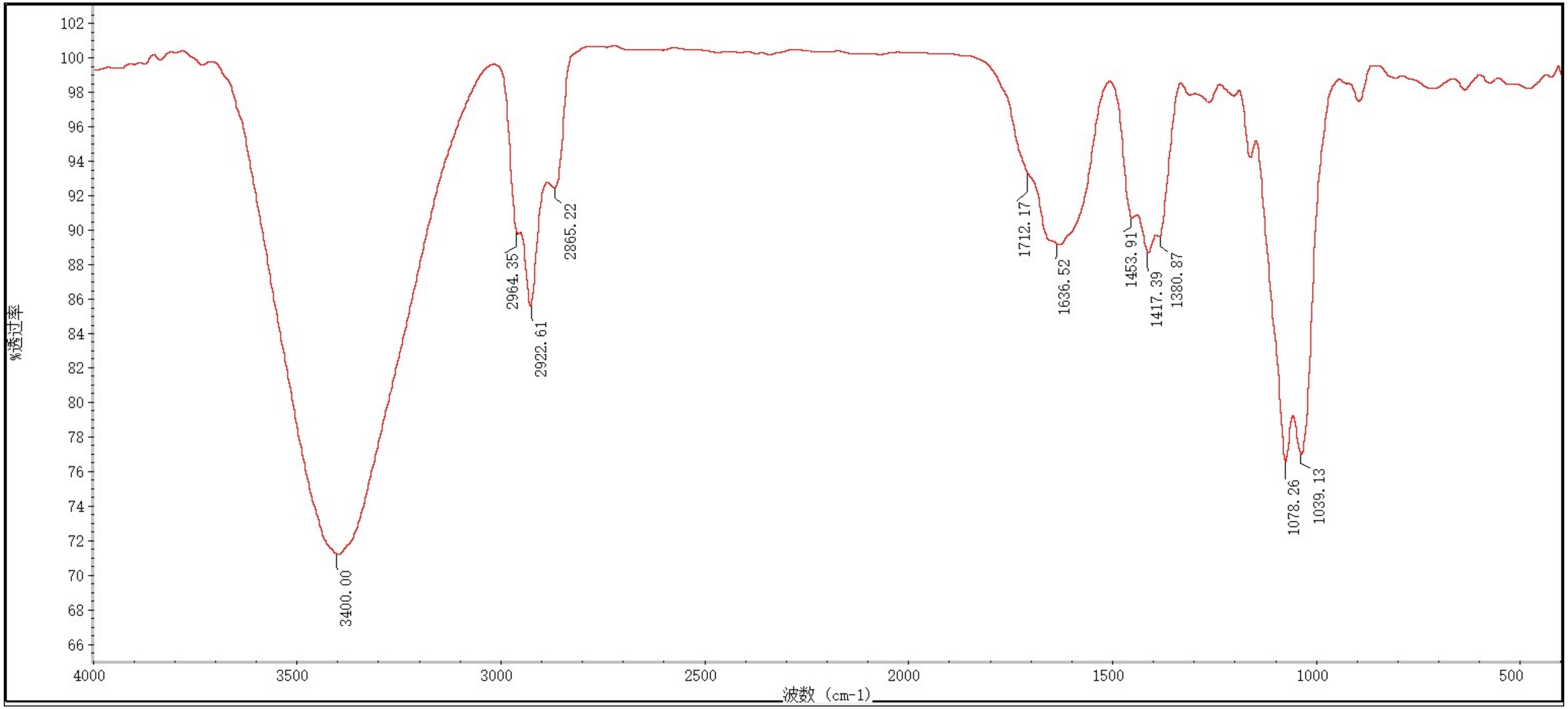

Figure S33. The IR spectrum of compound **3**

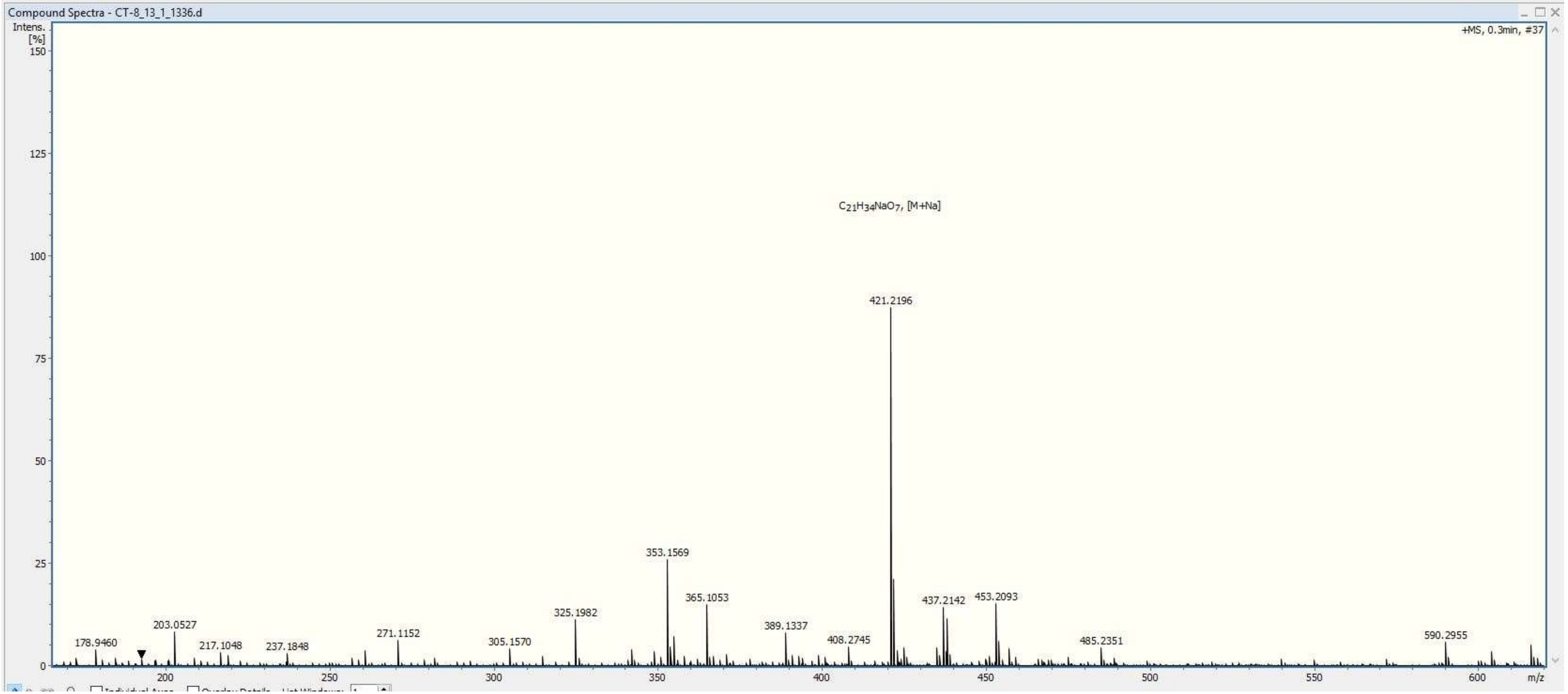

Figure S34. The (+)-HR-ESI-MS spectroscopic data of compound **3**

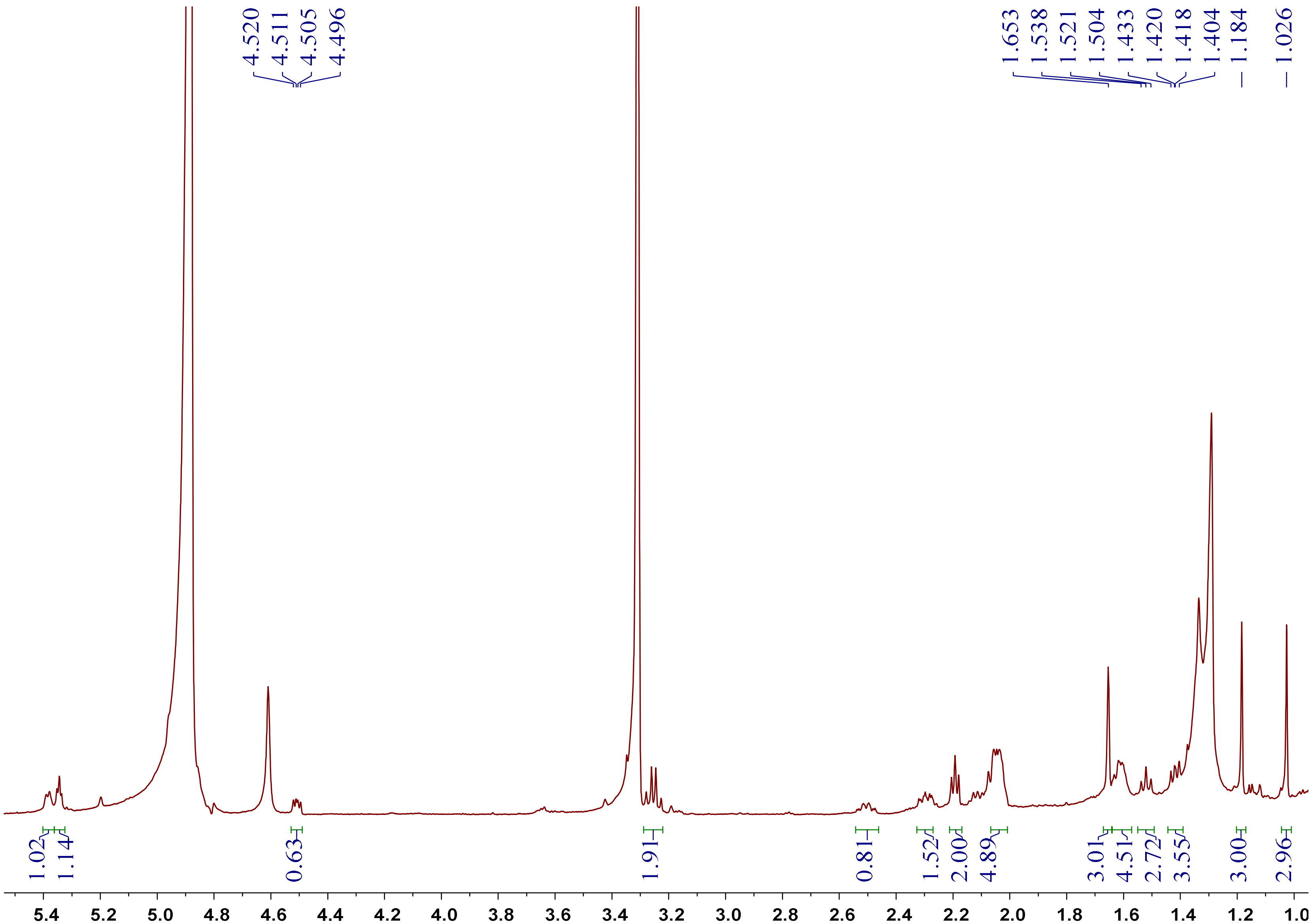

Figure S35. The ^{1}H NMR spectrum of compound **3a** in CD_3OD

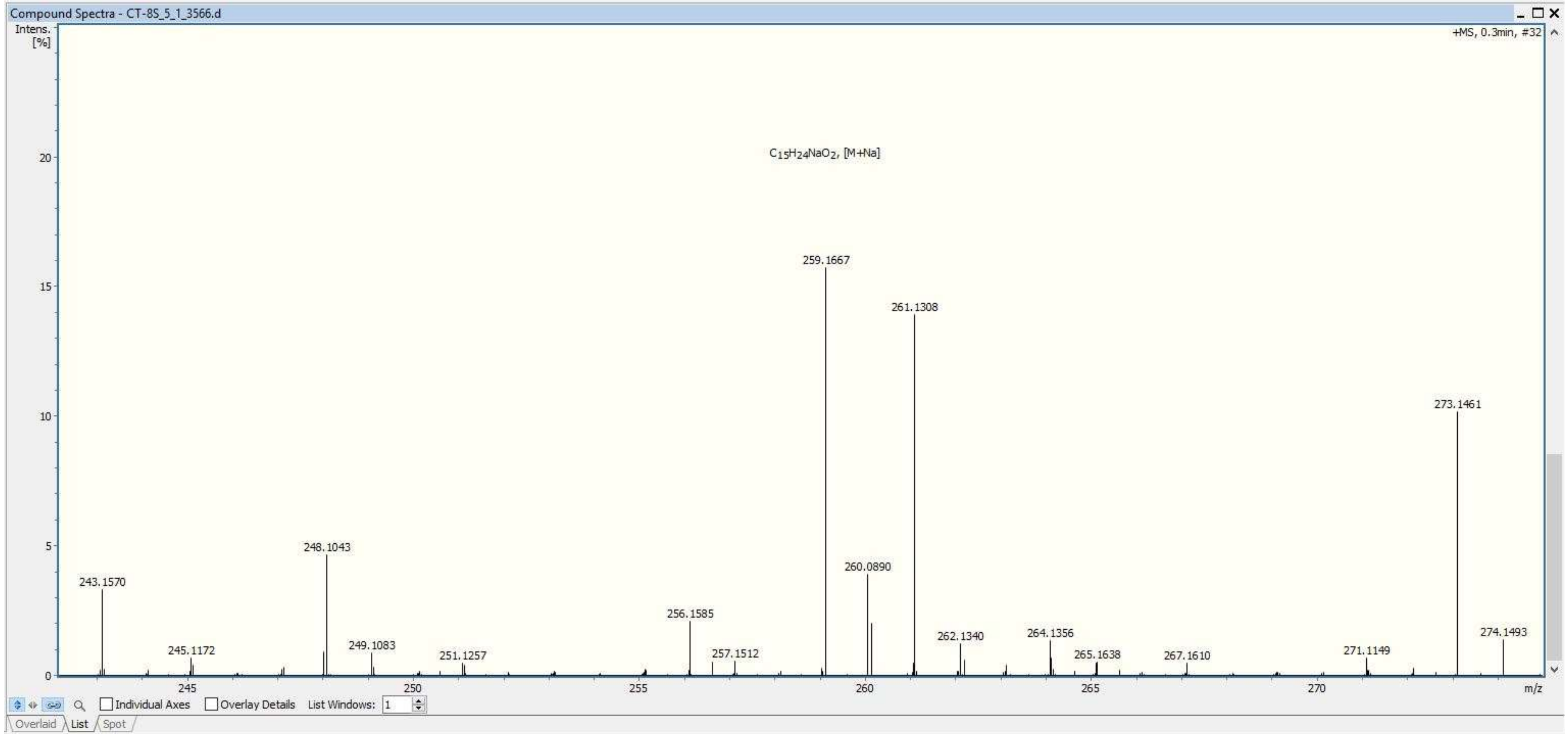

Figure S36. The (+)-HR-ESI-MS spectroscopic data of compound **3a**

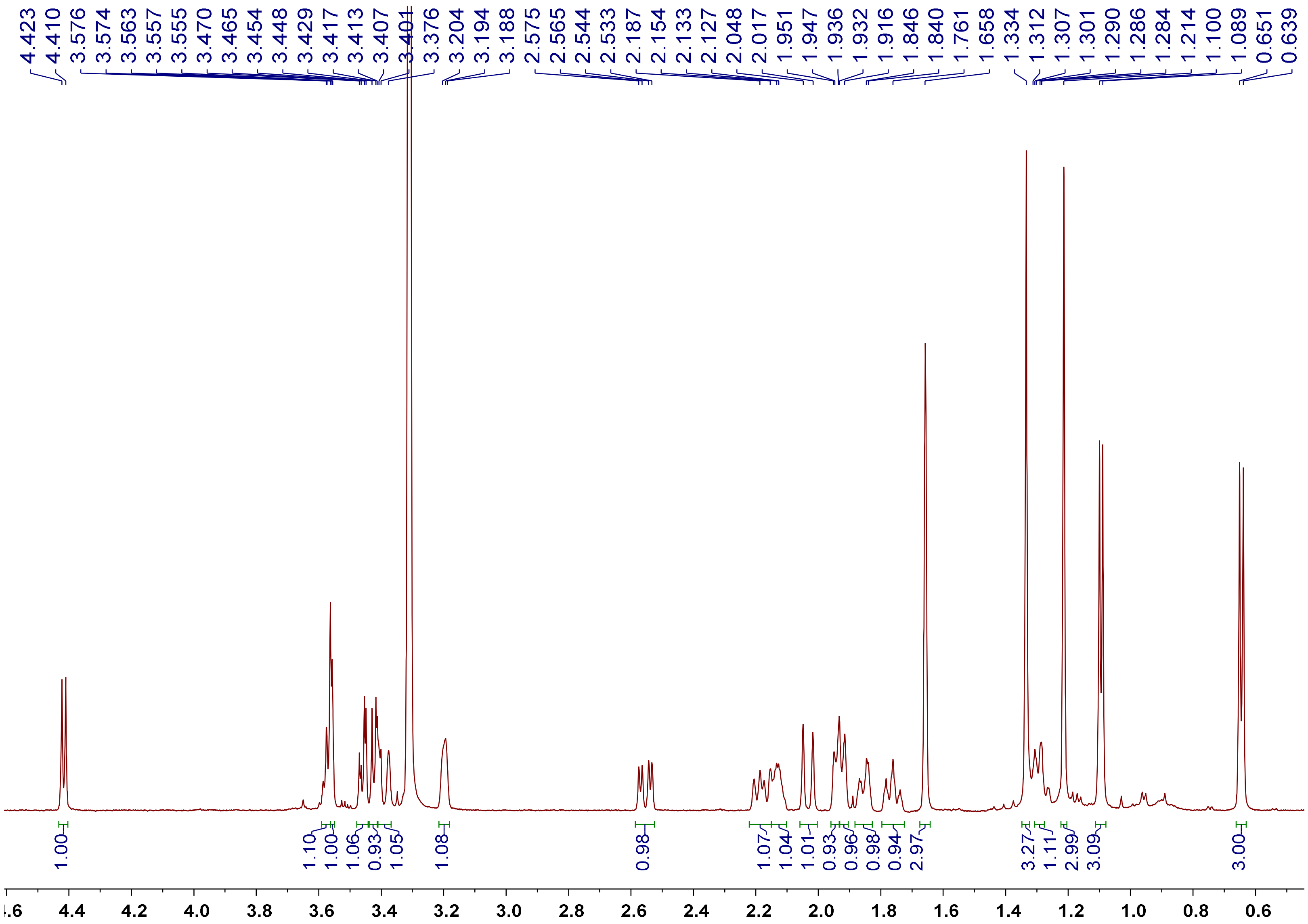

Figure S37. The ^{1}H NMR spectrum of compound **4** in CD_3OD

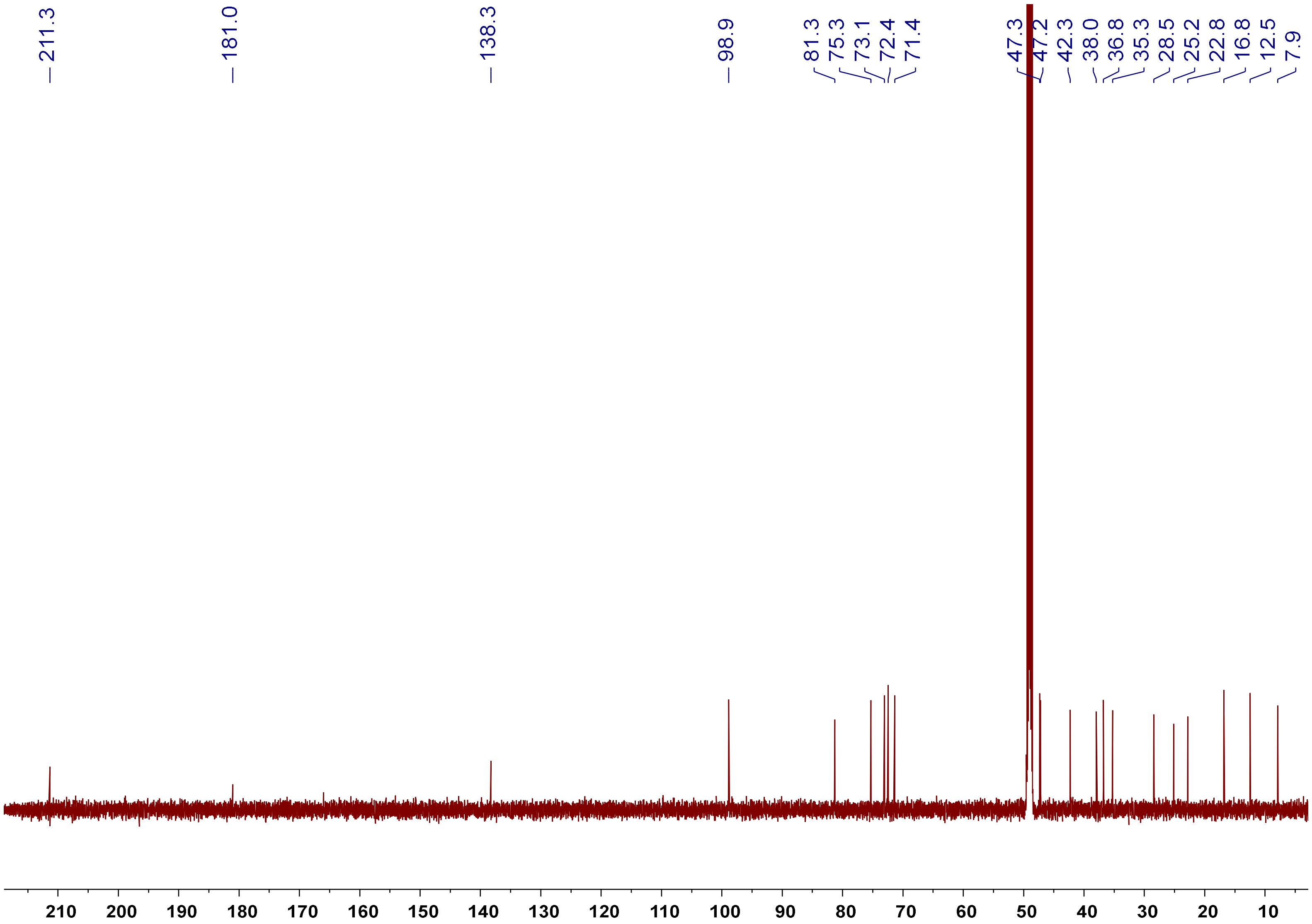

Figure S38. The ^{13}C NMR spectrum of compound **4** in CD_3OD

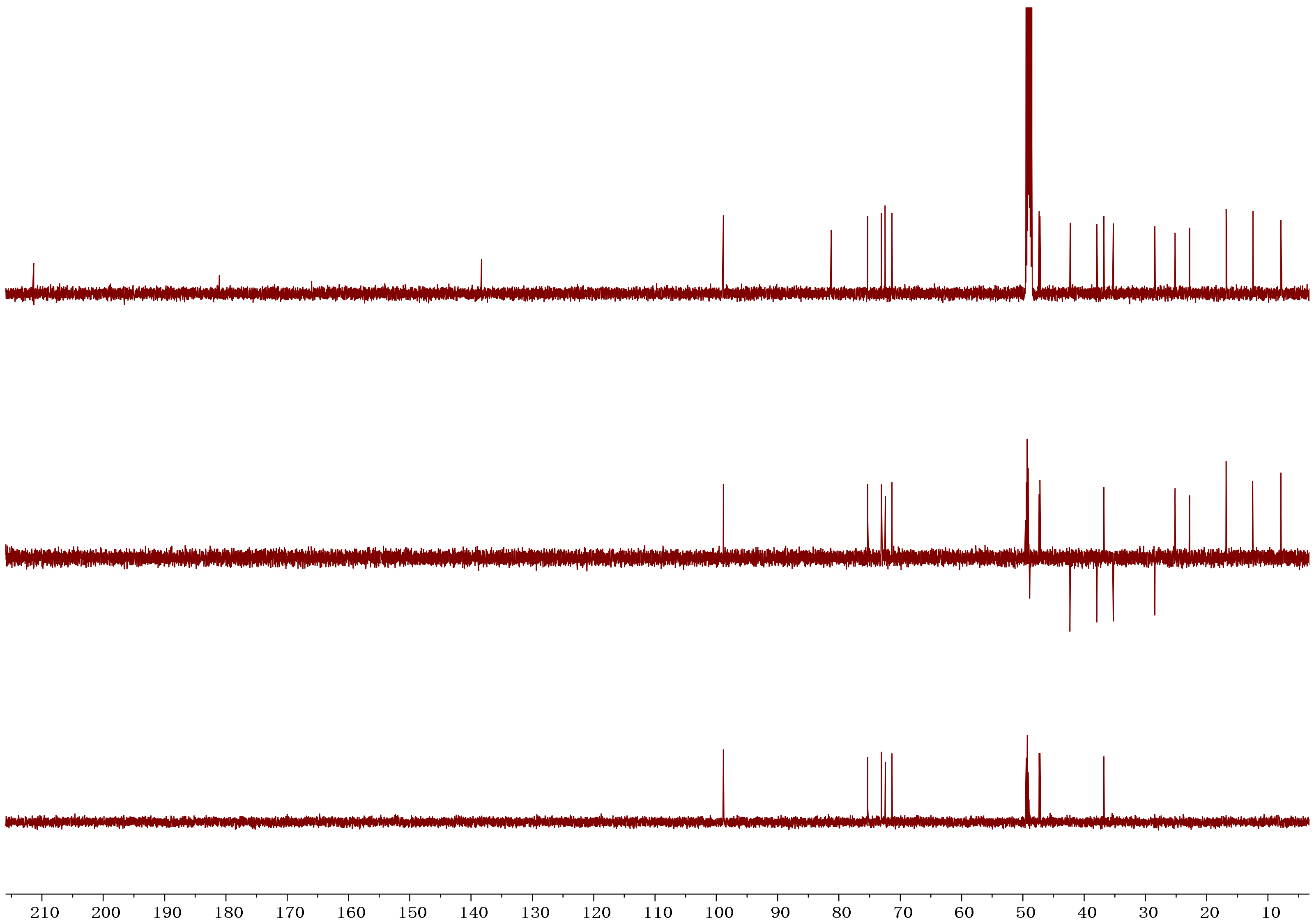

Figure S39. The DEPT spectrum of compound **4** in CD_3OD

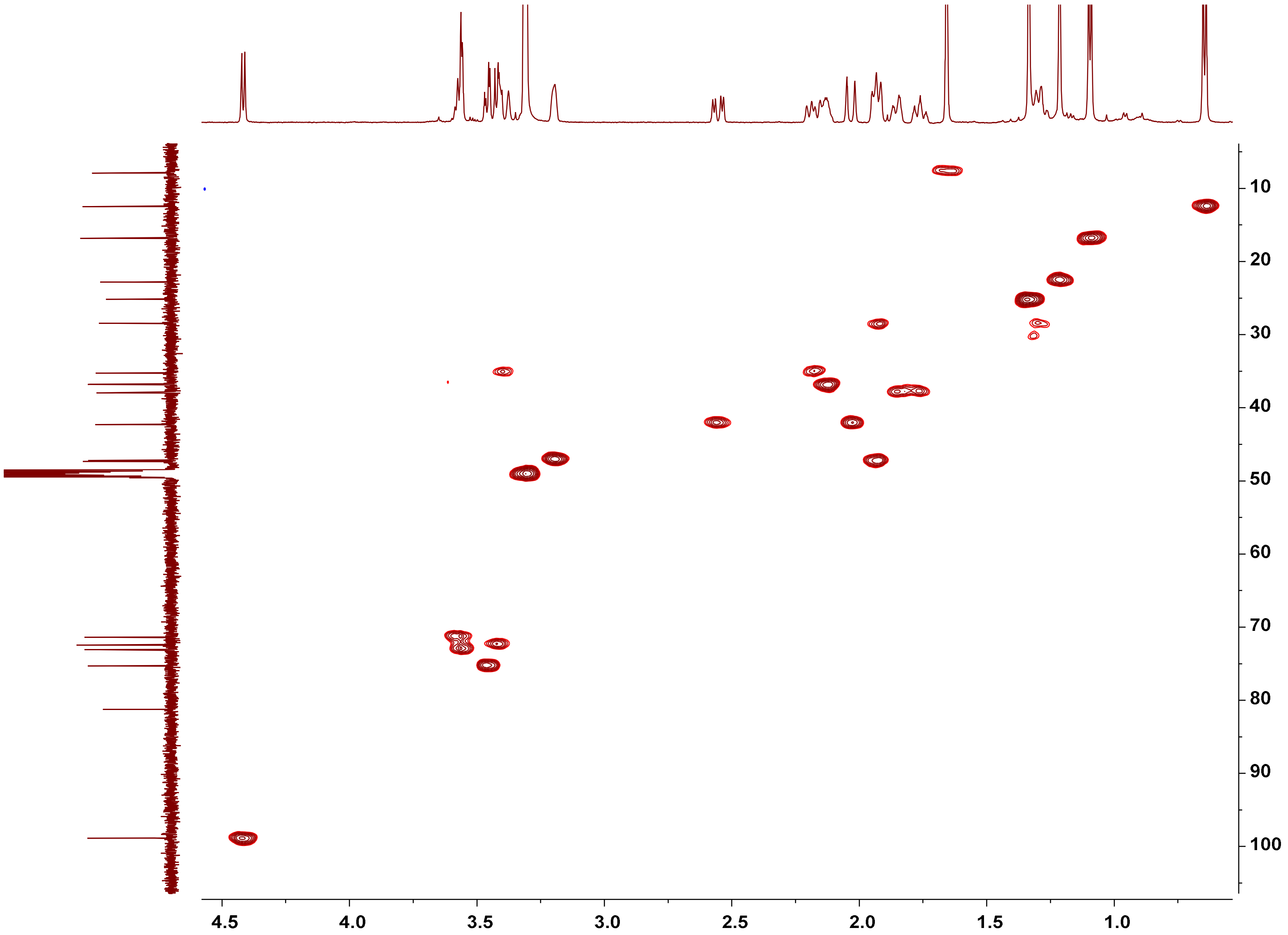

Figure S40. The HSQC spectrum of compound **4** in CD_3OD

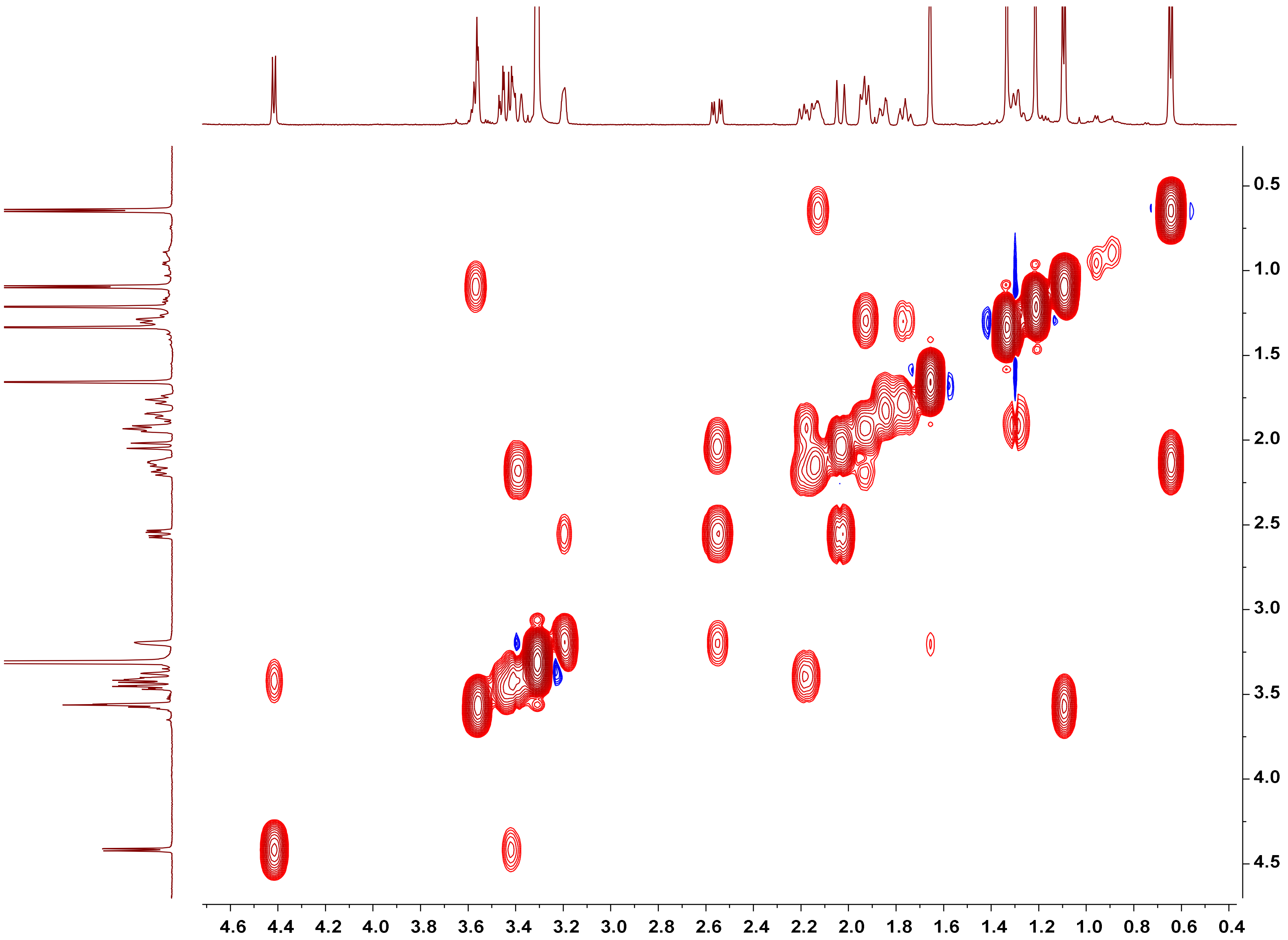

Figure S41. The ^{1}H-^{1}H COSY spectrum of compound **4** in CD_3OD

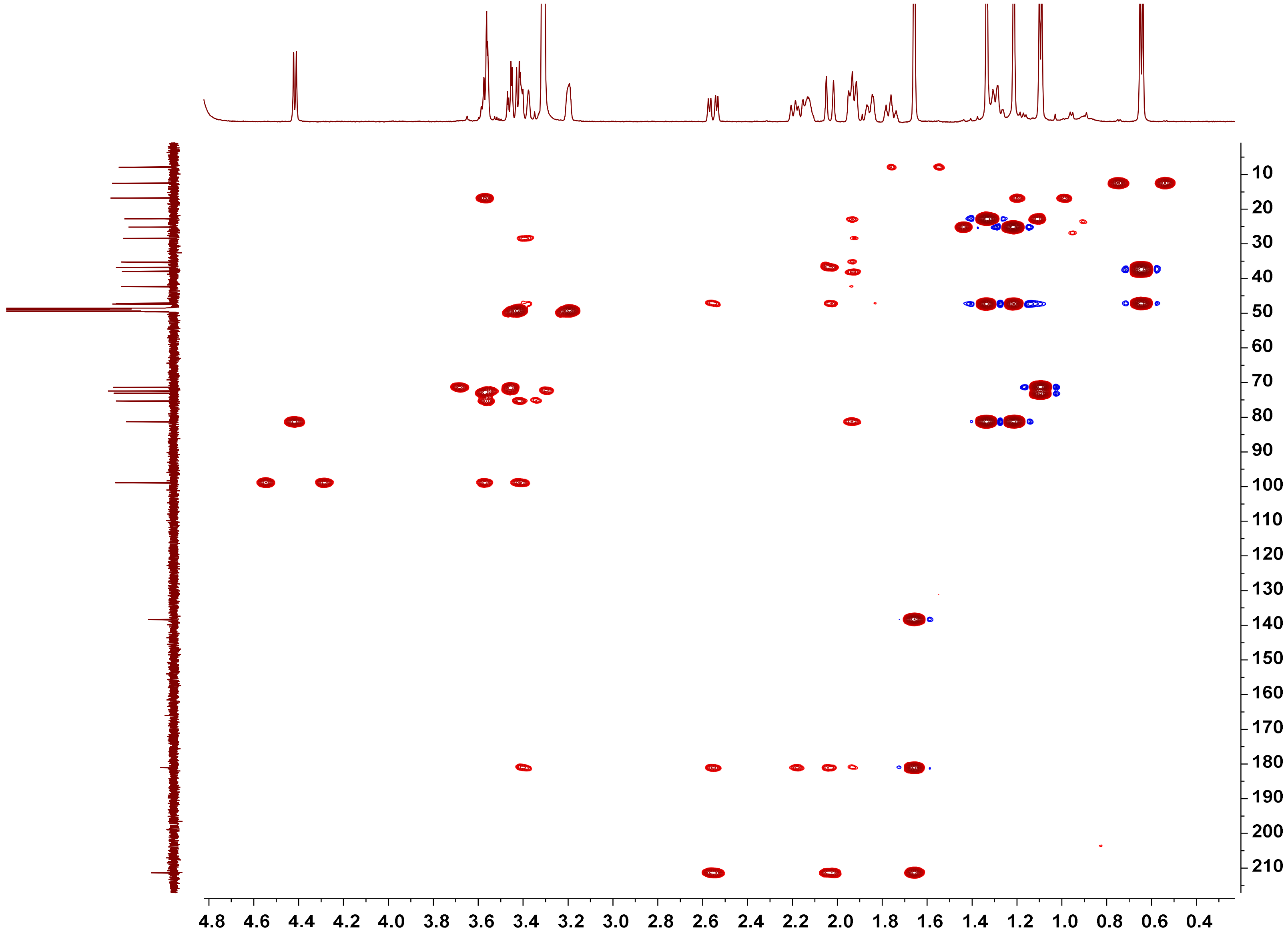

Figure S42. The HMBC spectrum of compound **4** in CD3OD

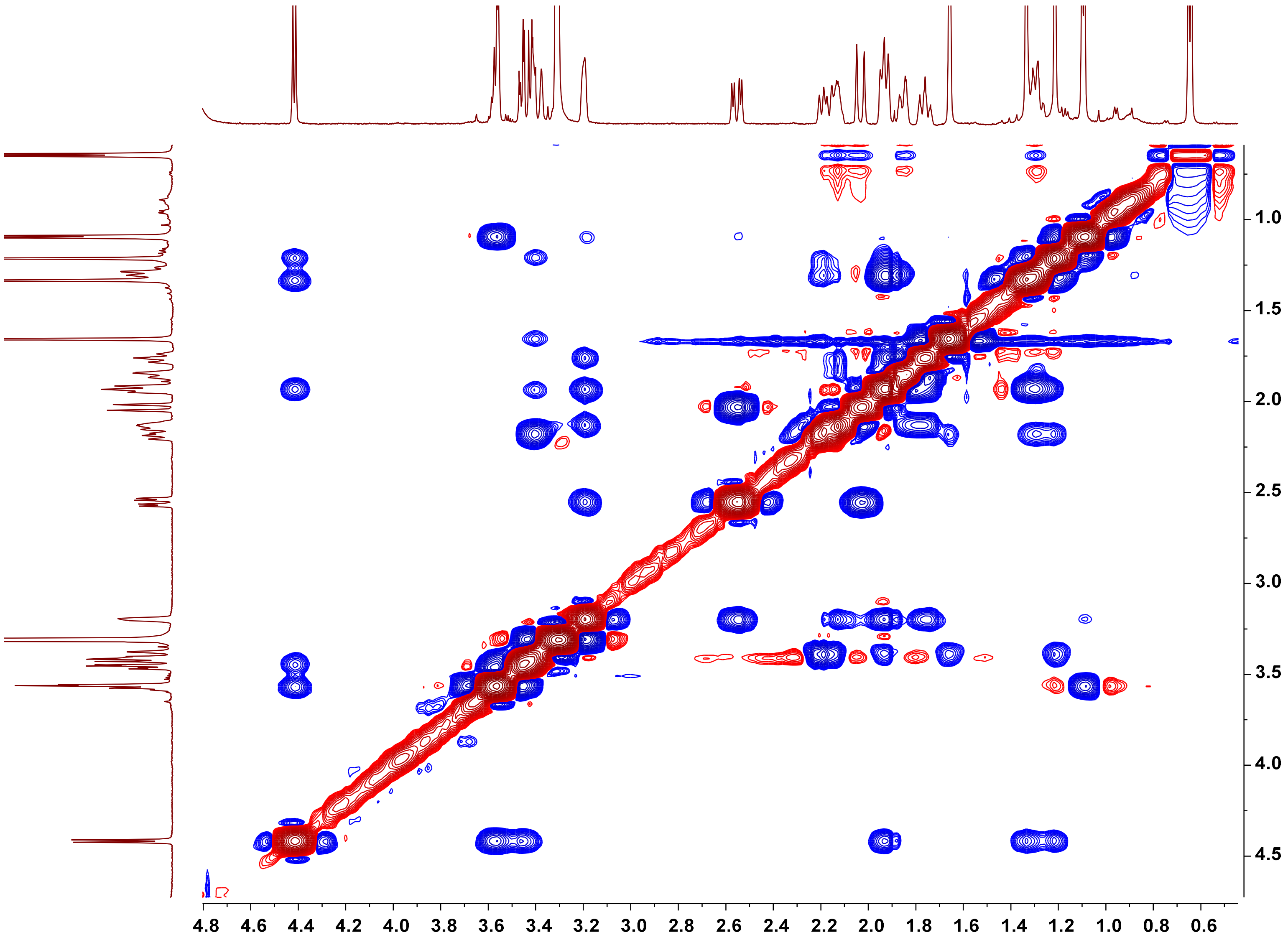

Figure S43. The NOESY spectrum of compound **4** in CD_3OD

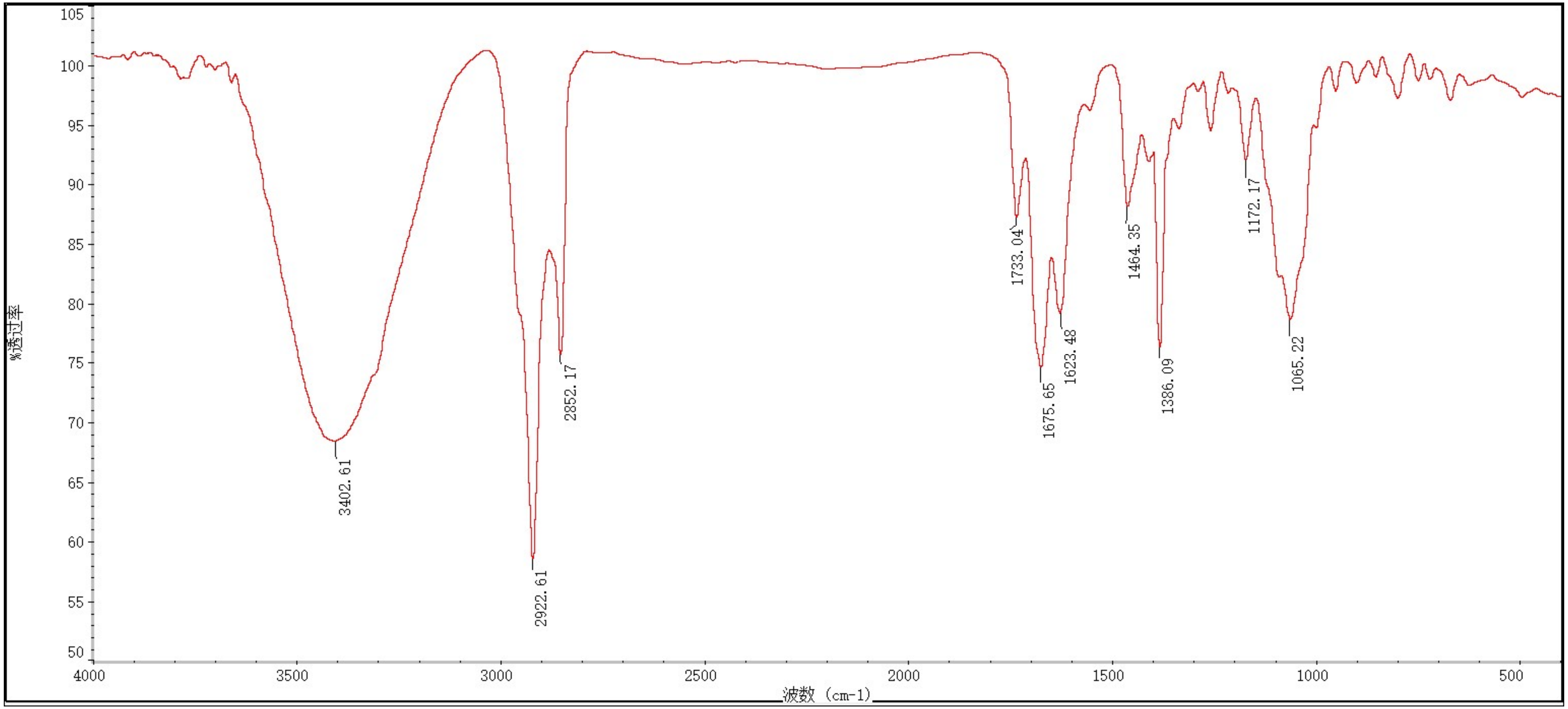

Figure S44. The IR spectrum of compound **4**

Mass	Calc. Mass	mDa	PPM	DBE	Formula	i-FIT	i-FIT Norm	Fit Conf %	C	H	O	Na
405.2244	405.2253	-0.9	-2.2	4.5	C21 H34 O6 Na	825.5	n/a	n/a	21	34	6	1

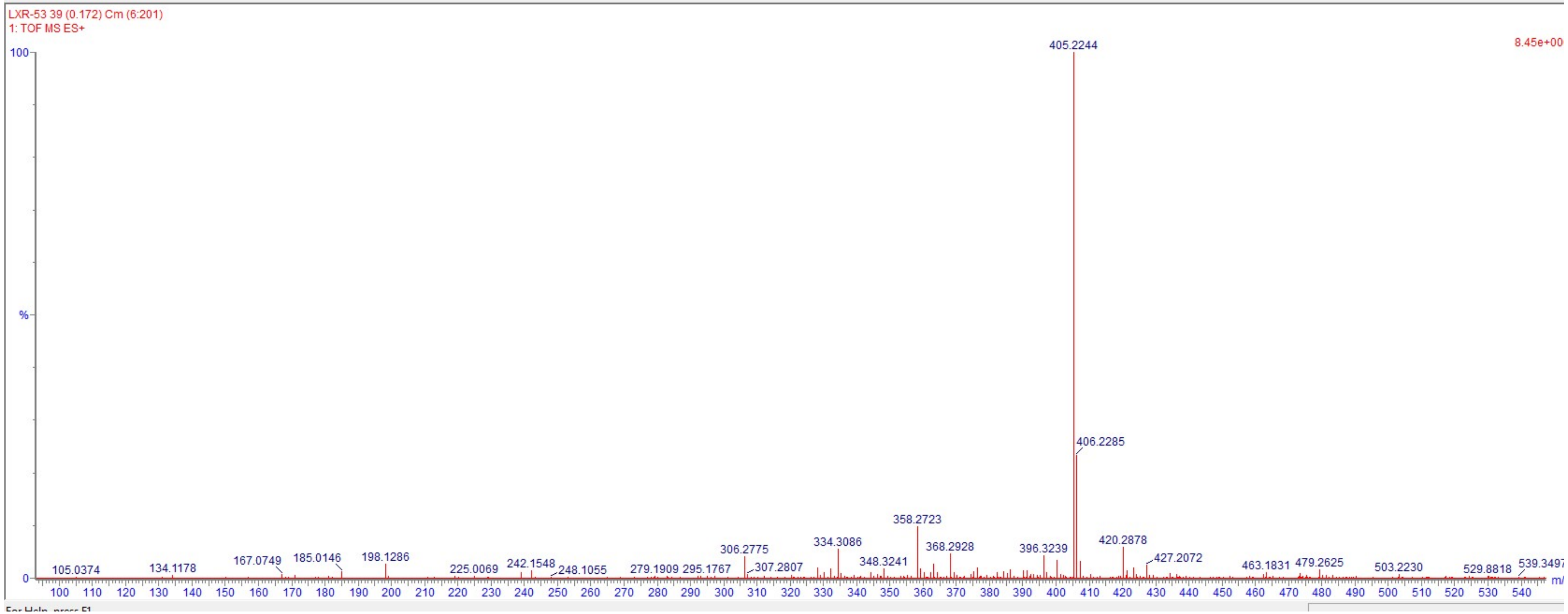

Figure S45. The (+)-HR-ESI-MS spectroscopic data of compound **4**

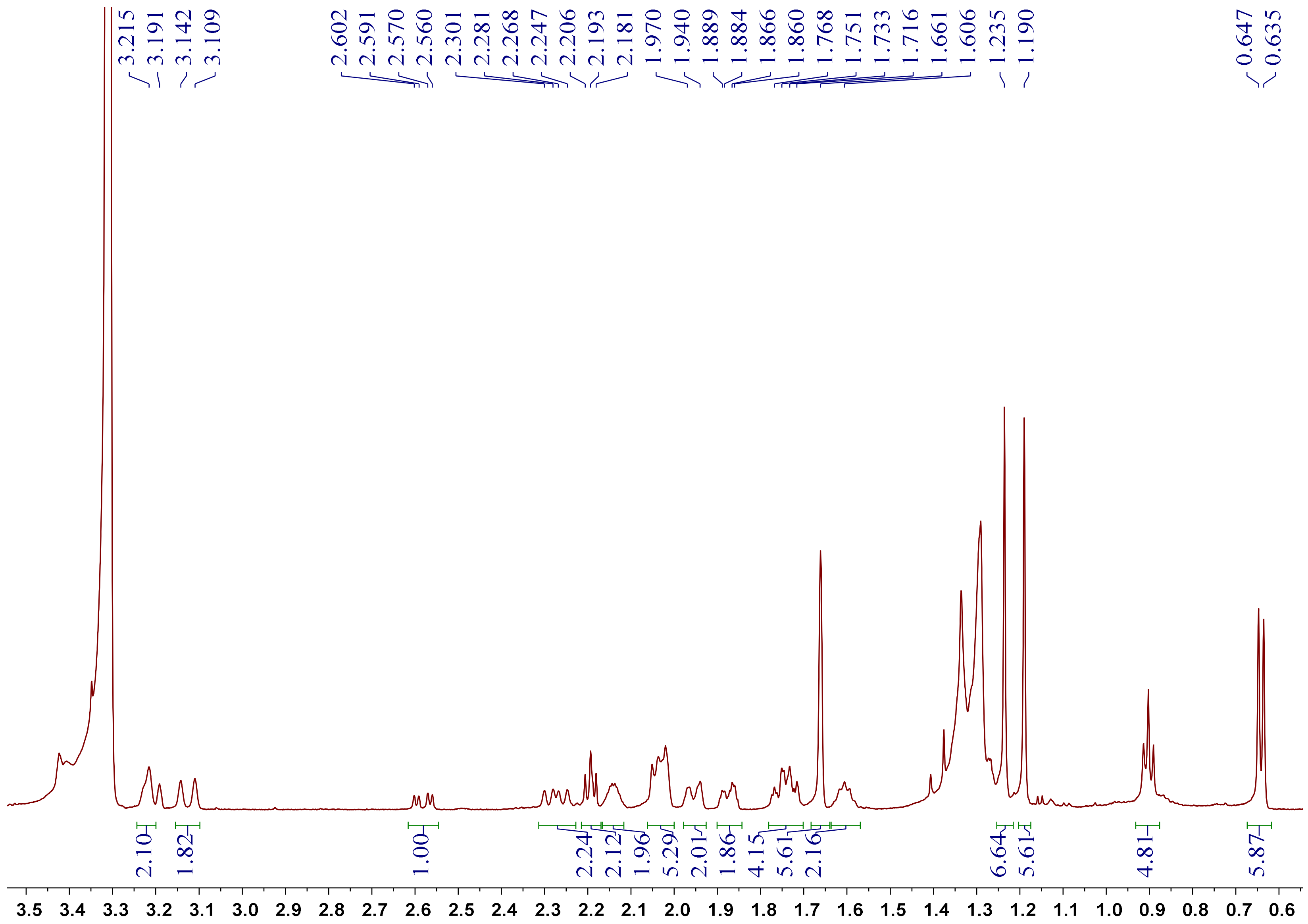

Figure S46. The ^{1}H NMR spectrum of compound **4a** in CD_3OD

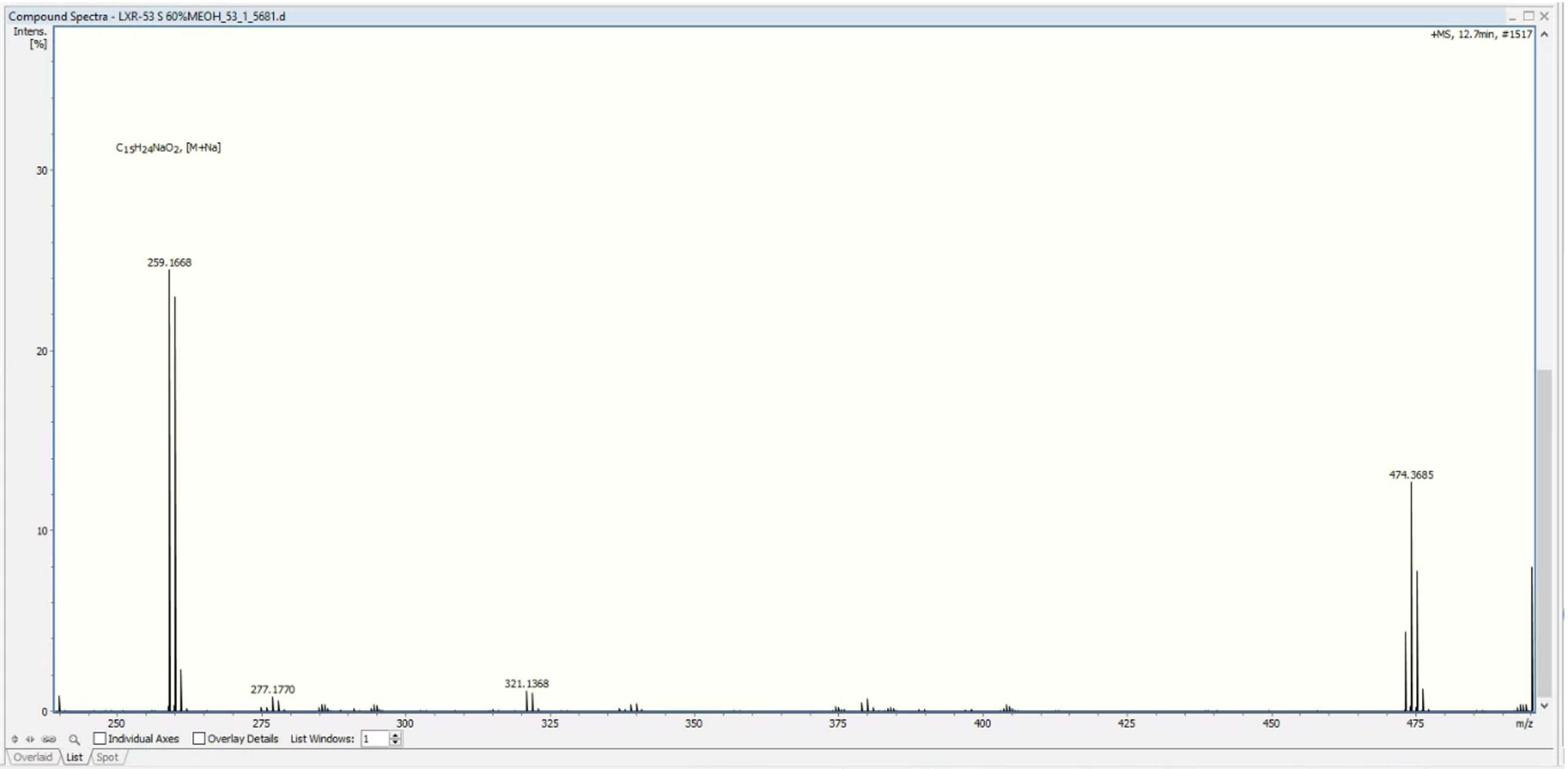

Figure S47. The (+)-HR-ESI-MS spectroscopic data of compound **4a**

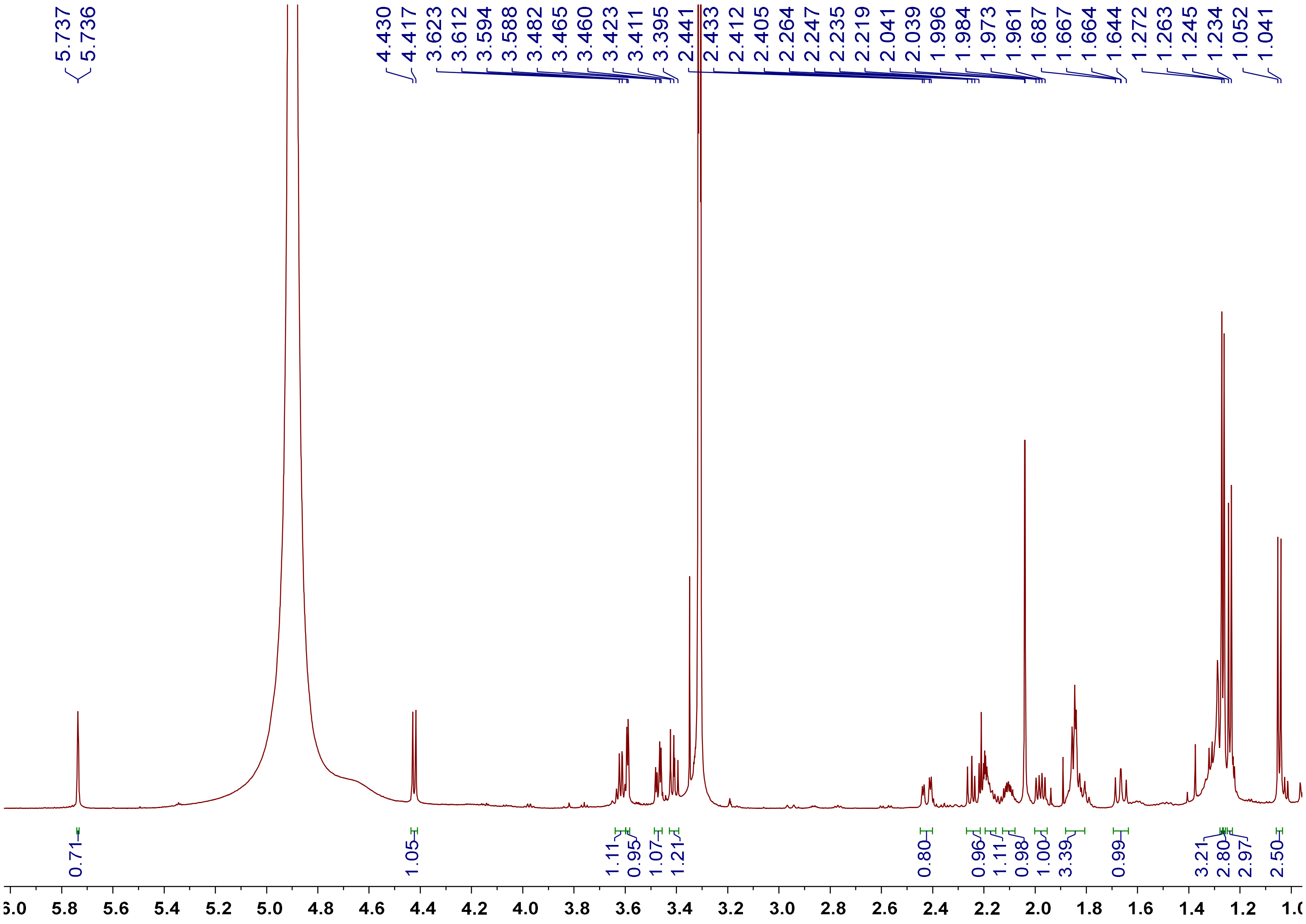

Figure S48. The ^{1}H NMR spectrum of compound **5** in CD_3OD

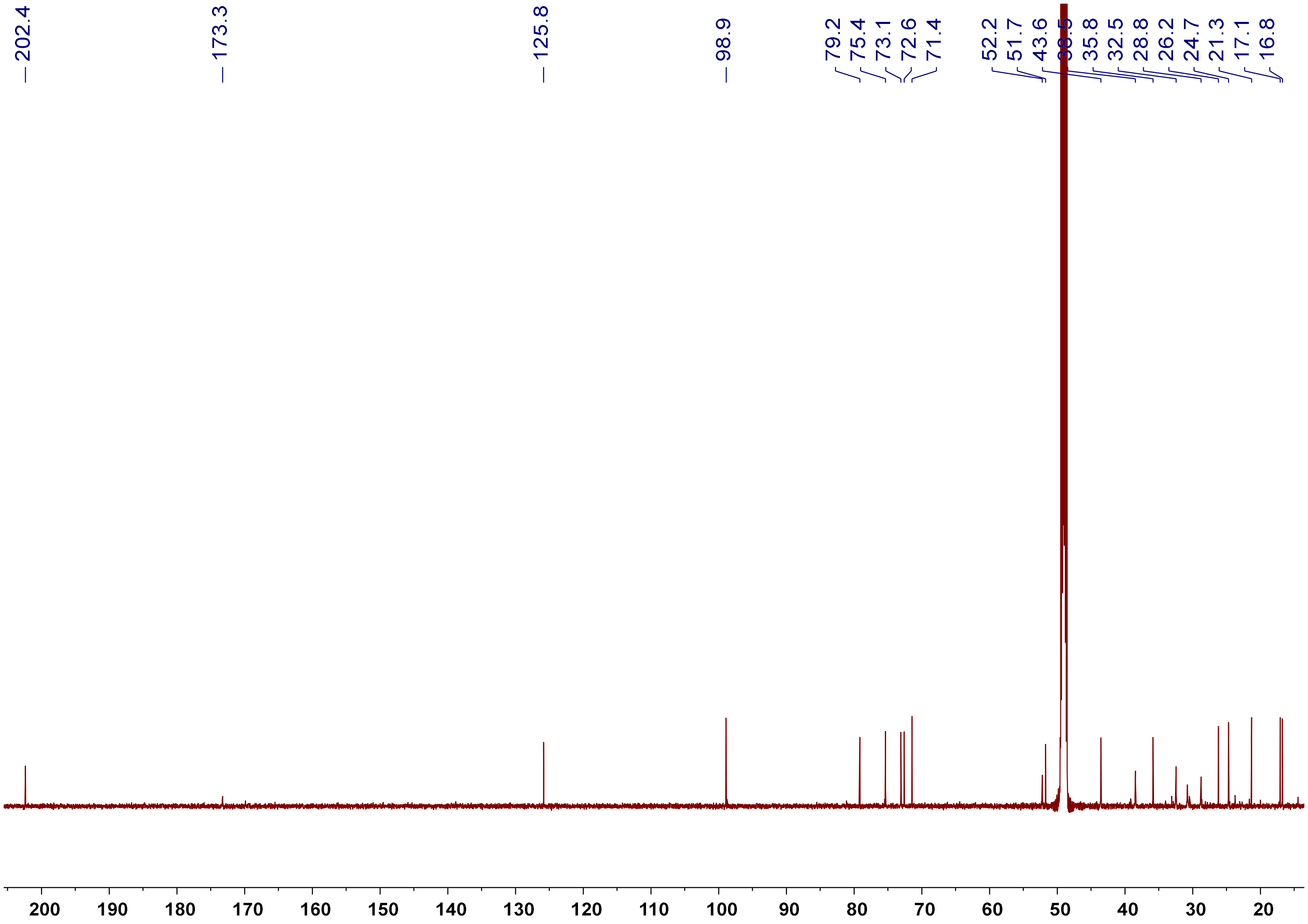

Figure S49. The ^{13}C NMR spectrum of compound **5** in CD_3OD

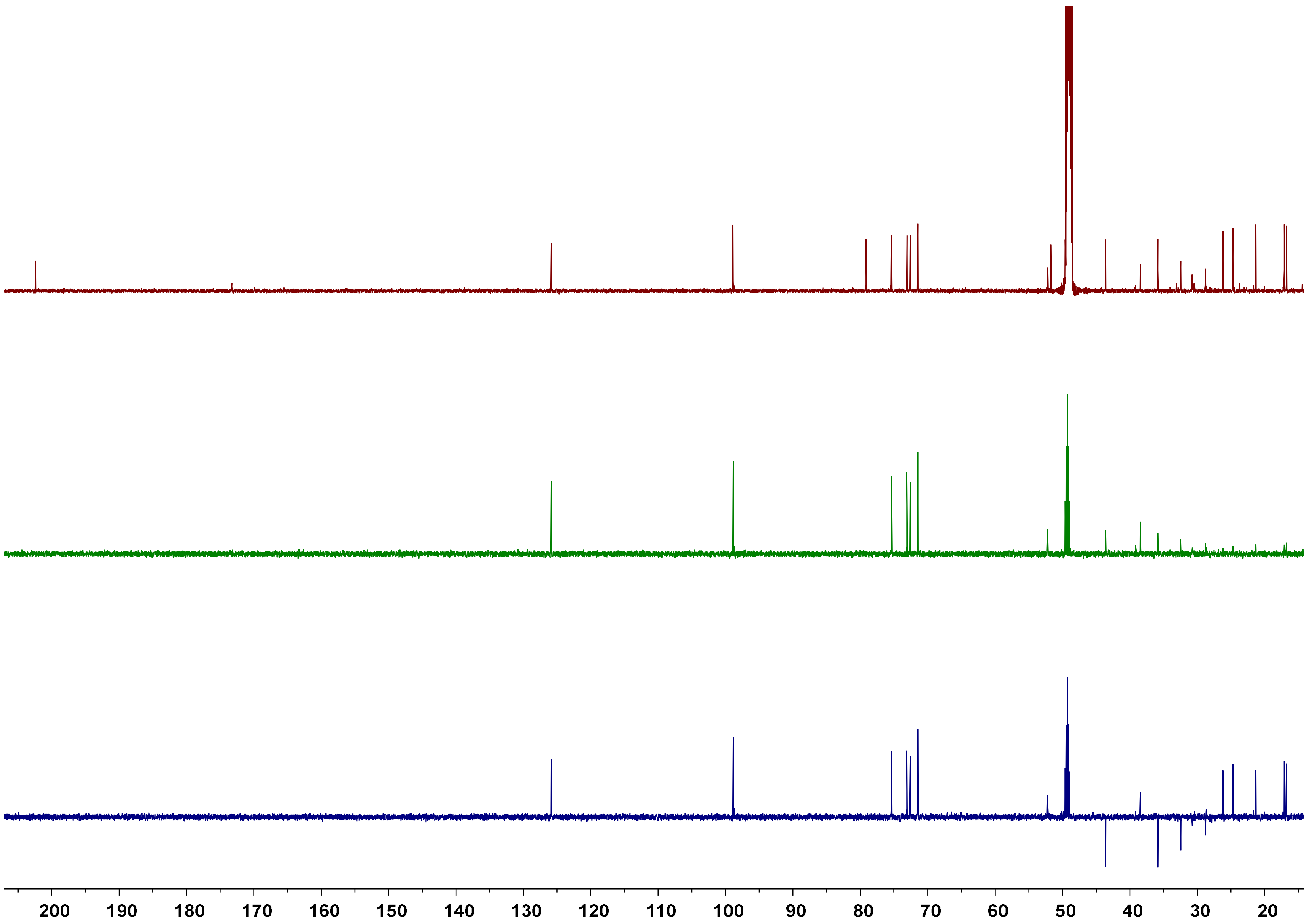

Figure S50. The DEPT spectrum of compound **5** in CD_3OD

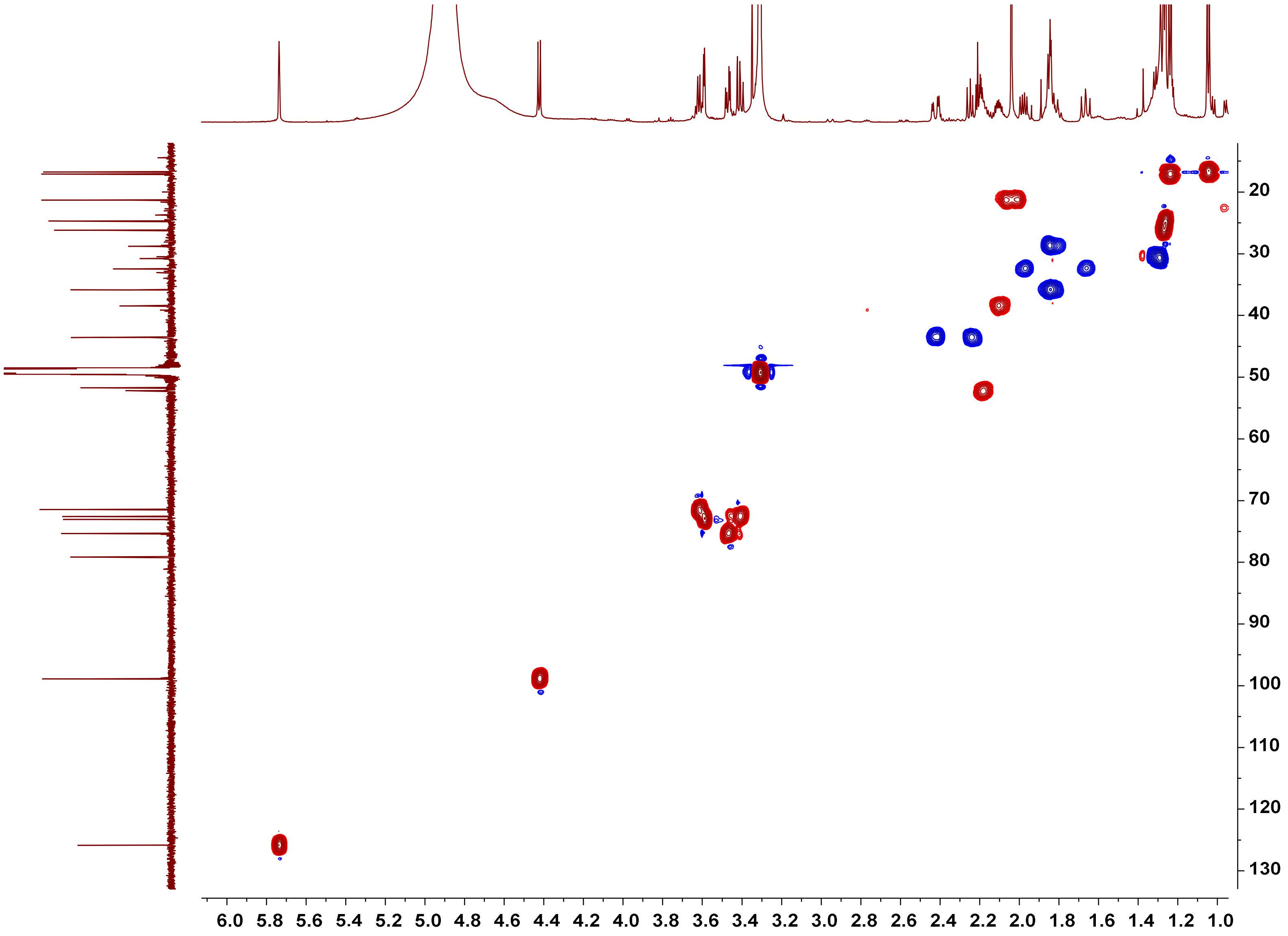

Figure S51. The HSQC spectrum of compound **5** in CD_3OD

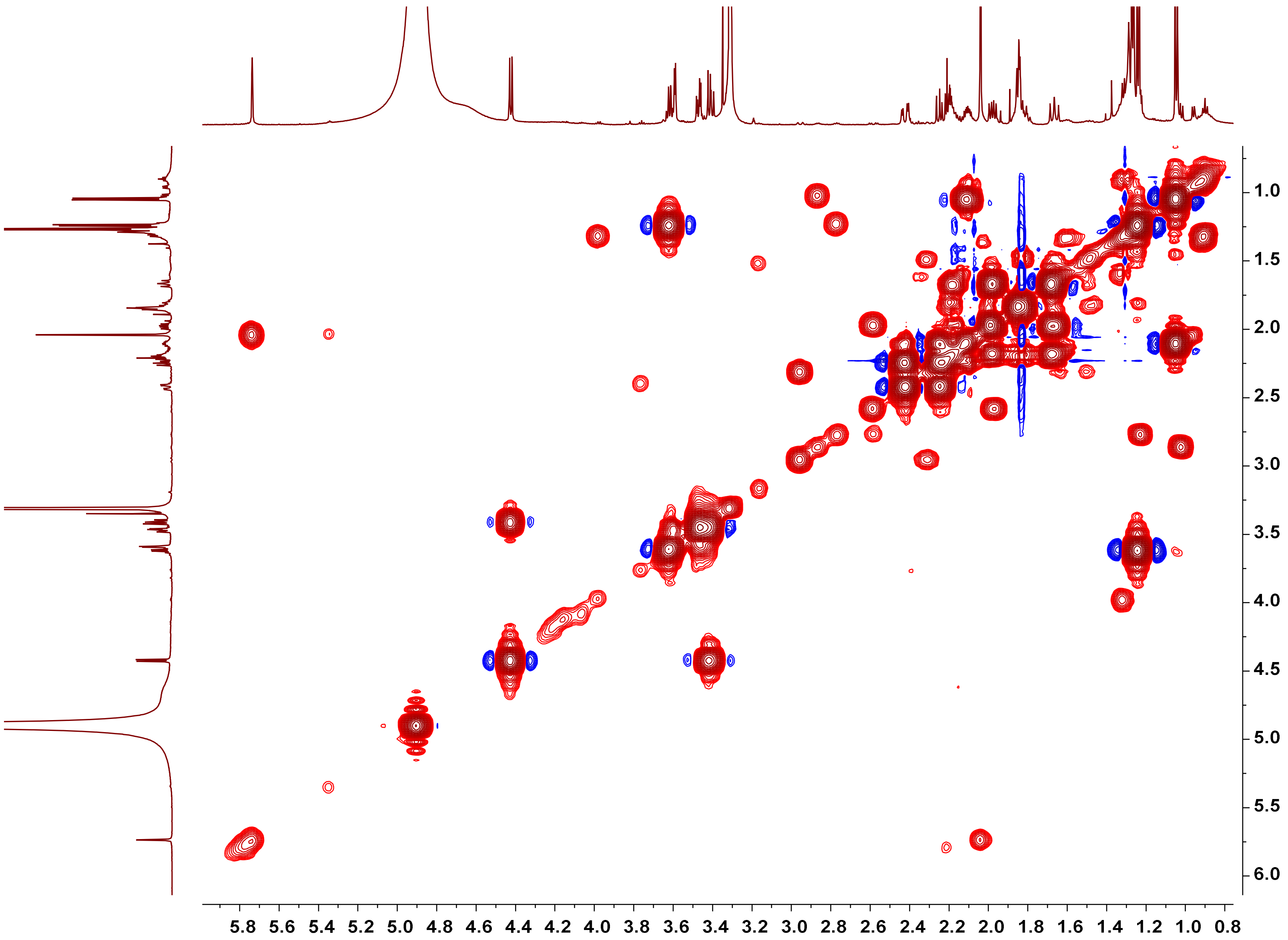

Figure S52. The ^{1}H-^{1}H COSY spectrum of compound **5** in CD_3OD

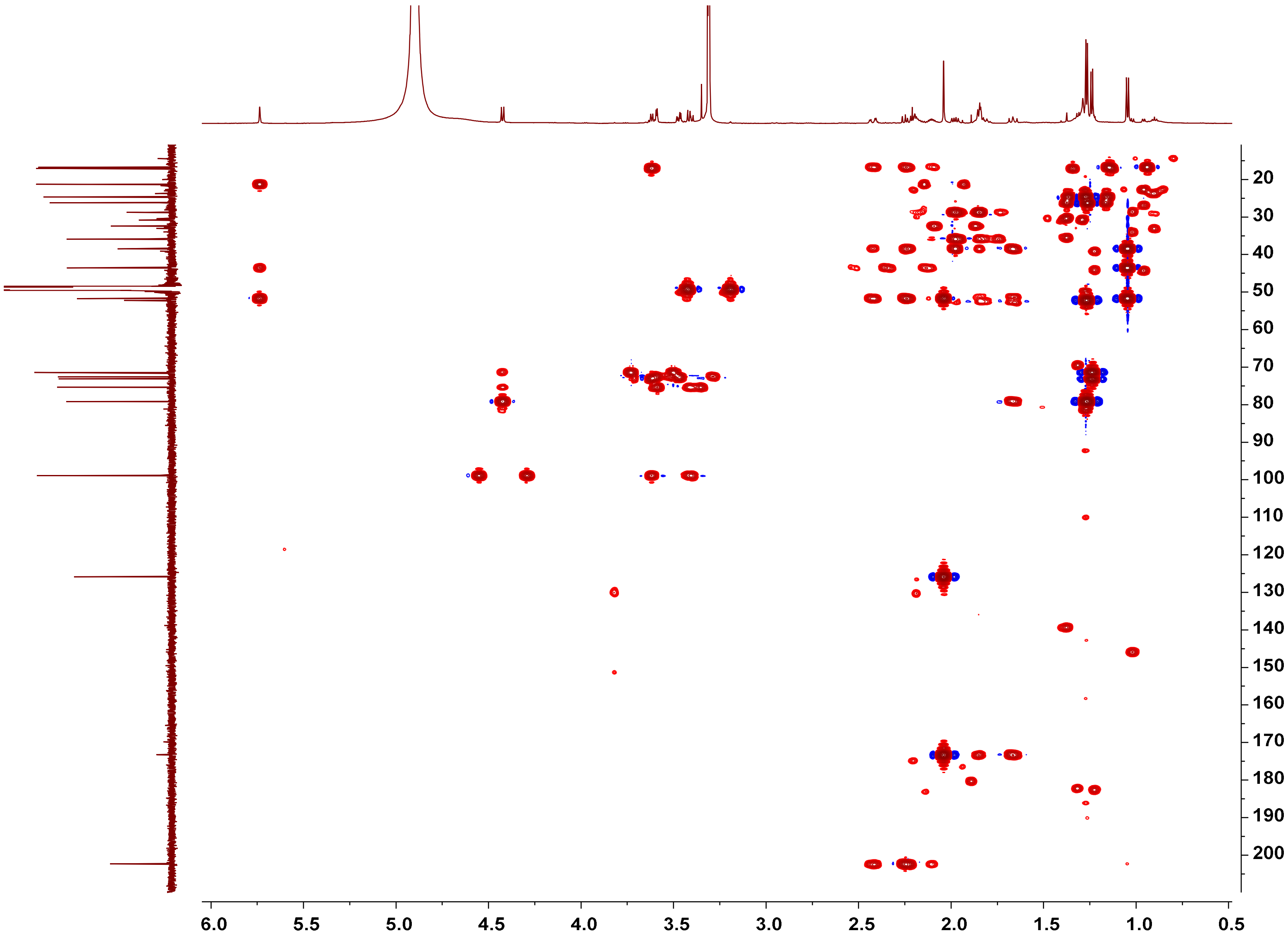

Figure S53. The HMBC spectrum of compound **5** in CD_3OD

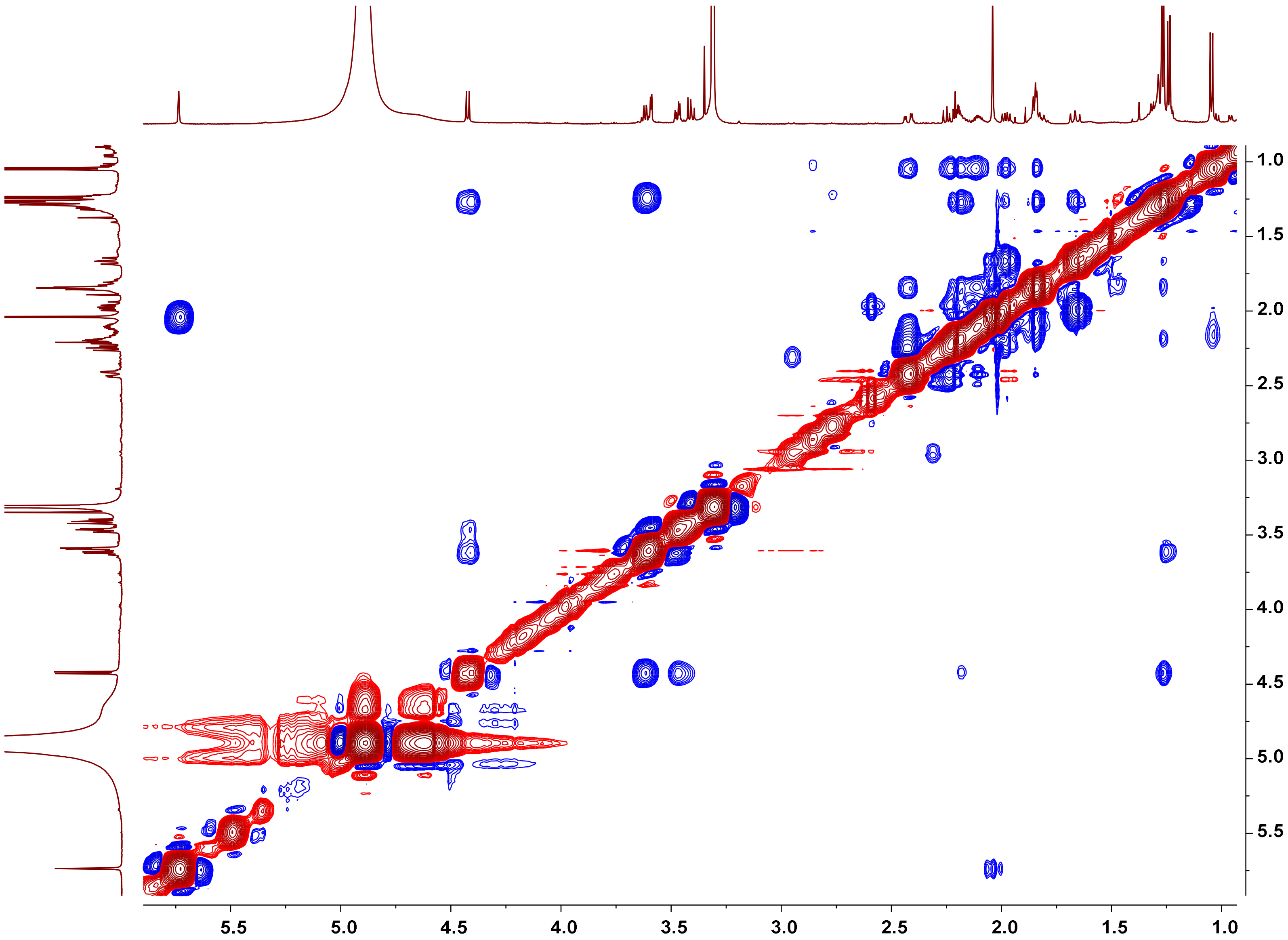

Figure S54. The NOESY spectrum of compound **5** in CD_3OD

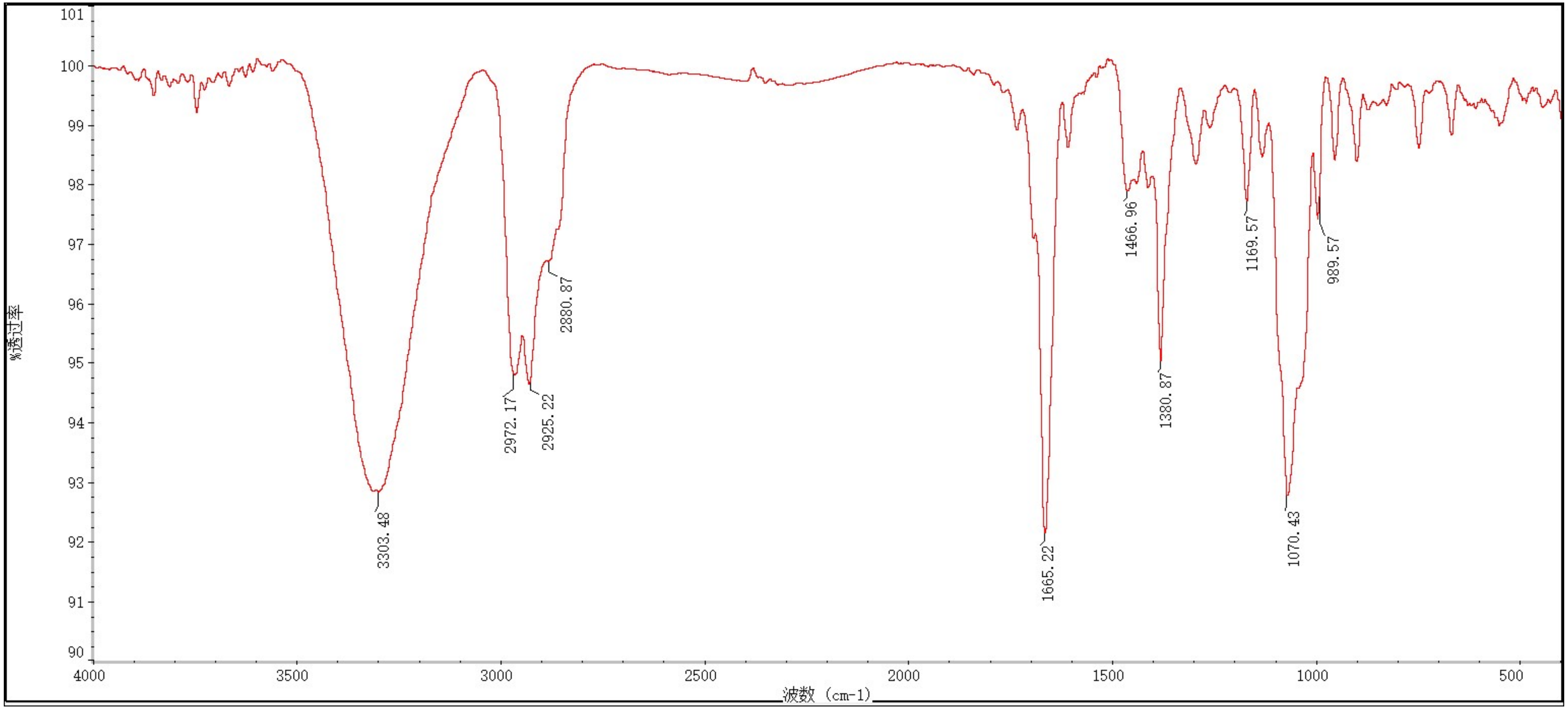

Figure S55. The IR spectrum of compound **5**

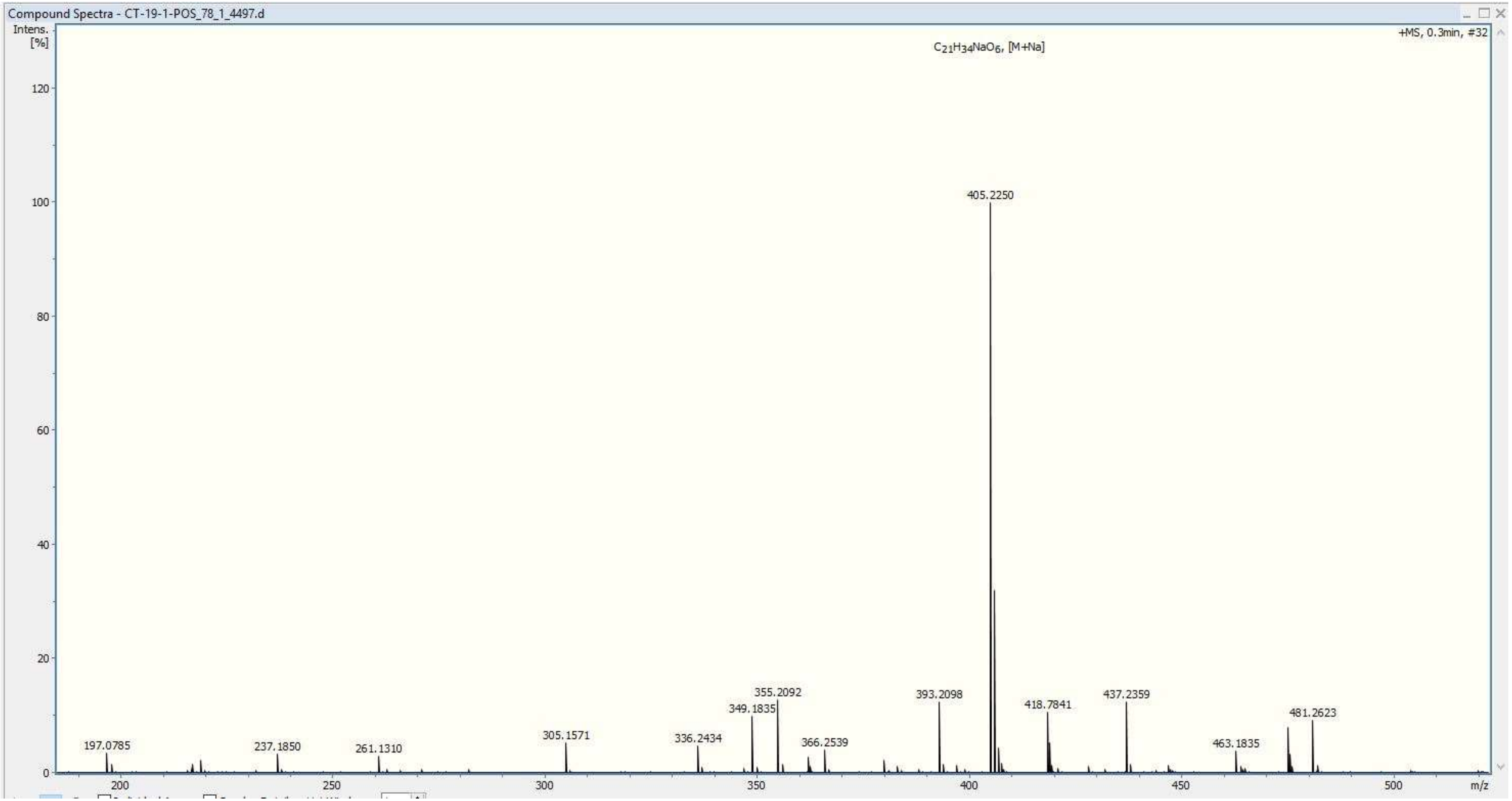

Figure S56. The (+)-HR-ESI-MS spectroscopic data of compound **5**

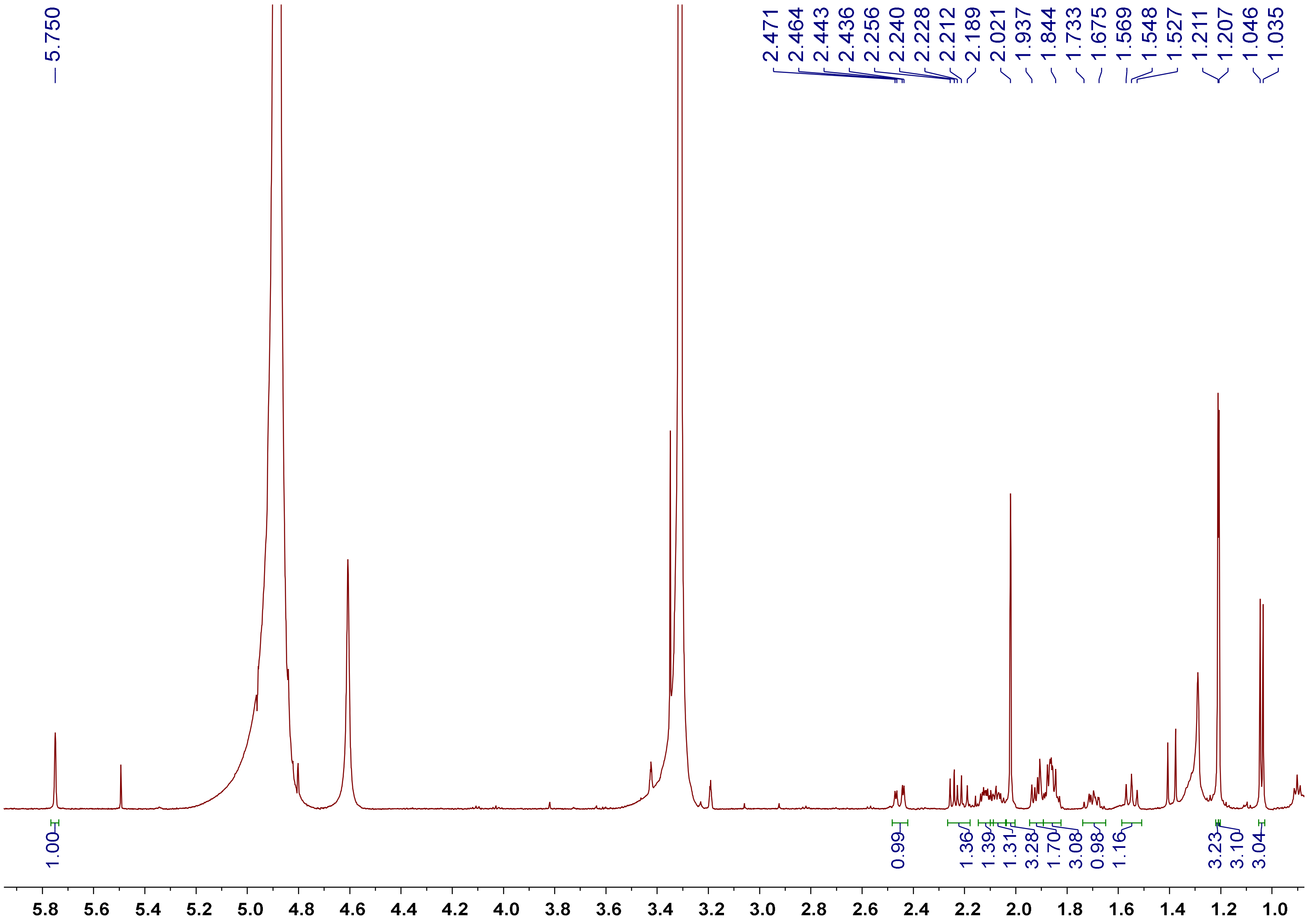

Figure S57. The ^{1}H NMR spectrum of compound **5a** in CD_3OD

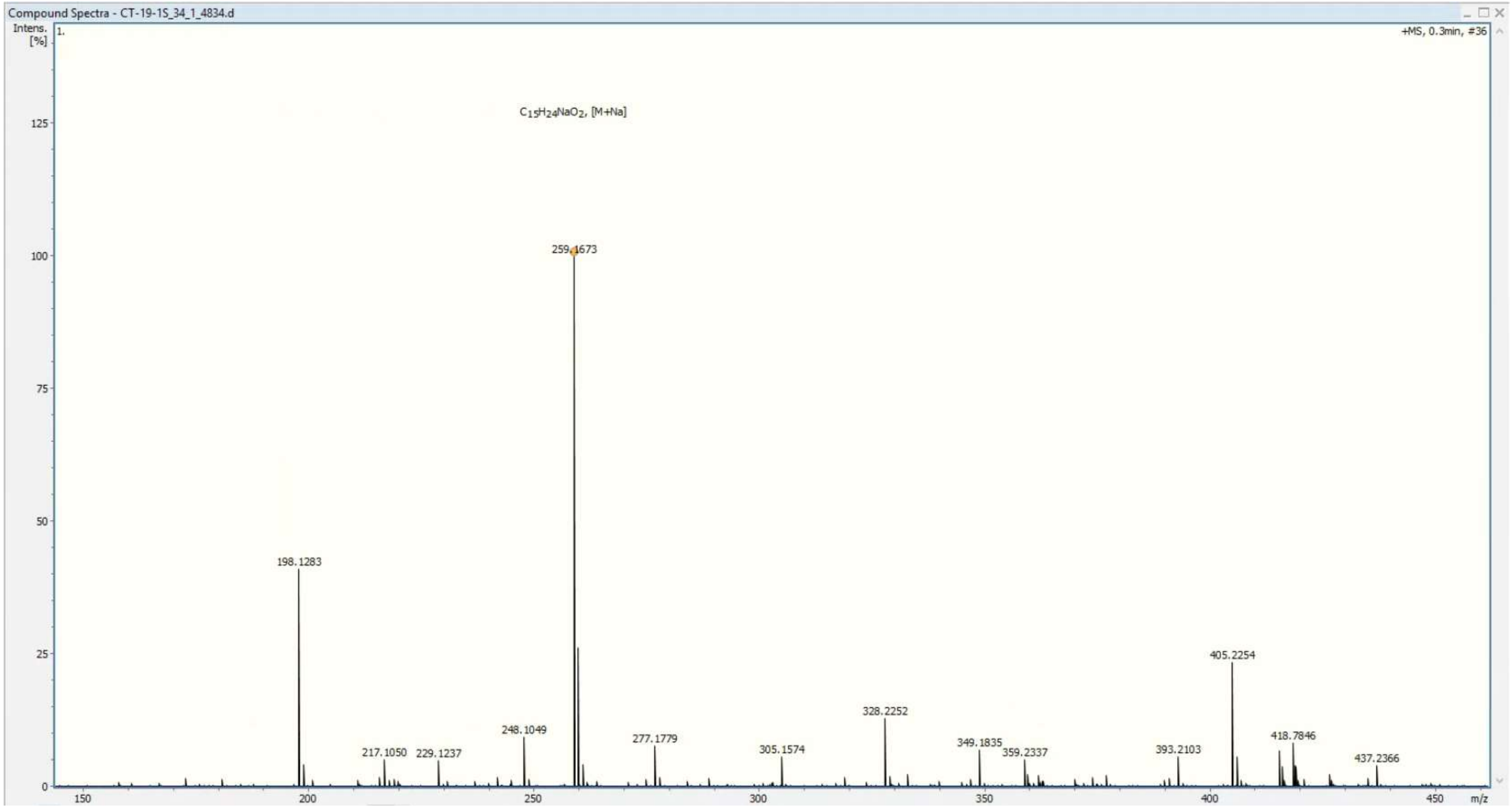

Figure S58. The (+)-HR-ESI-MS spectroscopic data of compound **5a**
